# High resolution respirometry to assess function of mitochondria in native homogenates of human heart muscle

**DOI:** 10.1371/journal.pone.0226142

**Published:** 2020-01-15

**Authors:** Adéla Krajčová, Tomáš Urban, David Megvinet, Petr Waldauf, Martin Balík, Jan Hlavička, Petr Budera, Libor Janoušek, Eva Pokorná, František Duška

**Affiliations:** 1 OXYLAB – Laboratory of Mitochondrial Physiology, Department of Anaesthesia and Intensive Care, Third Faculty of Medicine, Charles University and FNKV University Hospital, Prague, Czech Republic; 2 Department of Anaesthesia and Intensive Care, 1^st^ Medical Faculty, Charles University and General University Hospital, Prague, Czech Republic; 3 Department of Cardiac Surgery, Third Faculty of Medicine, Charles University and FNKV University Hospital, Prague, Czech Republic; 4 Transplantation Surgery Department, Institute for Clinical and Experimental Medicine, Prague, Czech Republic; 5 Department of Organ Recovery and Transplantation Databases, Institute for Clinical and Experimental Medicine, Prague, Czech Republic; Instituto de Investigacion Hospital 12 de Octubre, SPAIN

## Abstract

Impaired myocardial bioenergetics is a hallmark of many cardiac diseases. There is a need of a simple and reproducible method of assessment of mitochondrial function from small human myocardial tissue samples. In this study we adopted high-resolution respirometry to homogenates of fresh human cardiac muscle and compare it with isolated mitochondria. We used atria resected during cardiac surgery (n = 18) and atria and left ventricles from brain-dead organ donors (n = 12). The protocol we developed consisting of two-step homogenization and exposure of 2.5% homogenate in a respirometer to sequential addition of 2.5 mM malate, 15 mM glutamate, 2.5 mM ADP, 10 μM cytochrome c, 10 mM succinate, 2.5 μM oligomycin, 1.5 μM FCCP, 3.5 μM rotenone, 4 μM antimycin and 1 mM KCN or 100 mM Sodium Azide. We found a linear dependency of oxygen consumption on oxygen concentration. This technique requires < 20 mg of myocardium and the preparation of the sample takes <20 min. Mitochondria in the homogenate, as compared to subsarcolemmal and interfibrillar isolated mitochondria, have comparable or better preserved integrity of outer mitochondrial membrane (increase of respiration after addition of cytochrome c is up to 11.7±1.8% vs. 15.7±3.1%, p˂0.05 and 11.7±3.5%, p = 0.99, resp.) and better efficiency of oxidative phosphorylation (Respiratory Control Ratio = 3.65±0.5 vs. 3.04±0.27, p˂0.01 and 2.65±0.17, p˂0.0001, resp.). Results are reproducible with coefficient of variation between two duplicate measurements ≤8% for all indices. We found that whereas atrial myocardium contains less mitochondria than the ventricle, atrial bioenergetic profiles are comparable to left ventricle. In conclusion, high resolution respirometry has been adapted to homogenates of human cardiac muscle and shown to be reliable and reproducible.

## 1. Introduction

Human heart muscle has very high and continuous energy demand, mostly due to its contractile work when propelling blood through the circulatory system [1–3]. Myocardium can generate and consume 15 times its own weight of ATP each day[3], and over 95% of ATP is generated in oxidative phosphorylation[4]. Intracellular stores of ATP and creatine phosphate can be exhausted within a few seconds [4] and any defect in mitochondrial metabolism leading to decreased ATP production results in rapid contractile dysfunction [4,5]. Thus, cardiac function depends on the continuous ability of myocardium to generate ATP at a high rate [3,4]. Cardiac cells contain around 5 000–10 000 mitochondria per cell[6], occupying about 30% of the cellular volume[3]. The major fuel for oxidative metabolism of a healthy human heart are fatty acids (70–90%) [4,7], the remainder being carbohydrates, lactate, ketone bodies and amino acids [4]. A range of congenital and acquired heart diseases are associated with altered mitochondrial metabolism and insufficient ATP production[8,9], as is cardiac aging [10]. In addition, there is growing interest in the research of cardiotoxicity of commonly used drugs which may cause severe damage to cardiac mitochondria leading to arrhythmias or heart failure [11]. Most data are obtained by studying cell lines or animal models [12], but their applicability to humans is questionable.

Assessing function of human cardiac mitochondria in a way that would reflect conditions *in vivo* and allow *in vitro* experimental manipulation remains a challenge. Human cardiac tissue can be obtained during cardiac surgery or by catheter endomyocardial biopsy, which limits the size of the sample to hundreds of milligrams at best. This amount of tissue allows for spectrophotometric measurement of the activity of isolated mitochondrial complexes or other enzymes, but this only poorly reflects the function of mitochondria and its alteration (e.g. an existing influence may not be seen on the enzymes measured, or, on the other hand, a severe inhibition of a functionally redundant enzyme may not affect the function of mitochondria as a whole). High-resolution respirometry offers the advantage of assessing mitochondrial function in the cytosolic context, but the intracellular contents leaks after membrane disruption and this method has also other limitations. When isolated mitochondria are evaluated, large amounts of tissue are needed[13–16]. The preparation of permeabilised muscle fibres is manually demanding and results can be operator-dependent [17,18].

The main purpose of this study was to develop a simple, reproducible technique for *ex vivo* bioenergetics profiling of the human myocardial tissue. We hypothesised that high resolution respirometry of tissue homogenates, as originally described by Pecinova et al. [19] and later modified for the use in human skeletal muscle [20], can be successfully adopted to human cardiac muscle. We set ourselves following objectives: (i) Perform optimization of experimental such as homogenate concentration, (ii) determine durability of muscle samples, (iii) determine dependence of oxygen consumption rate on oxygen concentration, (iv) compare the new technique with well-established respirometry in isolated mitochondria and, lastly, (v) determine, whether atrial myocardium has energetic profile representative of left ventricle myocardium.

## 2. Methods

### 2.1. Study subjects

The study protocol and consent form were approved by the Ethics Committee of the Institute for Clinical and Experimental Medicine and Thomayer Hospital and the Ethics Committee of Faculty Hospital Kralovske Vinohrady, Prague. All the patients at Department of Cardiac Surgery provided written informed consent. The informed consent of brain-dead donors were obtained from family members.

Myocardial tissue homogenates were prepared from biopsies of right atrial appendages (sample ~ 300 mg) obtained from patients undergoing coronary artery bypass grafting surgery or heart valve replacement at the Department of Cardiac Surgery of Královské Vinohrady University Hospital (n = 57, for all experiments). For studies comparing atrial and ventricular myocardium, we used samples obtained from brain-dead organ donors (n = 15) without cardiac disease, whose hearts were not suitable for transplantation due to donor age over 50 years (as per local protocol of Transplantation Centre of Institute of Clinical and Experimental Medicine) or logistic issues. Tissue samples from left ventricle myocardium and from left atrial appendage were obtained by the organ retrieval surgeon immediately after cardiac arrest artificially induced by ice-cold KCl-based cardioplegic solution (NaCl 110.0 mM, NaHCO_3_ 10.0 mM, KCl 16.0 mM, MgCl_2_ 16.0 mM, CaCl_2_ 1.2 mM, pH 7.8). Patients with known heart disease (heart failure, severe arrhythmias incl. types II or III atrioventricular blocks, medical history of myocardial infarction, ejection fraction of left ventricle ˂ 30%, re-operation and past or current inflammatory heart diseases and mitochondrial disorders) were a priori excluded. Detailed characteristics of study subjects are described in Table 1.

**Table 1:** Characteristics of study subjects. Note: CAD = coronary artery disease, CABG = coronary artery bypass grafting, COPD = chronic obstructive pulmonary disease, CPR = cardiopulmonary resuscitation, MI = myocardial infarction, T1DM/T2DM = Diabetes Mellitus, type 1 or 2, respectively.

Subject	Age	Sex	Surgery procedure	Cardiac disease/Cause of brain death	Other diseases, medication
Cardiac surgery 1	69	M	CABG	CAD, MI	CKD, T2DM, Hypothyreosis
Cardiac surgery 2	64	M	CABG	CAD, MI	Atrial fibrillation, Hypertension, T2DM
Cardiac surgery 3	71	M	CABG	CAD	Arterial hypertension, Hyperlipidaemia
Cardiac surgery 4	75	M	CABG	CAD	Hypertension, T2DM, COPD
Cardiac surgery 5	71	M	CABG	CAD	Hypertension, Hyperlipidaemia
Cardiac surgery 6	56	F	CABG	CAD, MI, mild congestive heart failure	Hypertension, T1DM, Hyperlipidaemia, Hypothyreosis
Cardiac surgery 7	69	M	Aortic valve replacement	Aortic valve stenosis	CAD, Hypercholesterolaemia, Glaucoma
Cardiac surgery 8	74	M	CABG	CAD, MI	Atrial fibrillation, Diabetes mellitus type 2
Cardiac surgery 9	71	M	CABG	CAD, MI	Atrial fibrillation, Hypertension
Cardiac surgery 10	65	M	CABG	CAD, MI	Hypertension, T2DM, Rectal cancer in the past
Cardiac surgery 11	76	M	CABG	CAD	Hypertension, Hyperlipidaemia
Cardiac surgery 12	60	M	CABG	CAD	Hypertension, T2DM, Hyperlipidaemia
Cardiac surgery 13	68	F	CABG	CAD	T1DM, Hyperlipidaemia
Cardiac surgery 14	72	M	CABG	CAD	Atrial fibrillation, Hypertension, Hyperlipidaemia
Cardiac surgery 15	68	M	CABG	CAD	Hypertension
Cardiac surgery 16	54	M	CABG	CAD, MI	Hypertension, Hyperlipidaemia, Asthma bronchiale
Cardiac surgery 17	64	M	CABG	CAD	Hypertension, Hyperlipidaemia, Urinary bladder cancer
Cardiac surgery 18	65	M	Aortic valve replacement	Aortic valve stenosis with acute cardiac failure	Pulmonary hypertension, Arterial hypertension
Organ donor 1	56	F	-	Hypoxic brain damage after road traffic accident	-
Organ donor 2	71	F	-	Spontaneous intracerebral haemorrhage	Hypertension, Asthma bronchiale
Organ donor 3	54	F	-	Subarrachnoideal haemorrhage	Hypertension, Hypothyreosis
Organ donor 4	73	M	-	Spontaneous Intracerebral haemorrhage: hypertensive crisis	Arterial hypertension, T2DM
Organ donor 5	67	M	-	Traumatic brain injury	Hypertension
Organ donor 6	77	F	-	Subarrachnoideal haemorrhage	COPD, Hypertension
Organ donor 7	40	F	-	Hypoxic brain injury after CPR	Eosinophilic pulmonary disease
Organ donor 8	63	M	-	Subarrachnoideal haemorhage	Arterial hypertension
Organ donor 9	42	F	-	Hypoxic brain injury after CPR	Pulmonary embolism
Organ donor 10	45	M	-	Aneurysmal intracerebral hemorrhage	-
Organ donor 11	84	F	-	Spontaneous intracerebral haemorrhage	Arterial hypertension, Hyperlipidaemia
Organ donor 12	55	F	-	Hypoxic brain injury after CPR	Bacterial meningitis
Organ donor 13	62	F	-	Aneurysmal intracerebral hemorrhage	Chronic kidney disease
Organ donor 14	75	F	-	Aneurysmal intracerebral hemorrhage	-
Organ 15	78	F	-	Spontaneous intracerebral haemorrhage	Atrial fibrillation, Arterial hypertension, T1DM

### 2.2. Myocardial tissue homogenates preparation

**Fig 1:**
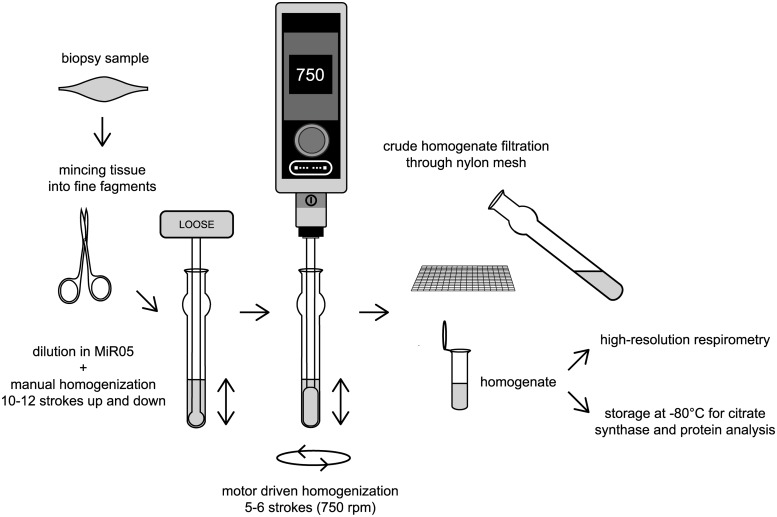
Step-by-step flowchart of cardiac muscle tissue homogenate preparation.

After collection, samples were put on ice in biopsy preservation solution [BIOPS, 2.77 mM CaK_2_EGTA, 7.23 mM K_2_EGTA, 5.77 mM Na_2_ATP, 6.56 mM MgCl_2_ x 6 H_2_O, 20 mM taurine, 15 mM Na_2_Phosphocreatine, 20 mM imidazole, 0.5 mM dithiothreitol and 50 mM MES hydrate (pH 7.1)] [21], and transported to the lab within 5 or 30 min (for cardiac surgery patients or organ donors, respectively). According to modified protocol for human skeletal muscle homogenates [20], we removed connective and adipose tissue using pre-cold scissors and anatomic forceps under a microscope (See photographs in the **Supplementary Appendix** and the **Supplementary Video File**), so that only muscle tissue was homogenized. After gentle blotting by a piece of sterile gauze (~ less than 6 seconds), cardiac muscle tissue was placed on parafilm, weighted on an analytical scale and minced with scissors into fine fragments whilst placed on ice. Tissue fragments were then scraped into pre-chilled 1 mL Dounce Tissue Grinder (glass construction; Wheaton^™^, Millville, USA) (see Fig 1) on ice. Respiratory medium MiR05 [0.5 mM EGTA, 3 mM MgCl_2_ x 6 H_2_O, 60 mM lactobionic acid, 20 mM taurine, 10 mM KH_2_PO_4_, 20 mM HEPES, 110 mM sucrose and 1 g/l of BSA (pH 7.1)][22] was subsequently added to the tissue to obtain a 10% tissue solution (~100 mg of tissue/per 1 mL of buffer).

Tissue fragments were then manually homogenized by 10–12 initial strokes up and down with a loose glass pestle (clearance = 0.114 ± 0.025 mm; Wheaton^™^, Millville, USA). The next step of homogenization in the Dounce grinder was performed with 5–6 slow strokes by a motor-driven pre-cold PTFE pestle of 2 mL Potter-Elvehjam homogenizer (rounded shape without notches; Wheaton^™^ 2mL Potter-Elvehjem Tissue Grinder; Wheaton^™^, Millville, USA; diameter shaft: 6.3 mm). The shaft of PTFE pestle was fixed in motor-driven homogenizer (HEi-Torque Value 100, Heidolph, Germany). Initially, the glass tube of 1 mL Dounce Tissue Grinder was inserted into plastic 50 mL Falcon tube filled with crushed ice. The PTFE pestle was immersed into the glass tube of 1 mL Dounce Tissue Grinder and motor-driven homogenizer was turned on. Whilst speed was increasing from 0 to 750 rpm, the pestle was slowly moved down to touch the bottom of the glass tube. When the speed reached 750 rpm, the pestle was manually moved up and down 5–6 times whilst it was still immersed in the homogenate in order to avoid bubble formation. After that, the speed was gradually slowed, and the pestle was pulled out of the glass grinder. The homogenate was then placed on ice and diluted in MiR05 buffer (from 1:1 to 1:9) to obtain the appropriate concentration for high-resolution respirometry and the homogenization was repeated by 3–4 additional strokes by motor-driven PTFE pestle. Raw homogenate was then filtered through a polyamide mesh (parameters: loop size 335 μm, fibre diameter 120 μm, open space 54%, material 100% polyamide; SILK & PROGRESS s.r.o., Czech Republic) in order to remove the remaining connective tissue, transferred to a pre-cold Eppendorf tube and stored on ice until the measurement. Various homogenizer sets with different clearance between the grinder and the pestle made from different material (glass & PTFE) were tried as well as single- versus multi-step homogenization procedure (see **Supplementary Appendix** for more details). We included the filtration through polyamide mesh to increase the homogeneity of muscle homogenate by removing the rest of connective tissue. We kept all the samples on ice throughout preparation procedures. All tubes, scissors and glassware were properly rinsed with 70% ethanol and distilled water, dried and kept on ice to cool to 0°C before use. Similarly, glass and PTFE pestles were pre-cooled to 0°C before homogenization. The critical step was to perform functional studies within an hour after homogenization procedure (see *Durability of homogenates* in Results).

### 2.3. High resolution respirometry

Functional studies were performed using high-resolution respirometry Oxygraph-2k (O2k, Oroboros Instruments, Innsbruck, Austria) with a polarographic oxygen electrode and two 2 mL chambers allowing for parallel measurements [13,21]. In our experiments, we performed all initial steps including calibration according to the manufacturer’s recommendations [21]. The oxygen solubility factor for MiR05 was set to 0.92 at 37°C[23]. The homogenate was then pipetted into both chambers, and these were carefully closed with stoppers avoiding the creation of bubbles in the medium. Oxygen concentration and flux were simultaneously recorded and analysed by Dat lab software (Datlab Version 7.0, Oroboros Instruments, Innsbruck, Austria). Reagents were added through a small capillary tube in the chamber using Hamilton syringes (Oroboros Instruments, Innsbruck, Austria).

### 2.4. Optimization of homogenate concentration

Initially, we aimed to develop a simple protocol (up to 30-minutes of duration) with well detectable responses of mitochondria to injected substrates, but avoiding a rapid exhaustion of oxygen in the chamber. A range of concentrations of homogenate (1; 2.5; 5 and 10%) were tried at n = 4. Lower concentrations of homogenates were prepared by dilution of 10% homogenate in additional volume of respiration medium (MiR05).

### 2.5. Substrate—Inhibitor—Uncoupler titration (SUIT) protocol

With 2.5% homogenate we subsequently performed a series of experiments to identify the most effective concentrations of adenosine diphosphate (ADP), the uncoupler “Carbonyl cyanide-4(trifluoromethoxy) phenylhydrazone (FCCP)”, the ATPase inhibitor oligomycin and cytochrome c (cyt c). Each titration experiment was performed on n = 7 except of oligomycin experiments on FCCP-induced stimulated respiration, which was performed on n = 3. All reagents were obtained from Sigma-Aldrich (St. Louis, USA). Substrates were dissolved in distilled water and pH of each solution was adjusted to pH 7.0. Oligomycin, FCCP and antimycin A were dissolved in DMSO. Firstly, we made a number of measurements with increasing ADP concentration (0.5; 1; 1.5; 2.5 mM) to achieve a maximum increase of oxygen consumption rate. ADP was added after injection of substrates for complex I (malate + glutamate). After that, oligomycin was titrated at various concentrations (0.5; 1.0; 1.5; 2; 2.5 μM) to decrease oxygen flux until the curve achieved the plateau. A series of experiments has been performed with FCCP titration to find optimal concentration for maximal respiratory rate, i.e. full uncoupling without electron transfer system (ETS) inhibition. FCCP was injected in stepwise manner to achieve final concentrations of 0.25; 0.5; 0,75; 1; 1.25; 1.5 and 1.75 μM, and peak oxygen consumption rate was sought for. In addition, we tested a possible interaction between 2.5 and 0.5 μM oligomycin and FCCP as this problem has recently been discussed [24]. Finally, oxygen consumption was recorded at increasing concentrations of cytochrome c (10 and 20 μM).

### 2.6. Calculation of mitochondrial functional indices

**Fig 2:**
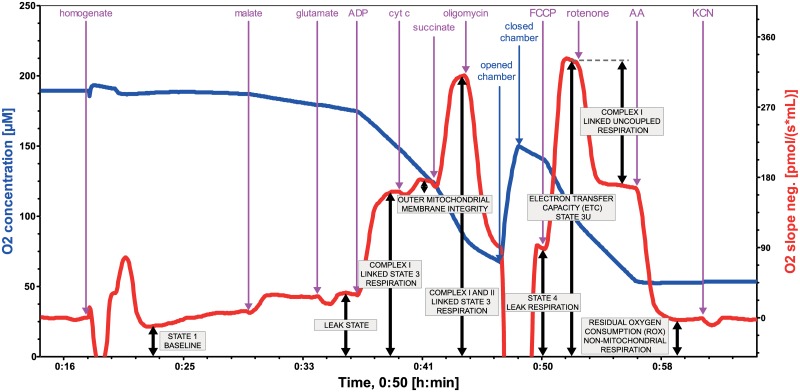
Mitochondrial functional indices measured by high-resolution respirometry on 2.5% homogenate of human cardiac muscle (left ventricle). Bioenergetic parameters were assessed at baseline (State 1) and by sequential addition of 2.5 mM malate and 15 mM glutamate (so-called pseudo-State 2 respiration representing LEAK state), 2.5 mM ADP (complex I linked State 3 respiration), 10 μM cytochrome c (outer mitochondrial membrane integrity), 10 mM succinate (complex I+II linked State 3 respiration), 2.5 μM oligomycin (State 4), 1.5 μM FCCP (State 3u representing electron transfer capacity), 3.5 μM rotenone (complex I linked uncoupled respiration), 4 μM antimycin A (residual oxygen consumption representing non-mitochondrial respiration) and 1 mM KCN (to confirm that antimycin A provides an adequate non-mitochondrial background). Each concentration refers to final concentration of the agent in the chamber. Stepwise titration of the uncoupler is possible and recommended if time permits. We also verified that 2.5 μM oligomycin does not inhibit uncoupled respiration as compared to 0.5 μM oligomycin. Note: AA = antimycin A; ADP Mg = Adenosine diphosphate with Mg^2+^; Cyt c = cytochrome c; FCCP = uncoupler; oligo = oligomycin (F_1_F_O_ATPase inhibitor); KCN = potassium cyanide.

Respiratory states and substrate concentrations used for final SUIT protocol are outlined in Fig 2. Mitochondrial parameters were derived as shown in Table 2.

**Table 2:** Mitochondrial functional indices.

Parameter (Abbreviation)	Name	Measured/Calculated	Note
STATE 1 [pmol/(s*ml)]	Basal respiration	Measured as OCR after addition of homogenate/mitochondria minus ROX	Represents basal respiration
*%* increase post addition of cyt c	-	Measured as 100 multiplied by (OCR after addition of substrates for complex I, ADP and cytochrome c minus OCR after addition of substrates for complex I, ADP)/divided by OCR after addition of substrates for complex I, ADP and cytochrome c	Represents damage of outer mitochondrial membrane
STATE 3 = OXPHOS CAPACITY (P’) [pmol/(s*ml)]	Oxidative phosphorylation capacity	Measured as OCR after addition of substrates for CI, ADP, cyt c and substrate for CII minus ROX	Represents OXPHOS capacity
STATE 4 = leak respiration (L’) [pmol/(s*ml)]	Leak respiration with adenylate	Measured as OCR after oligomycin addition minus ROX	Represents proton leak (in absolute values)
STATE 3u = ETS CAPACITY [pmol/(s*ml)]	Electron transfer system capacity	Measured as OCR after addition of substrates for CI and II, ADP, cyt c, oligomycin and FCCP minus ROX	Represents electron transfer system capacity
ROX [pmol/(s*ml)]	Residual oxygen consumption rate	Measured as OCR after addition of antimycin A	Represents non-mitochondrial respiration
Complex I [pmol/(s*ml)]	-	Measured as OCR after addition of malate+glutamate and ADP minus ROX	Represents complex I linked respiration
CI control ratio (CI/CI+II)	Complex I control ratio	Measured as OCR after addition of malate+glutamate and ADP/ devided by (this value + OCR value after subsequent succinate addition)	Represents control ratio for NADH electron transfer-pathway state
Complex II [pmol/(s*ml)]	-	Measured as OCR after addition of malate+glutamate, ADP, cyt c and succinate minus OCR after addition of malate+glutamate, ADP and cyt c	Represents complex II linked respiration
CII control ratio (CII/CI+II)	Complex II control ratio	Measured as OCR after addition of malate+glutamate, ADP, cyt c and succinate/ devided by (this value plus OCR after malate+glutamate and ADP addition)	Represents control ratio for succinate electron transfer-pathway state
Proton leak [%]	-	Measured as (100 multiplied by OCR after addition of substrates for CI and CII, ADP, cyt c and *oligomycin*)/ divided by OCR after addition of substrates for CI and CII, ADP and cyt c	Represents proton leak (%)
ATP production [%]	Adenosine triphosphate production	Measured as 100 minus value of proton leak (%)	Represents the rate of mitochondrial ATP production (%)
RCR	Respiratory control ratio	Calculated as the ratio of STATE 3/STATE 4	Represents OXPHOS coupling efficiency

Note: ADP = Adenosine diphosphate; ATP = Adenosine triphosphate; CI = complex I; CII = complex II; FCCP = Carbonyl cyanide-4-(trifluoromethoxy)phenylhydrazone; OCR = oxygen consumption rate; OXPHOS = oxidative phosphorylation; RCR = respiratory control ratio; ROX = residual oxygen consumption.

### 2.7. Mitochondrial membrane integrity

**Fig 3:**
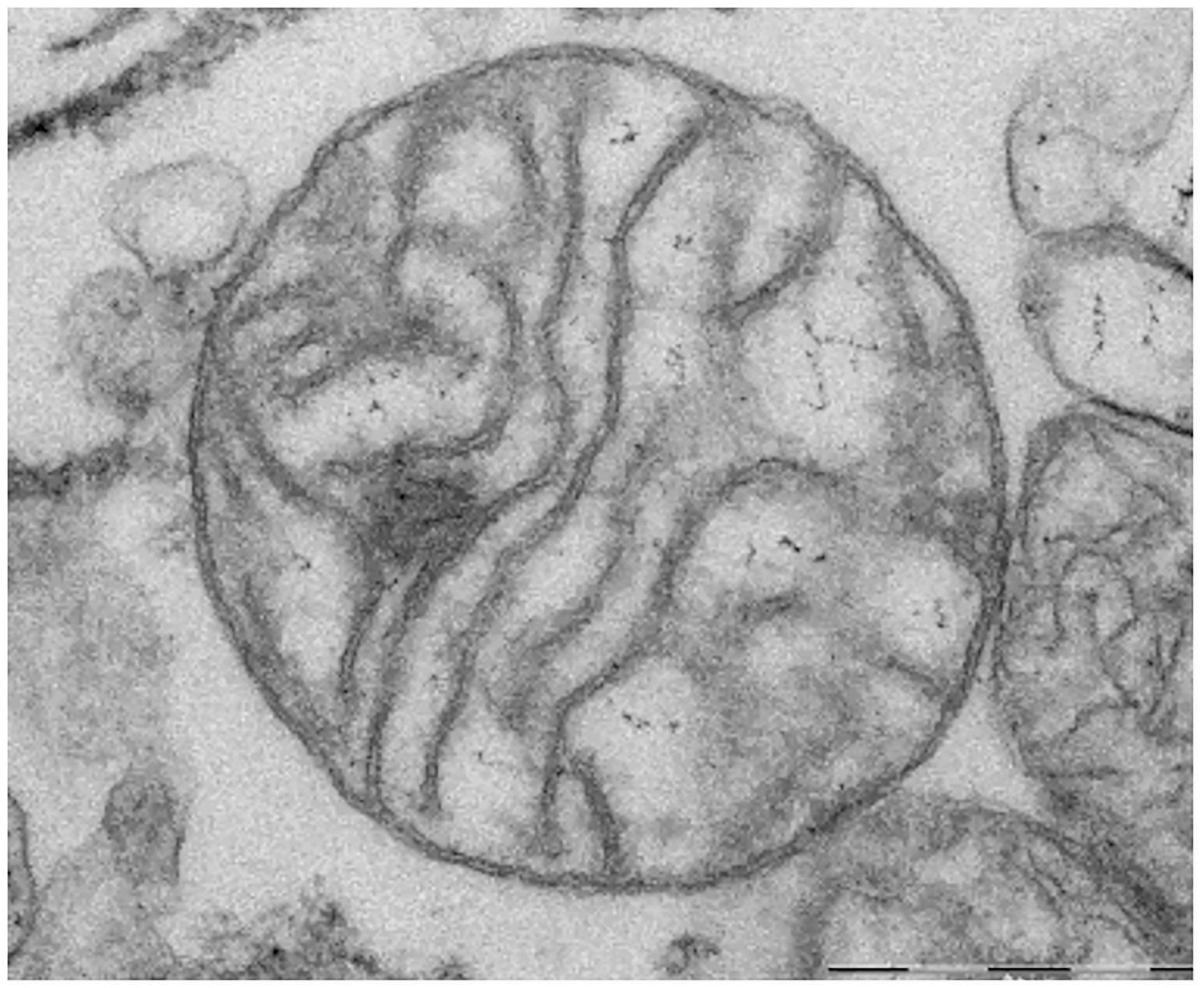
Detail of intact mitochondria in human heart muscle homogenate (from left ventricle) on electron microscopy. Scale bar: 0.5 μm.

Mitochondrial outer membrane integrity was tested by addition of 10 μM cytochrome c. Since an intact outer mitochondrial membrane provides a complete barrier against penetration of cytochrome c, an increase in respiration after addition of exogenous cytochrome c reflects disruption of outer mitochondrial membrane caused by homogenization and the isolation procedure[25]. Up to a 10–20% increase in respiration is considered acceptable as evidence of preserved integrity of mitochondrial membrane[26,27]. Damage to outer mitochondrial membrane was defined as percent of increase in oxygen consumption after addition of exogenous cytochrome c, when both ADP and mitochondrial substrates were present, and calculated as 100*(cyt c-ADP)/ADP. The *respiratory control ratio* (State 3/State 4), indicating mitochondrial coupling of respiration to phosphorylation, was used as an additional parameter testing functional integrity of mitochondria[26,28]. The morphology of mitochondria in the homogenate was assessed by electron microscopy using FEI Morgagni 268 transmission electron microscope (see Fig 3). The images were captured by Mega View III CCD camera (Olympus Soft Imaging Solutions). More details about preparation of mitochondria for electron microscopy and imaging are described in **Supplementary Appendix**.

### 2.8. Comparison of cardiac muscle homogenates with isolated mitochondria and reproducibility of the method

We compared the reproducibility and results of the new respirometry protocol using homogenates with isolated mitochondria, the gold standard to study mitochondrial function[29]. Muscle samples from the left ventricles of brain-dead organ donors (n = 4) were split and processed: one part used for preparation of a homogenate and the other for the preparation of mitochondria. Respirometry in homogenates and isolated mitochondria was done in parallel, using the same SUIT protocol. The same process was repeated twice for each subject in order to test the reproducibility of the results. Isolation of both subsarcolemmal and interfibrillar mitochondria was performed in parallel with preparation of cardiac muscle tissue homogenates as previously described[19,28]. Briefly, minced cardiac tissue was homogenized in Isolation Medium containing 225 mM Mannitol, 75 mM Sucrose and 1 mM EGTA (pH 7.4) and subsequently centrifuged at low speed. Subsarcolemmal mitochondrial fraction was obtained from supernatant followed by centrifugation at high speed without the use of proteolytic enzymes. Interfibrillar mitochondria were isolated by multi-step centrifugation after the initial pellet had been treated with nagarse (see **Supplementary Appendix** for more details). Mitochondrial yield was calculated as the % of citrate synthase activity in isolated mitochondria compared to whole homogenate[13] and was 13 ± 4% of total activity in the homogenate. To test the reproducibility of both methods, from duplicate measurements we calculated coefficient of variability (CV). To assess the reproducibility of the technique on atrial homogenates, we performed duplicate parallel measurements of homogenates. Each pair of homogenates was prepared from the same biopsy obtained from the patients undergoing cardiac surgery (n = 9) and also calculated CV.

### 2.9. Atrial vs. ventricular samples

Samples from brain-dead organ donors (n = 9) were obtained from the left appendage and ventricle of the same heart, processed in parallel by the same protocol and measured simultaneously in the two respirometry chambers. Abundant tissue obtained from organ donors allowed us to perform these measurements in duplicates or triplicates. Whilst doing so, we stored the intact heart muscle sample on ice and repeated the process of homogenisation immediately prior to the measurements.

### 2.10. Citrate synthase activity

Data were normalized to citrate synthase activity which is routinely used as a marker of mitochondrial density in the sample[30]. Immediately after the filtration step, the homogenized sample was divided into the two equal parts containing the same amount of homogenate: one part was subsequently used for high resolution respirometry and the second part was taken and stored at -80°C until the citrate synthase analysis. Citrate synthase assay was then performed as previously described[30].

### 2.11. Durability tests and cryopreservation experiments

#### Durability of muscle samples

The atrial samples from brain-dead donors (n = 3) were divided into 5 parts and stored on melting ice in a fridge (0–2°C). After 0, 12, 24, 48 and 72 hours one randomly selected part of the sample was homogenised and the respirometry protocol performed immediately afterwards.

#### Durability of tissue homogenates

In analogy, we prepared homogenates from fresh heart samples of brain-dead donors (n = 4), divided into aliquots, measured at baseline, and then 2, 4, 6, 12 and 24 hours after homogenate storage on ice. Results in time were compared to determine the influence of storage time on mitochondrial functional indices, particularly coupled and uncoupled ETS capacity, and leak through inner mitochondrial membrane.

#### Cryopreservation of homogenates and cardiac muscle samples

Homogenate was prepared in MiR05 and frozen at– 80°C (n = 3; each sample was prepared from the individual cardiac muscle biopsy). Analogously, cardiac muscle samples (n = 3) in BIOPS with 30% DMSO and 10 mg/ml BSA were frozen and stored in liquid nitrogen as previously described[31]. Later the muscle was thawed, washed and homogenized in MiR05. In both experiments, high resolution respirometry (SUIT protocol) was performed at baseline (before freezing) and immediately after thawing.

### 2.12. Sensitivity of detection of complex I inhibition

During preliminary experiments (n = 3; each sample was prepared from the individual cardiac muscle biopsy), we first found that a concentration 0.003 μM of rotenone decreases complex I linked respiration by 39±5%. In order to assess whether our SUIT protocol is able to detect such a degree of complex I inhibition, in parallel chambers we tested the effect of addition of 0.003 μM of rotenone to Complex I substrate (malate+glutamate) and ADP in the presence or absence of complex II substrates (succinate).

### 2.13. Dependence of oxygen consumption rate on oxygen concentration

Human atrial homogenates (n = 7; each sample was prepared from the individual cardiac muscle biopsy) were observed after injection of substrates (2.5 mM malate, 15 mM glutamate, 10 mM succinate and 10 μM cytochrome c) and uncoupler (1.5 μM FCCP) until the oxygen concentration was completely exhausted. Afterwards, the chamber was opened and oxygen consumption measured again to ensure that energy substrates were not depleted during the experiment and oxygen concentration was the only factor influencing oxygen consumption rate.

### 2.14. Statistics

Continuous data are presented as means ± SD. Coefficient of Variation [CV] was calculated as CV [%] = 100 * SD/mean, where SD is standard deviation. Raw respirometry parameter data were modelled using linear mixed effect model (LMEM). In the fixed part, the model consists of a dependent continuous parameter (e.g. oxygen consumption rate) and a categorical independent parameter (e.g. substrates, inhibitors or time intervals [in durability analysis]). In the random part, there was a random intercept (ID of a patient) and correlation matrix of residuals, if statistically significant (as assessed by likelihood ratio test). LMEM also was used for fitting linear part of relationship between normalized O_2_ consumption and O_2_ concentration. Here we use random intercept (ID of a patient) and random coefficient (O_2_ concentration). Models were fit by maximum likelihood method. P value <0.05 was considered significant. For all statistical analyses we used software Stata 15.1 (Stata Corp., LLC, U.S.A.).

## 3. Results

### Final protocol

#### Preparation of homogenates

Homogenization is the crucial step, as too thorough homogenisation disrupts mitochondrial membranes and too gentle procedure leads to an inhomogeneous sample. More detailed results and discussion on homogenization are in **Supplementary appendix**. The best results were obtained by a two-step homogenization process. First, minced tissue fragments were diluted in MiR05 buffer to obtain a 10% homogenate and manually homogenized by 10–12 strokes up and down using a Dounce tissue grinder set with a clearance 0.114 ± 0.025 mm. Second, 5–6 slow strokes were delivered at 750 rpm by motor-driven PTFE pestle (2 mL Potter-Elvehjam homogenizer) placed in the same glass tube. The sample was filtered through a polyamide mesh before measurements (See **Supplementary Video File** showing homogenization procedure).

#### Final SUIT protocol

Titration experiments (See **Supplementary Appendix**) resulted in the final protocol: Initially, 1900 μl MiR05 was pipetted to the chamber. After calibration, 200 μl of 2.5% homogenate from either atrial or ventricular myocardium (~5 mg myocardial tissue wet weight homogenized in 200 μL of MiR05) was added into each chamber and the lid was closed. Bioenergetic parameters were assessed by standard sequence of substrates, inhibitors and the uncoupler (See Fig 2).

### Comparison of homogenates with isolated mitochondria prepared from ventricular myocardium

Mitochondrial structure in homogenates seemed intact under electron microscopy (see Fig 3). Mitochondrial membranes tend to be better preserved in homogenates as compared to isolated mitochondria. The increase of oxygen consumption after addition of cytochrome c (an index of mitochondrial outer membrane damage) was 11.7±1.8% in homogenates vs. 15.7±3.1% (p˂0.05) and 11.7±3.5% (p = 0.99) in subsarcolemmal and interfibrillar isolated mitochondria, respectively. Respiratory control ratio (an index of efficiency of OXPHOS) was 3.65±0.5 in homogenates vs. 3.04±0.27 (p˂0.01) and 2.65±0.17 (p˂0.0001) in subsarcolemmal and interfibrillar isolated mitochondria, respectively. OXPHOS and ETS capacity normalized to CS activity were not significantly changed in homogenates compared to isolated mitochondria (OXPHOS/CS 8239±5374 vs. 7985±2504, p = 0.88; ETS/CS 7535±4545 vs. 6777±2174, resp., p = 0.60). Duplicate measurements of all dimensionless mitochondrial parameters had lower coefficient of variability in homogenates as compared to isolated mitochondria (CV ≤ 4% vs. 20%; see S1 Table in S1 Appendix for more detailed information).

Similar coefficients of variation were obtained for mitochondrial functional indices obtained by duplicate measurements of homogenates prepared from identical samples of right atrial appendage. See Table 3.

**Table 3:** Raw data from 6 duplicate measurements (homogenate A vs homogenate B) from right atrial appendages. Mitochondrial functional parameters. Note: ETS = Electron Transfer System, OXPHOS = oxidative phosphorylation, RCR = Respiratory Control Ratio, CI = Complex I, CII = Complex II, ROX = Residual Oxygen Consumption, ET = Electron Transfer.

Biopsy	Sample 1		Sample 2		Sample 3		Sample 4		Sample 5		Sample 6		Mean ± SD	Mean CV[%] ± SD
Homogenate	A	B	A	B	A	B	A	B	A	B	A	B		
STATE 1 [pmol/(s*ml)]	7	7	9	9	8	8	9	8	5	5	7	6	**7±2**	**5.4±3.9**
*%* increase post addition of cyt c	16	15	16	16	17	18	15	16	15	17	11	13	**15±2**	**5.4±3.3**
STATE 3 = OXPHOS CAPACITY (P') [pmol/(s*ml)]	181	195	223	249	234	209	216	212	216	186	167	145	**203±29**	**7.2±3.4**
STATE 4 = leak respiration (L') [pmol/(s*ml)]	51	59	82	81	77	72	74	76	74	67	52	51	**68±12**	**4.2±3.7**
ROX [pmol/(s*ml)]	1	0	-3	-4	-3	-2	-3	-2	-1	1	1	3	**N/A**	
Complex I [pmol/(s*ml)]	89	92	108	115	105	89	100	97	97	82	85	68	**94±13**	**8±6**
CI control ratio (CI/CII+II)	0.55	0.51	0.52	0.50	0.49	0.46	0.49	0.49	0.48	0.49	0.55	0.52	**0.5±0.03**	**2.8±2.1**
Complex II (CII) [pmol/(s*ml)]	72	87	98	116	111	103	102	100	104	86	70	64	**93±17**	**8.7±4.8**
CII control ratio (CII/CI+II)	0.45	0.49	0.48	0.50	0.51	0.54	0.51	0.51	0.52	0.51	0.45	0.48	**0.5±0.03**	**3.0±2.4**
Proton leak [%]	28	30	37	33	33	34	34	36	34	36	31	35	**33±2.54**	**5.0±4.5**
ATP production [%]	72	70	63	67	67	66	66	64	66	64	69	65	**66.5±2.64**	**5.0±2.7**
STATE 3u = ET capacity (E') [pmol/(s*ml)]	196	206	227	260	248	217	210	197	210	180	157	133	**203.4±35.39**	**2.5±1.4**
RCR	3.84	3.51	2.74	3.15	3.18	2.99	2.79	2.57	2.81	2.71	3.04	2.69	**3.00±0.37**	**6.3±2.7**

### Durability and cryopreservation experiments

Serial measurements of atrial myocardium samples stored in transport media at 0–4°C demonstrated preserved mitochondrial functional parameters (unchanged uncoupling and ETS >92% of baseline) for up to 12 hours. Afterwards we observed a steady decline of respiratory capacity and increased uncoupling. After 24, 48 and 72 hours, uncoupled respiration dropped to 87% ± 18%, 81% ± 20% and 60 ± 44%, respectively. In contrast, respiration attributable to leak of protons through inner mitochondrial membrane increased steadily from 33% to 90% after 72 hours. If homogenate was prepared first and then stored on ice, the signs of decay occurred earlier, within 2–4 hours. ETS gradually declined over time, and dropped to 89±7%, 93±1%, 63±29%, 52±27% after 2, 4, 6, and 12 hours. Proton leak increased steadily to almost double in 12 and 24 hours after homogenization. Cryopreservation of both homogenates or intact muscle resulted in damage and uncoupling of both mitochondrial membranes and 80% resp. 44% decrease in Complex I-linked respiration. (See **Supplementary Appendix**).

### Representativeness of atrial appendage samples—Comparison with ventricular myocardium

**Fig 4:**
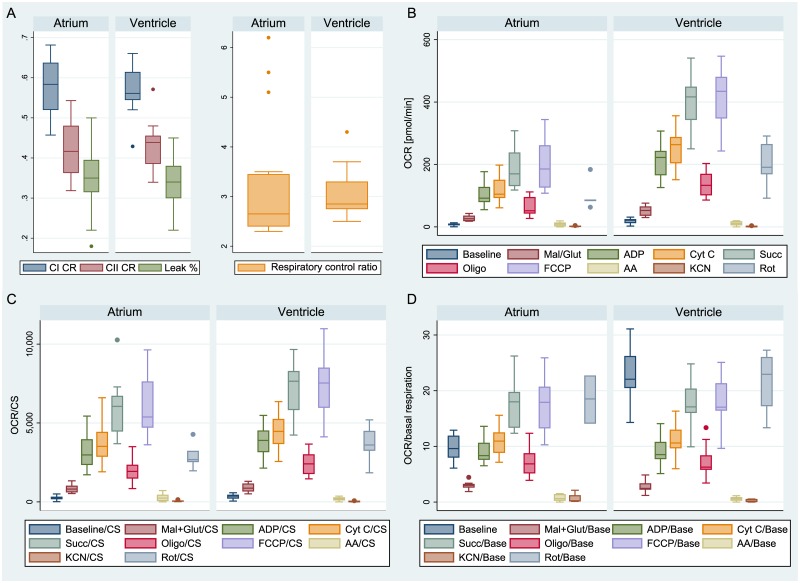
Comparison of atrial and ventricular mitochondrial functional indices (left atrium; left ventricle). A) Dimensionless functional indices. B) Raw data (absolute values). C) Values corrected to citrate synthase activity. D) Values corrected to baseline respiration.

**Fig 5:**
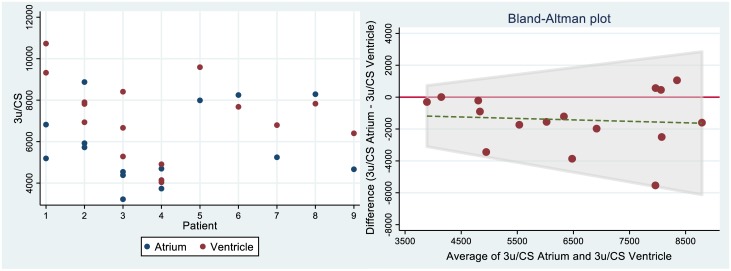
A-left: Dot-plot of interclass variability of electron transfer capacity normalised to citrate synthase activity (Denoted as state 3u = uncoupled). B-right: Bland-Altman plot showing the individual differences in the same parameter between atria and ventricles. Ventricles have generally a bit higher 3u/CS than atria, but there is no dependency on absolute value.

When samples of atrial appendage were compared with myocardium from the apex of left ventricle of the same subject (n = 16 pairs from 9 hearts) and processed through the same protocol, no significant differences were found in any of the derived dimensionless mitochondrial functional indices (proton leak 35±9% vs. 34±6%, p = 0.794; RCR 3.2±1.3 vs. 3.1±0.5, p = 0.258; CI control ratio 0.58±0.07 vs. 0.57±0.06, p = 0.493; CII control ratio 0.42±0.07 vs. 0.43±0.06, p = 0.503 in atrial appendages and ventricles respectively; see Fig 4A). The absolute mitochondrial indices in ventricular samples were ~ 2 fold higher (n = 5 pairs from 5 hearts; see Fig 4B). The values are still ~ 1.2 fold higher when normalized to citrate synthase activity (see Fig 4C), but the differences disappear completely when oxygen consumption rate is corrected to baseline value (see Fig 4D). Most importantly, as demonstrated in the dot plots in Fig 5A, when respirometry measurements are performed in the homogenates of atrial and ventricular samples, the physiological variability among patients is higher than the variability between atrial vs. ventricular samples or the variability between duplicate measurements. In addition, Bland Altman plot (see Fig 5B), demonstrates the absence of any pattern of the differences between ETS in atria and ventricles.

### Sensitivity of detection of complex I inhibition

We found a decrease by 87±14 pmol/(s*ml) vs. 71±17 pmol/(s*ml) [82%] in the chamber with substrates for complex I only vs. chamber with full SUIT protocol (i.e. substrates for both complexes I and II). Thus, <40% decrease in Complex I linked respiration is still detectable even in the presence of abundant substrate of complex II.

### Influence of oxygen concentration on uncoupled respiration

In fully uncoupled mitochondria, we observed a quasi-hyperbolic relationship between oxygen concentration and consumption (See Supplementary Appendix). Firstly, there is a linear portion of the curve with mean slope 0.205 (CI95 0.138–0.272). Then, below ~20 μM of oxygen there was a very steep decline of respiration.

### Possible extension of basic respirometry protocol—Measuring respiratory fluxes through individual ETS complexes and fatty acid oxidation

By modifying the protocol so that specific inhibitors are added in the presence of abundant substrate and ADP, the technique enables the measurement of the complex II-linked uncoupled respiration (by addition of inhibitor = malonate 5 mM in uncoupled state instead of rotenone) or complex IV (by addition of substrate = ascorbate/TMPD 10/0.2 mM and inhibitor = 100 mM Sodium Azide), or fatty acid oxidation (by addition of substrate = palmitoyl-carnitine 20 μM and FAO inhibitor = etomoxir 40 μM). For more details See **Supplementary Appendix**.

## 4. Discussion

In this study, we adopted high resolution respirometry to homogenates of human atrial myocardium to enable measurement of mitochondrial function from a sample of less than 20 mg of fresh muscle. We demonstrated that this simple technique (transport media-to-respirometer time <20 min) has better variability and the same or better preservation of outer membrane integrity and OXPHOS coupling efficiency than the established techniques of respirometry in isolated mitochondria [11,13,24,42]. The method has been shown to be sensitive enough to detect <40% inhibition of Complex I-linked respiration. We also have shown that bioenergetic parameters of atrial myocardium are representative of left ventricle of the same heart provided that the adjustment to mitochondrial content is made. Crucial to the reliability of the technique is avoiding hypoxia and when parallel measurements are made (e.g. experimental vs. control), both measurements should be performed at similar oxygen concentration. Notably, 10% decrease in oxygen concentration causes ~2% decrease of oxygen consumption rate–this phenomenon is also observed with permeabilised fibers (See Pesta et al. 2012[21]). Thus, when oxygen concentrations in the two chambers start to be different, we recommend opening both chambers and allow equilibration with ambient air. Although respiratory function declines slowly, it is possible to store the muscle sample on ice up to 12 hours prior to homogenization and measurement. Once homogenized, the respirometry must be performed immediately as small, but significant decreases occur as early as 2 hours after homogenization. We have not found any successful cryopreservation technique, and it is necessary to process samples with 12 hours of sampling.

Mitochondrial function in the human heart has conventionally been assessed by polarographic measurement performed on isolated mitochondria[32–35], using a classical Clark electrode[36]. For this technique a minimum of ~0.5 g is required [35]. Such an amount of tissue can only be obtained from brain dead donor hearts unsuitable for donation (similar to what we did in this study), or explanted failing hearts, which are known to be dysfunctional[8,34,37]. Moreover, the isolation procedure itself can lead to artificial damage of mitochondria during centrifugation[38], which only yields 20–40% of mitochondria in the sample[13–16,39], potentially inducing a selection bias[40]. Because isolation of mitochondria disrupts native environment of mitochondrial network and intracellular communication with other organelles[41], the generalizability of the results to in vivo environment have been questioned [29,42]. In 1987, Veksler et al.[43] described the technique of permeabilisation of cardiac muscle fibers by saponin, which interacts with cholesterol and creates pores in plasma membrane which becomes permeable for small molecules (such as for ADP, or other substrates and inhibitors needed in SUIT respirometry protocol), so that cytosol is washed out from otherwise intact cells[17,44]. This *in situ* technique is therefore more representative of *in vivo* mitochondrial arrangement than isolated mitochondria and allows determination of mitochondrial functional parameters in small endomyocardial biopsy samples[43,45,46] stored for up to 24 hours[17]. Major disadvantage of permeabilisation is that the process is manually demanding and takes at least ~2 hours[17]. Due to the challenges of assessing citrate synthase activity from permeabilised fibers recovered from the respirometry chamber [17,18], mitochondrial functional indices were normalized to wet weight of manually isolated fibers blotted on a filter paper to remove excess of fluid[17,18]. A recent study showed a relatively large variability (CV of 15.2%) between the measurements of two bundles of skeletal muscle fibers from the same subject[18], roughly the same variability as we have seen in isolated mitochondria in our study, and twice the variability we have seen by using homogenates in this study.

Another factor, which has to be considered when comparing experimental approaches to the assessment of mitochondrial bioenergetics is the degree of damage mitochondria during the pre-analytical phase. The increase of ADP-driven respiration after the addition of cytochrome c was 15±2% with our technique, suggesting acceptable integrity of outer mitochondrial membrane. The leak of cytochrome c through damaged outer mitochondrial leads to an increase of respiration over 20%[26,27]. The damage of inner mitochondrial membrane causes uncoupling of oxidative phosphorylation from ETS. With our technique, respiratory control ratio (see Table 3) was 3.0±0.4 a bit lower than in human permeabilised fibres of myocardium (5.0±0.4)[47] and skeletal muscle[48]. It remains unclear, what is causing the artificial uncoupling, but it should be noted that it was not seen in fresh homogenates of human skeletal muscle, where RCR was 7.8[20] nor it was observed in saponin-permeabilised cardiac fibres harvested by very similar methods[47]. Myosin-ATPases are known to increase the apparent KM for ADP[49]. Of note, oxygen flux after addition of oligomycin is leak + non-mitochondrial oxygen consumption. Although non-mitochondrial oxygen consumption was close to 0 in our experiments (see Table 3), we recommend routine addition of ultimate ETS inhibitor, that allows determination of non-mitochondrial oxygen consumption. In case it is elevated, leak respiration should be determined by subtracting it from oxygen flux after oligomycin.

In practice, using high resolution respirometry on cardiac muscle homogenates enables *ex vivo* interventional studies (e.g. manipulations with substrate environment or exposure to drugs such as antiarrhytmics propafenone or amiodaron, which was the original purpose, why we aimed to develop this method), using e.g. atrial appendages resected during the insertion of extracorporeal circuit cannula during cardiac surgery. This is a risk-free source of heart muscle for research, in contrast to endomyocardial biopsy[45], and the simplicity of the technique together with durability of the sample 12 hrs allows for repeated measurements from the same atrium. In line with the finding of Lemieux et al.[47], we found that bioenergetics profile of atrial myocardium reflects that of ventricular myocardium, when adjusted to increased mitochondrial content. Indeed, the ability to study specific activities of individual ETS complexes is yet another advantage of this method, e.g. for further research in the field of mitochondrial myopathies.

The major weakness of our study is relatively limited number of brain-dead organ donors in which we were able to compare variability between atria and ventricles (n = 12). Based on post-hoc power analysis (data not shown), this number of subjects would only allow to detect differences in the main mitochondrial parameters in the range >20 to >30% and did not allow us to look at various subgroups of patients with a range of cardiac diseases, nor allowed a robust multiple regression analysis looking at factors influencing the bioenergetic differences between atrial and ventricular myocardium. Indeed, there are no operations on healthy hearts and underlying diseases leading to cardiac surgery may have affected mitochondrial function[45]. The technique itself has the main limitation in the degree of artificial uncoupling of the inner mitochondrial membrane.

In conclusion, we have (i) adapted homogenization procedure and high resolution respirometry technique for small samples of human myocardium, (ii) determined that whilst tissue samples can be stored for up to 12 hours, homogenates are to be processed within 2 hours. We have shown (iii), that there is an oxygen limitation requiring oxygen fluxes be adjusted to oxygen concentration or experiments in parallel chambers be conducted at identical O_2_ concentration. When compared with respirometry in isolated mitochondria (iv) the new technique requires ~10 times smaller sample, is less time consuming, more reproducible, but leads to some degree of artificial uncoupling. Lastly (v), we have determined, that atrial myocardium has energetic profile representative of left ventricle myocardium.

## Supporting information

S1 Video (AVI)

S1 Appendix (DOCX)

S1 Data (ZIP)
