# Supplementary material for: High resolution respirometry to assess function of mitochondria in native homogenates of human heart muscle
Source: PLoS One. 2020 Jan 15;15(1):e0226142. doi: 10.1371/journal.pone.0226142 (PMC6961865; doi:10.1371/journal.pone.0226142)
Supplement: S1 Appendix — (DOCX) [file pone.0226142.s002.docx]

SUPPLEMENTARY APPENDIX

to paper Krajcova A et al. “High resolution respirometry to assess functions of mitochondria in native homogenates of human heart muscle“

Table of Contents

[LIST OF ABBREVIATIONS: 2](#_Toc4875671)

[LIST OF CHEMICALS: 4](#_Toc4875672)

OPTIMIZATION OF HOMOGENIZATION PROCEDURE.……………………………….. 6

[1A. Development of two-step homogenization procedure 6](#_Toc4875673)

[1B. Outer mitochondrial membrane integrity 9](#_Toc4875674)

[1C. Optimization of homogenate concentration: comparison of 10%, 5%, 2.5% and 1% homogenate. 9](#_Toc4875675)

[FINAL STEP-BY-STEP PROTOCOL OF HUMAN HEART MUSCLE HOMOGENATES PREPARATION: 10](#_Toc4875677)

[SUIT protocol for high resolution respirometry 20](#_Toc4875691)

[2A. Titration of reagents 20](#_Toc4875692)

[2B. Final respirometry protocol 21](#_Toc4875696)

[2C. Influence of oligomycin concentration on FCCP-induced uncoupled maximal respiration 21](#_Toc4875697)

[2D. Possible modifications of protocol 21](#_Toc4875698)

[2E. The dependency of oxygen consumption rate on oxygen concentration. 24](#_Toc4875700)

[2F. Durability experiments 25](#_Toc4875701)

[ISOLATION OF MITOCHONDRIA 28](#_Toc4875702)

[COMPARISON OF CARDIAC MUSCLE HOMOGENATES WITH ISOLATED MITOCHONDRIA 30](#_Toc4875705)

[COMPARISON OF ATRIAL VS VENTRICULAR HOMOGENATES 31](#_Toc4875706)

3A. Completed data ………………………………………………………………………. 31

3B. Sex-based differences ………………………………………………………………... 32

[ELECTRON MICROSCOPY 32](#_Toc4875707)

[REFERENCES 33](#_Toc4875708)

# LIST OF ABBREVIATIONS:

AA Antimycin A

ADP Adenosine 5′-diphosphate monopotassium salt dihydrate

ATP Adenosine 5′-triphosphate disodium salt hydrate

BIOPS Biopsy preservation solution

BSA Bovine serum albumin

CABG Coronary artery bypass grafting

CAD Coronary artery disease

COPD Chronic obstructive pulmonary disease

CPR Cardiopulmonary resuscitation

CV Coefficient of variation

CS Citrate synthase

CYT C Cytochrome c

DMSO Dimethyl sulfoxide

EGTA Ethylene glycol-bis(2-aminoethylether)-N,N,N′,N′-tetraacetic acid

ETS Electron transfer system

Etom Etomoxir

FAO Fatty acid oxidation

FCCP Carbonyl cyanide 4-(trifluoromethoxy)phenylhydrazone

GLUT Glutamate

HEPES 4-(2-Hydroxyethyl)piperazine-1-ethanesulfonic acid, N-(2-Hydroxyethyl)piperazine-N′-(2-ethanesulfonic acid)

LMEM Linear mixed effect model

OLIGO Oligomycin

OXPHOS Oxidative phosphorylation

PC Palmitoyl-carnitine

MAL Malate

MES **2-(N-Morpholino)ethanesulfonic acid**

**MI Myocardial infarction**

MiR05 Mitochondrial respiration medium

PTFE Polytetrafluoroethylene

REB Research Ethics Board

Rote Rotenone

ROX Residual oxygen consumption

SUC Succinate

SUIT Substrate-uncoupler-inhibitor-titration

T1DM Diabetes mellitus, type 1

T2DM Diabetes mellitus, type 2

TMPD N,N,N′,N′-tetramethyl-p-phenylenediamine

# LIST OF CHEMICALS:

- Adenosine 5′-diphosphate monopotassium salt dihydrate (ADP), A5285-1G, Sigma-Aldrich (St. Louis, USA)
- Adenosine 5′-triphosphate disodium salt hydrate (ATP), A2383-1G, Sigma-Aldrich (St. Louis, USA)
- Antimycin A (Streptomyces sp.), A8674-25MG, Sigma-Aldrich (St. Louis, USA)
- Bovine serum albumin, lyophilized powder, A6003-25G, Sigma-Aldrich (St. Louis, USA)
- Carbonyl cyanide 4-(trifluoromethoxy)phenylhydrazone (FCCP), C2920-10MG, Sigma-Aldrich (St. Louis, USA)
- CelLytic™ MT Cell Lysis Reagent, C3228-500ML, Sigma-Aldrich (St. Louis, USA)
- Cytochrome c from equine heart, C7752-100MG, Sigma-Aldrich (St. Louis, USA)
- DL-Dithiothreitol, D0632-5G, Sigma-Aldrich (St. Louis, USA)
- Ethylene glycol-bis(2-aminoethylether)-N,N,N′,N′-tetraacetic acid (EGTA), E4378-25G, Sigma-Aldrich (St. Louis, USA)
- L-Glutamic acid monosodium salt hydrate, G1626-100G, Sigma-Aldrich (St. Louis, USA)
- HEPES Bioextra pH 5.0-6.5 (1M in H2O), H7523-250G, Sigma-Aldrich (St. Louis, USA)
- Imidazole, **56750-100G,** Sigma-Aldrich (St. Louis, USA)
- Lactobionic acid 97%, 153516-25G, Sigma-Aldrich (St. Louis, USA)
- Magnesium chloride hexahydrate (MgCl_2_ · 6H_2_O), M2393-500G, Sigma-Aldrich (St. Louis, USA)
- L-(−)-Malic acid, M1000-100G, Sigma-Aldrich (St. Louis, USA)
- D-Mannitol, M4125-500G, Sigma-Aldrich (St. Louis, USA)
- MES free acid hydrate, M8250-250G, Sigma-Aldrich (St. Louis, USA)
- Oligomycin, O4876-5MG, Sigma-Aldrich (St. Louis, USA)
- Phosphocreatine disodium salt hydrate, P7936-1G, Sigma-Aldrich (St. Louis, USA)
- Potassium hydroxide, P1767-500G-D, Sigma-Aldrich (St. Louis, USA)
- Potassium phosphate monobasic, P5655-500G, Sigma-Aldrich (St. Louis, USA)
- Protease inhibitor cocktail, P8340-5ML, Sigma-Aldrich (St. Louis, USA)
- Succinic acid, S9512-100G, Sigma-Aldrich (St. Louis, USA)
- D-(+)Sucrose, 84097-1KG, Sigma-Aldrich (St. Louis, USA)
- Taurine, T0625-25G, Sigma-Aldrich (St. Louis, USA)

**OPTIMIZATION OF HOMOGENIZATION PROCEDURE:**

## 1A. Development of two-step homogenization procedure

*Optimization of homogenate preparation, mitochondrial membrane integrity and reproducibility of the method.* Various homogenizers made from borosilicate glass or PTFE material (Micro Tissue Grinder Kit; Wheaton, Millville, USA) with different clearance between pestle and tube were used for homogenization. Homogenizer sets composed of glass tube and glass pestle were compared with those containing glass tube and PTFE pestle (see **Figure S1, parts A** and **B**). Different surfaces of homogenizers also were tried, e.g. roughened & smoothened glass or PTFE pestle covered by completely smoothened material & serrated surface with small notches on the edge of the bottom (see **Figure S2, parts A** and **B**). Homogenization process was performed on motor-driven homogenizer (HEi-Torque Value 100, Heidolph, Germany), where we tested various number of strokes and speed.

The 2mL Potter-Elvehjam homogenizer with tight clearance (0.11-0.15 mm, Wheaton^TM^, Millville, USA, material of tube: glass; material of pestle: glass) was excluded because of outer mitochondrial membrane damage when oxygen consumption after addition of 10 µM cytochrome c increased over 40% (see **Figure 1, part A** and **Figure S6**).

Homogenization with 2 mL glass tube and serrated PTFE pestle with small notches on the bottom edge (with clearance 0.15-0.25 mm; Wheaton^TM^, Millville, USA, see **Figure S2, part A**) showed a lesser damage of mitochondrial membrane (increase after cyt c 16±6.6%) although substantial heterogeneity between two homogenates was evident (CV<34% for all measurements) and inner mitochondrial membrane suffered from moderate uncoupling (45±20%).

The variability was reduced when the serrated PTFE pestle was replaced by a PTFE pestle with a smooth surface (Wheaton 1 mL Tapered Tissue Grinder Set with PTFE Pestle, Wheaton^TM^, Millville, USA, see **Figure S3**). In addition, we added another step involving filtration of crude homogenate through polyamide mesh which helped us to further decrease variability between two homogenates (CV<20% for all absolute measurements and dimensionless derived indices). On the other hand, increase of oxygen consumption after addition of cytochrome c was again high (increase after cyt c 37±11%) and inner mitochondrial membrane was significantly uncoupled (55±7%). Therefore, we continued with further experiments using other homogenizer sets.

The best results were obtained by two-step homogenization process: in the first part of homogenization, minced tissue fragments were diluted in MiR05 buffer to obtain a 10% homogenate (10 mg of tissue/100 ml of medium) and manually homogenized by 10-12 strokes up and down using a Dounce tissue grinder set (large clearance 0.114 ± 0.025 mm; Wheaton^TM^, Millville, USA; see **Figure S4**). The next step was performed in the same glass tube of previously used Dounce grinder (see **Figure S5**) with 5-6 slow strokes by motor-driven (750 rpmi; HEi-Torque Value 100, Heidolph, Germany) PTFE pestle of 2mL Potter-Elvehjem homogenizer (Micro Tissue Grinder Kit; Wheaton^TM^, Millville, USA) placed Crude homogenate was then filtered through polyamide technical screen (parameters: loop size 335 µm, fibre diameter 120 µm, 100% polyamide; SILK & PROGRESS s.r.o., Czech Republic; see **Figure 13**). This method led to a preserved outer mitochondrial membrane (increase of oxygen consumption after cyt c was 15±2%), mild uncoupling of inner mitochondrial membrane (33±3%) and very good reproducibility of the results (CV ˂10% for all absolute measurements and ˂3% for dimensionless derived indices).


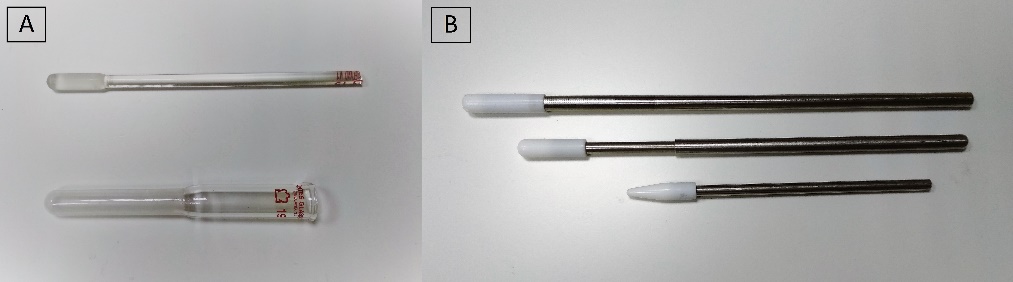


**Figure S1.** **A)** Potter-Elvehjem homogenizers with glass tube and pestle; **B)** Potter-Elvehjem homogenizers with PTFE pestle

from top to bottom: *rounded* shape on the lower portion of the pestle with *serrated* surface on the bottom edge; *rounded* shape on the lower portion of the pestle with completely *smoothened* surface; *tapered* shape on the lower portion of the pestle with completely *smoothened* surface).


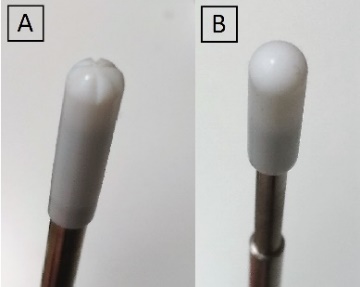


**Figure S2.** Detail of PTFE pestle. **A)** PTFE pestle of *rounded* shape on the lower portion with *serrated* surface (notches) on the bottom edge; **B)** PTFE pestle of *rounded* shape on the lower portion with completely *smoothened* surface (without notches).


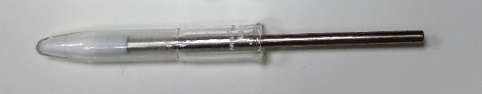


**Figure S3.** 1 mL Tapered Tissue Grinder Set (Potter-Elvehjem homogenizer) with PTFE Pestle of *tapered* shape on the lower portion with completely *smoothened* surface.


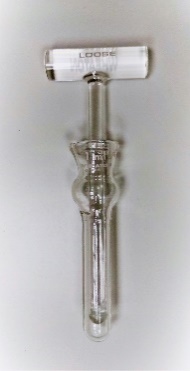


**Figure S4.** Homogenizer set that was used for the *first step of homogenization procedure* *(manual)*. Wheaton^TM^ 1mL Dounce Tissue Grinder (Wheaton^TM^, Millville, USA); Glass tube and glass (Loose) pestle.


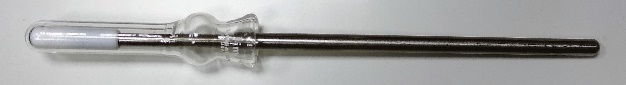


**Figure S5.** The combination of homogenizer parts that were used for the *second step of homogenization procedure* *(motor-driven)*. Potter-Elvehjem with PTFE Pestle of *ground* shape on the lower portion with completely *smoothened* surface (Wheaton^TM^, Millville, USA) placed in 1 mL glass tube of Dounce Tissue Grinder Set (Wheaton^TM^, Millville, USA).

## 1B. Outer mitochondrial membrane integrity


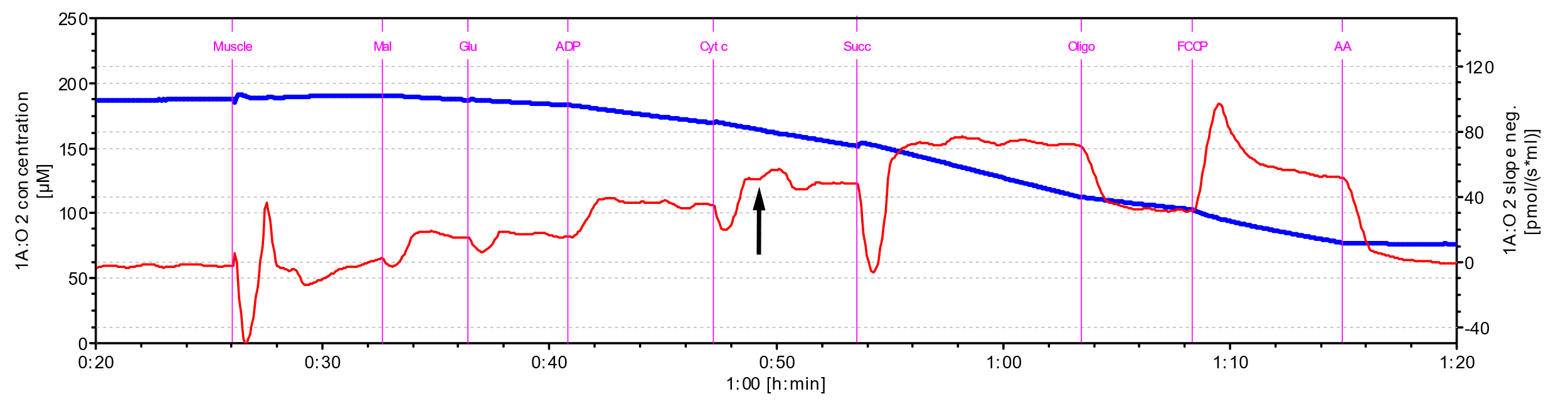


**Figure S6.** Glass-glass homogenizer, Potter-Elvehjem homogenizer with glass tube and pestle (Wheaton^TM^, Millville, USA), clearance 0.11-0.15 mm, 7-8 strokes, speed 750 rpm. Oxygen consumption after addition of 10 µM cytochrome c increased over 20%.


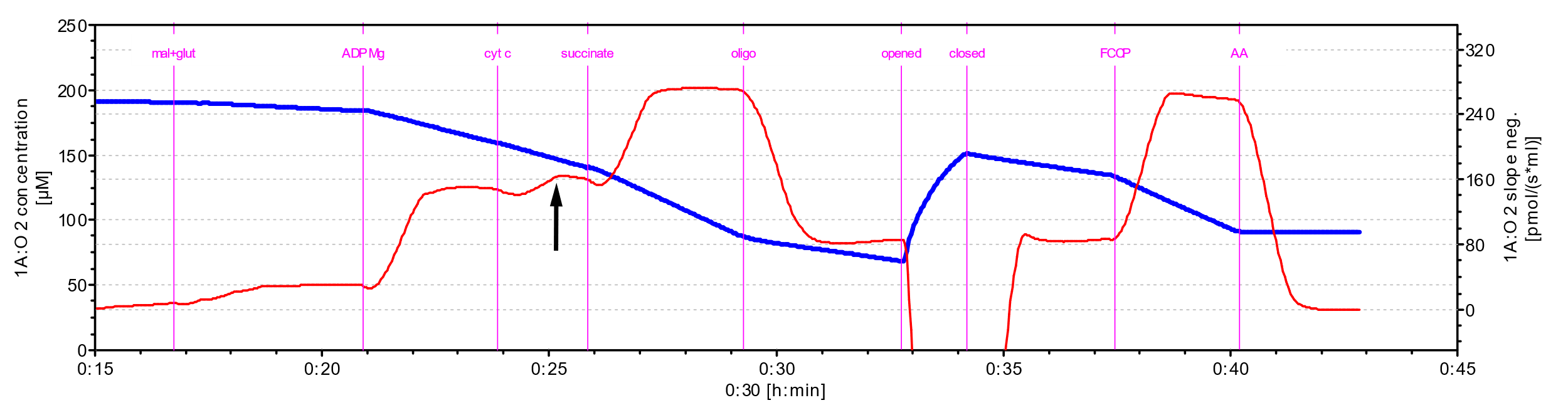


**Figure S7.** Teflon-glass homogenizer (Wheaton Round Tissue Grinder Set [Potter-Elvehjem homogenizer] with PTFE Pestle of *round* shape on the lower portion with completely *smoothened* surface (Wheaton^TM^, Millville, USA), 5-6 strokes, speed 750 rpmi). Oxygen consumption after addition of 10µM cytochrome c was up to 15% of baseline.

## 1C. Optimization of homogenate concentration: comparison of 10%, 5%, 2.5% and 1% homogenate.

**
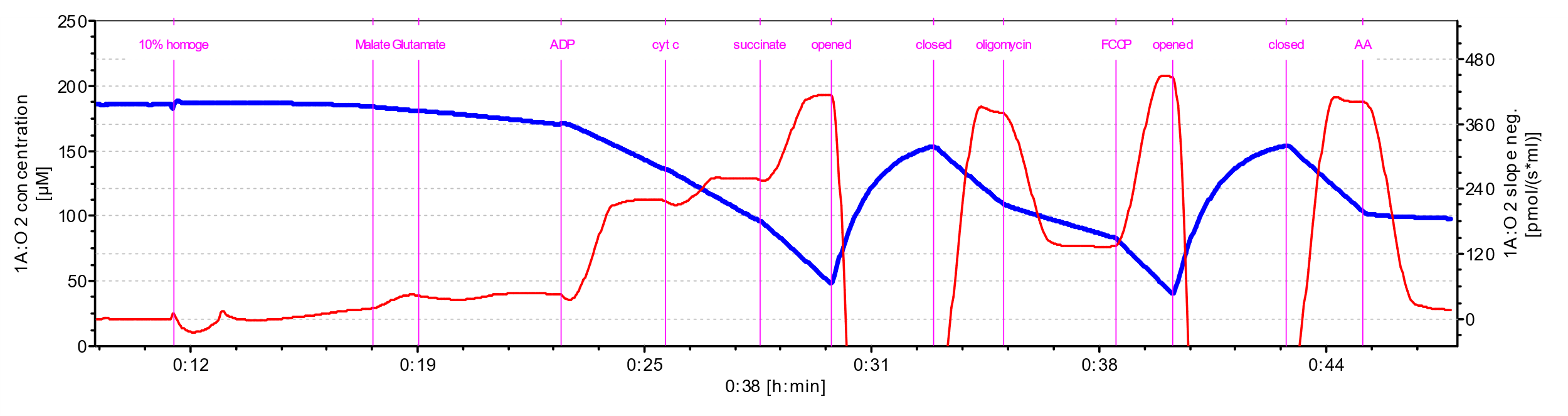
**

**Figure S8.** 10% homogenate. Rapid exhaustion of oxygen levels in 30 minutes.


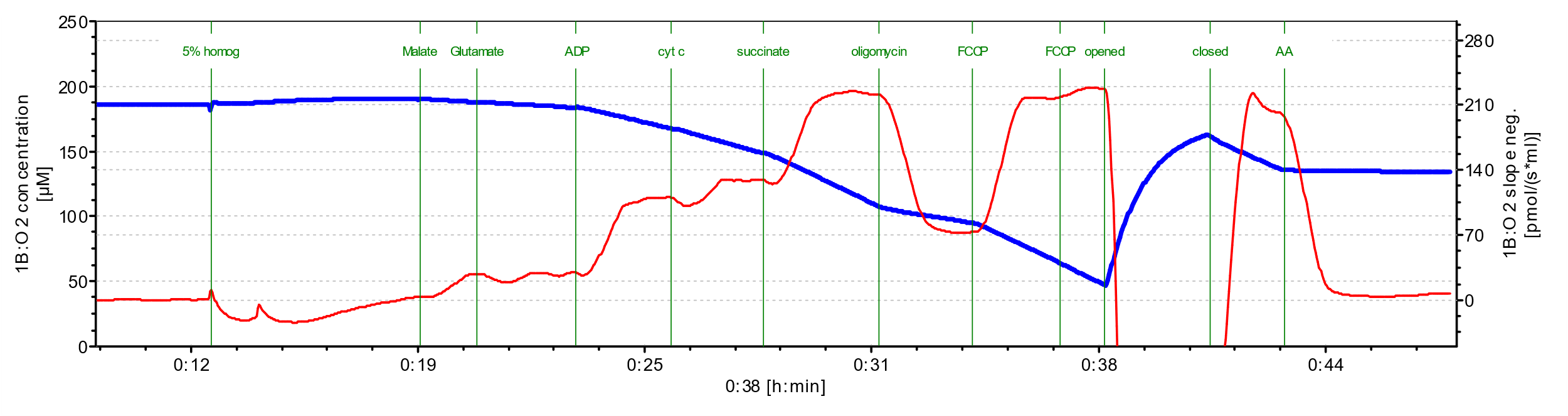


**Figure S9.** 5% homogenate. Rapid exhaustion of oxygen levels in 30 minutes.


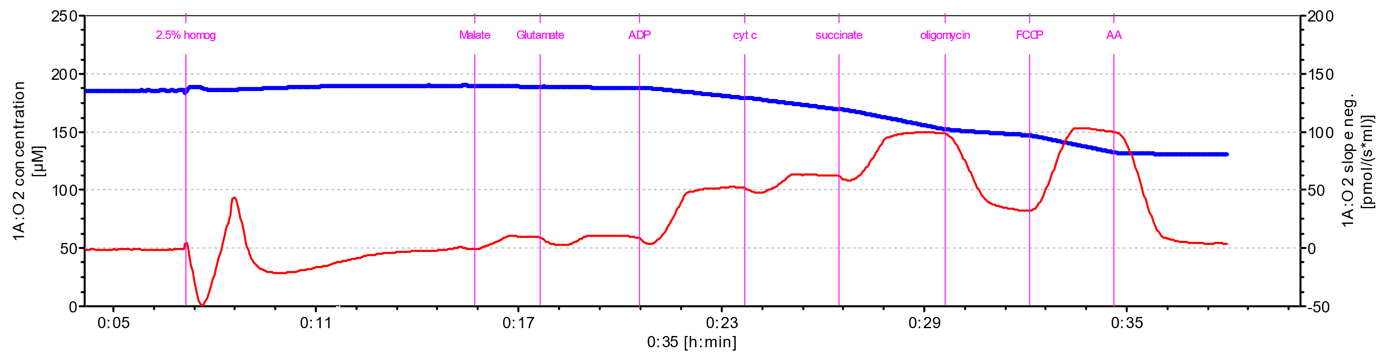


**Figure S10.** 2.5% homogenate. Optimal concentration of homogenate (whole experiment is processed without a need of opening the chamber).


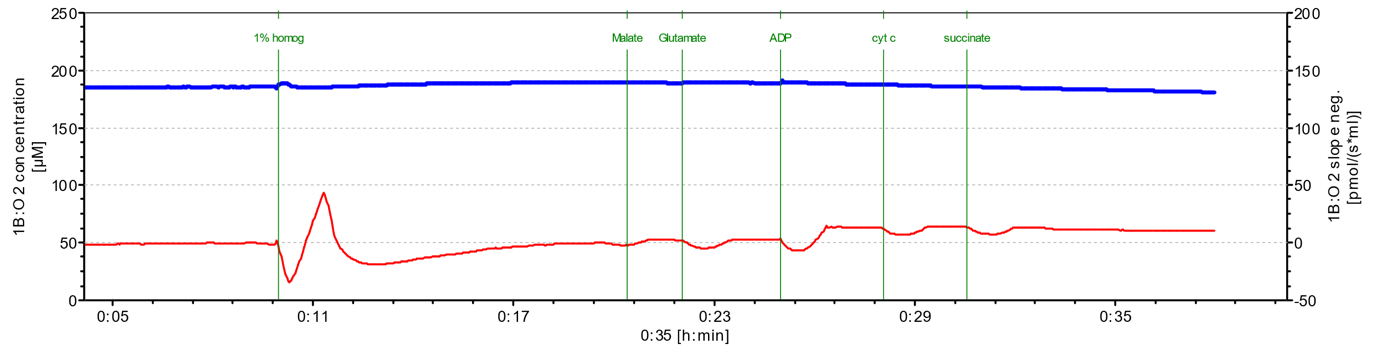


**Figure S11.** 1% homogenate. Almost no icrease of oxygen consumption after addition of substrates and ADP.

## **FINAL STEP-BY-STEP PROTOCOL OF HUMAN HEART MUSCLE HOMOGENATES PREPARATION:**

Homogenates were prepared with partial modifications according to the protocol for rat heart tissue homogenates[1] and the protocol for human skeletal muscle homogenates, previously developed by our research group[2].

**All steps of procedure must be strictly performed on ice.**

1. Wash properly all tubes, scissors, forceps, teflon pestles and glassware (including Dounce Tissue homogenizer and glass pestles) with 70% ethanol and tap water and keep them on ice to cool to 0°C. Immediatelly before use, rinse the instruments once again using distilled water and thoroughly dry them with clean gauze. Specifications of each instrument needed for homogenization are decribed in **Figure S12**.


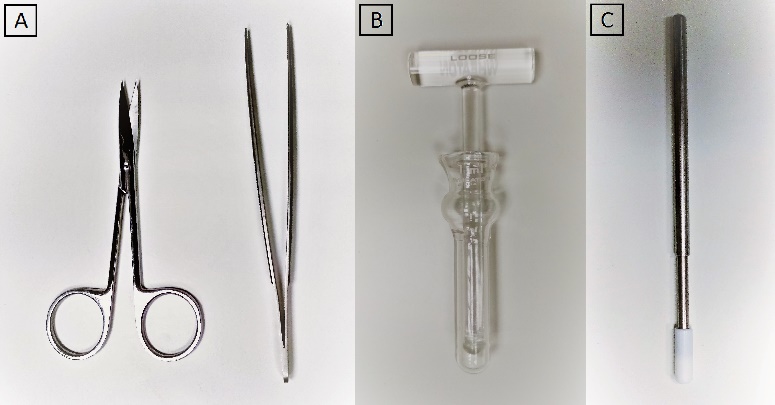


**Figure S12.** **A)** Standard Laboratory scissors and Anatomic straight forceps; **B)** Wheaton^TM^ 1mL Tissue Grinder, Dounce (Wheaton^TM^, Millville, USA); Loose pestle (larger clearance 0.114 ± 0.025 mm); material of tube and pestle: borosilicate glass; **C)** Shaft with Pestle of Wheaton^TM^ 2mL Potter-Elvehjem Tissue Grinder, (Wheaton^TM^, Millville, USA); diameter shaft: 6.3 mm; Pestle material: PTFE.

1. Prepare stitched cornet from polyamide mesh (see **Figure S13, parts A** and **B**).


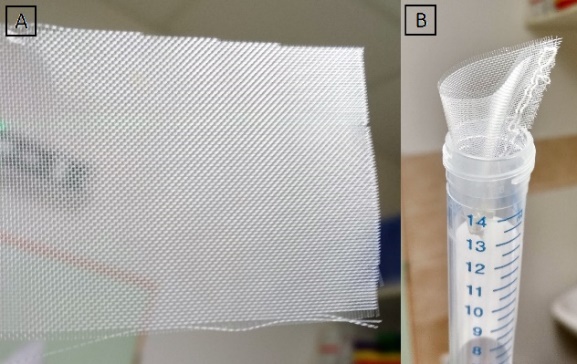


**Figure S13.** **A)** polyamide technical screen (parameters: loop size 335 µm, open space 54 %, fibre diameter 120 µm, material: 100% polyamide; SILK & PROGRESS s.r.o., Czech Republic); **B)** Filtration cornet, sewn from polyamide mesh.

1. Immediately after dissection, insert muscle biopsy sample from heart appendage/ventricle into plastic 50 ml Falcon tube with 10 ml of cold BIOPS and transfer it to the lab on ice in the closed plastic storage box.
2. Place the biopsy sample on a piece of parafilm on ice (see **Figure S14**).


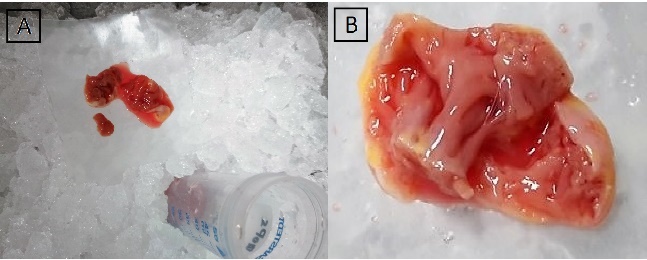


**Figure S14**. **A)** Muscle biopsy from human right appendage taken from BIOPS and placed on parafilm on ice; **B)** Detail of muscle biopsy specimen.

1. Remove fat, connective tissue and blood vessels from heart muscle tissue using clean pre-cold scissors and forceps (see **Figure S15**).


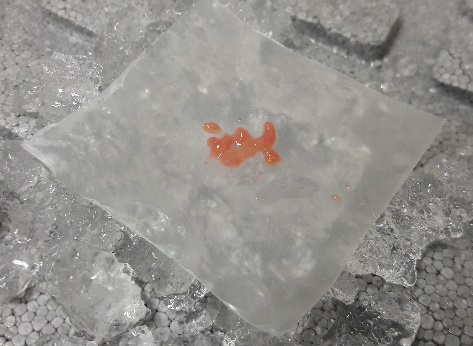


**Figure S15.** Heart mucle biopsy after removal of connective and adipose tissue.

1. Check the sample under the microscope. Place the sample on the piece of parafilm/or lid of Petri dish on the bottom plate of Petri dish (60 mm; polystyrene) filled with ice and put it under the microscope (see **Figure S16**). Check the sample and remove remaining fat and connective tissue if necessary.


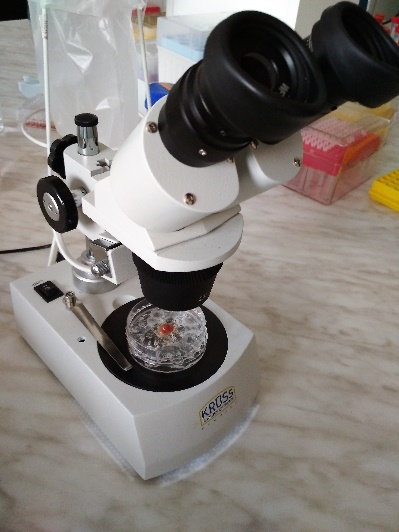


**Figure S16.** Heart mucle biopsy placed on the Petri dish under microscope.

1. Dry the sample gently by piece of gauze to remove residual BIOPS and blood (  ̴̴ 5 seconds), (see **Figures S17** and **S18**).


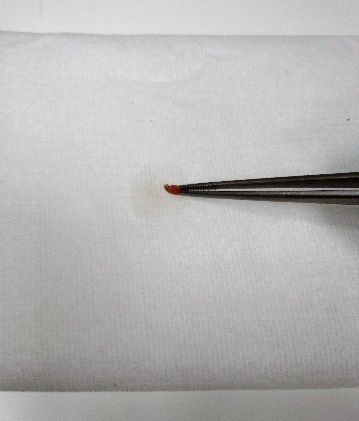


**Figure S17.** Heart muscle sample placed on the piece of gauze.


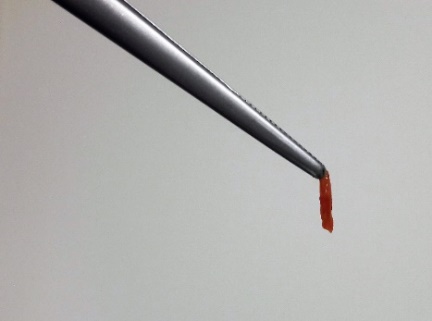


**Figure S18.** Heart muscle sample after extraction of the rest of BIOPS and blood.

1. Weight the sample on the analytical scale placed on parafilm (see **Figure S19**).


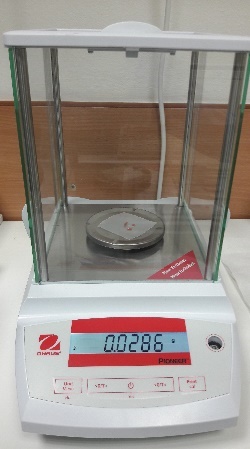


**Figure S19.** Weighing of muscle sample on the analytical scale.

1. After that, dissect the sample with scissors into fine fragments (see **Figure S20**). During this step, the muscle has to be placed on the parafilm on ice.


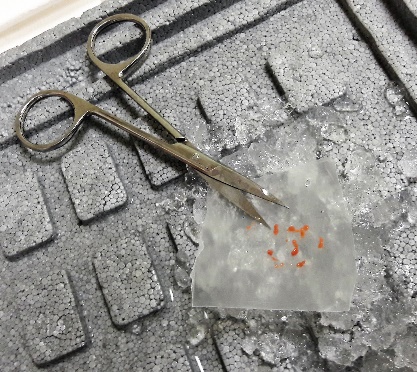


**Figure S20.** Fine fragments of heart mucle tissue cutted by scissors.

1. Transfer carefully the pieces of muscle into the pre-cold Wheaton^TM^ 1 mL Dounce Tissue Grinder and dilute in ratio: 100 mg of the muscle per 1 mL of MiR05 medium to obtain 10% muscle homogenate (see **Figure 21**).


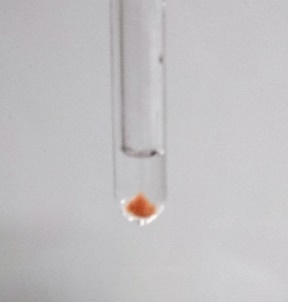


**Figure S21.** Fine fragments of heart mucle tissue, diluted in MiR05, placed in Dounce Tissue Grinder.

1. Homogenize gently in 1 mL Dounce Tissue Grinder by moving the pre-chilled glass pestle (Loose) up and down (10-12 strokes) (see **Figures S22** and S**23**). During the procedure, Grinder has to be placed on ice.


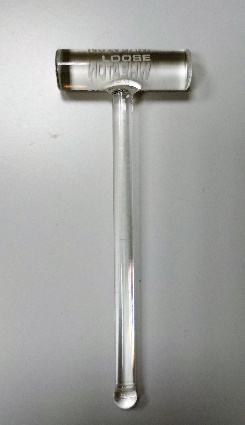


**Figure S22.** Glass pestle (Loose) from Dounce Tissue Grinder Set.


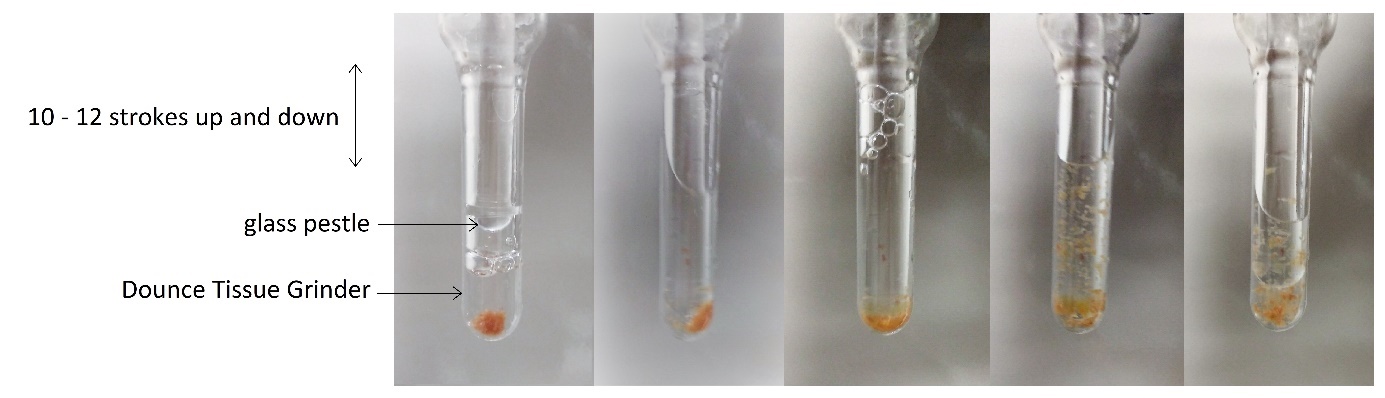


**Figure S23.** Manual homogenization with 10 – 12 up and down. The process must be done **on ice**.

1. Wash the shaft of the PTFE pestle from Wheaton^TM^ 2mL Potter-Elvehjem Tissue Grinder with distilled water and dry the pestle by sterile gauze. Fix the shaft into motor-driven homogenizer (HEi-Torque Value 100, Heidolph, Germany). Set the speed to 750 rpmi (see **Figure S24**).


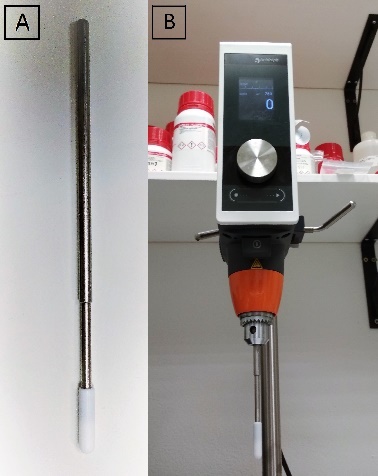


**Figure S24.** **A)** Shaft with PTFE pestle from Wheaton^TM^ 2mL Potter-Elvehjem Tissue; **B)** Motor-driven homogenizer (HEi-Torque Value 100, Heidolph, Germany) with fixed shaft with PTFE pestle; speed set at 0 rpmi (still not running).

1. Insert the glass tube of 1 mL Dounce Tissue Grinder (with muscle pieces floating in MiR05) into plastic 50 mL Falcon tube with crushed ice (diluted with small amount of tap water). Be careful to leave the upper part of glass tube protruding out of the crushed ice. Immerse the PTFE pestle into the glass tube of 1 mL Dounce Tissue Grinder and turn on the motor-driven homogenizer. Whilst speed is going from 0 to 750 rpmi, slowly move the pestle down to touch the bottom of glass tube. Gently move up and down 5-6 times whilst the pestle is still inserted in the homogenate (this helps to avoid creating bubbles inside under pressure - see **Figure S25**).


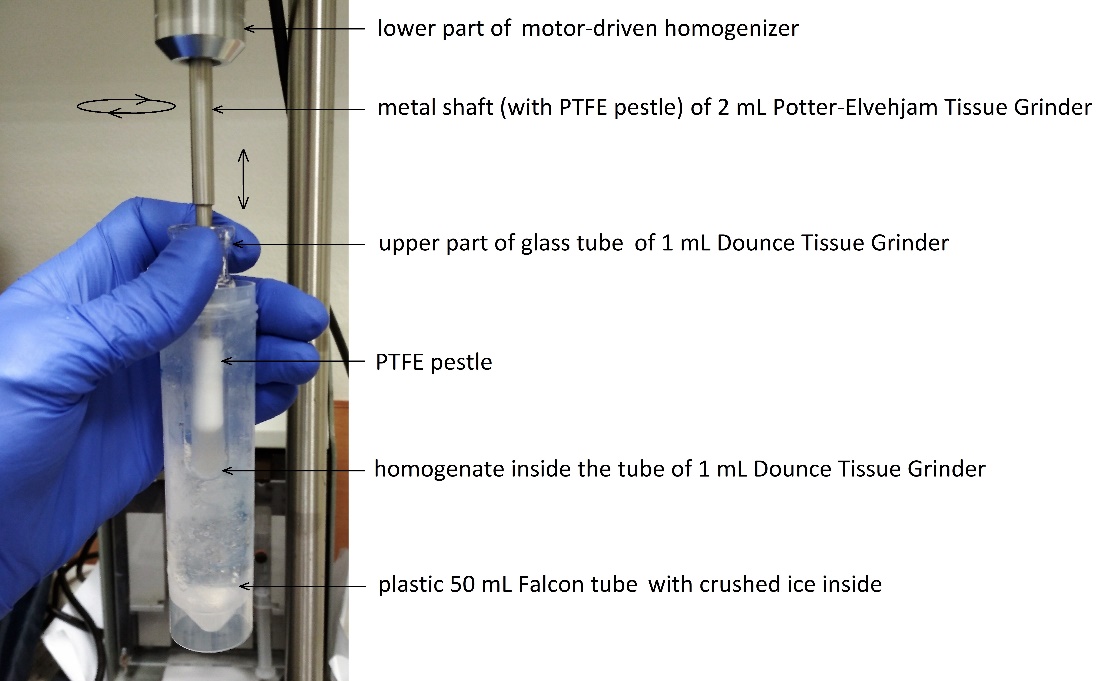


**Figure S25.** Motor-driven homogenization (750 rpmi) simultaneously with manual 5-6 strokes up and down.

1. At the end of the homogenization process, slowly pull down the glass tube (of 1 mL Dounce Tissue Grinder) in tube with crushed ice from the pestle and simultaneously gradually slow down the speed of motor-driven homogenizer by turning the wheel (back to 0 rpmi).
2. Dilute the 10 % homogenate with MiR05 es to obtain a final concentration of 2.5%.
3. Repeat step 11: motor-driven homogenization (750 rpmi) with simultaneous manual 3-4 (only!) strokes up and down. You will get crude 2.5% homogenate (see **Figure 26**).


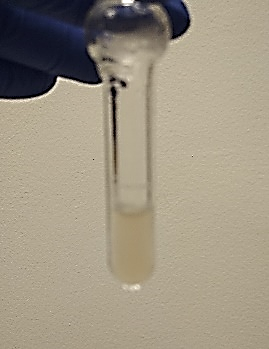


**Figure S26.** 2.5 % crude homogenate after multi-step homogenization.

1. Filter crude homogenate through polyamide technical screen into pre-cold plastic 12 mL Falcon tube or 2 mL Eppendorf tube (see **Figure 27**).


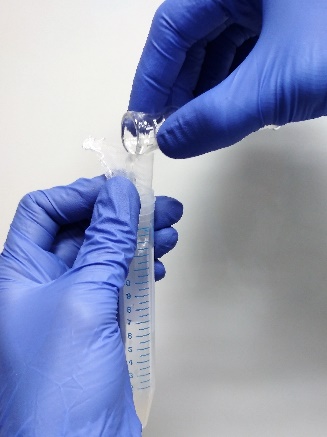


**Figure S27.** Filtration of crude homogenate through polyamide mesh.

1. Store the Falcon tube or Eppendorf tube with filtered homogenate on ice until the polarographic experiment will be performed (**do not leave the homogenate on ice more than 1 hour**; in case that measurement is not performed until 1 hour after homogenization **it is necessary** to prepare a new fresh homogenate).
2. After calibration of Oxygraph-2k, add 200 µL of filtered homogenate to the 1.8 ml MiR05 in the chamber (use pipette with tips of 1 mL volume) (see **Figure S28**).


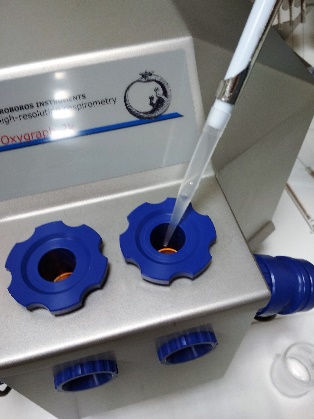


**Figure S28.** Pipetting of filtered homogenate into the opened Oxygraph-2k chamber.

1. Inject the agents (substrates, uncouplers or inhibitors) according to SUIT protocol into the chamber with Hamilton syringes (see **Figure S29**).


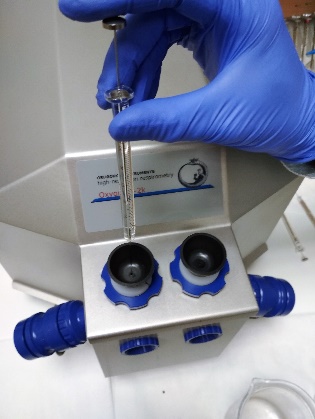


**Figure S29.** Manual pipetting of agents into the closed Oxygraph-2k chamber (via a small capillary tube in a black lid) with Hamilton syringe.

# SUIT protocol for high resolution respirometry

## 2A. Titration of reagents

##


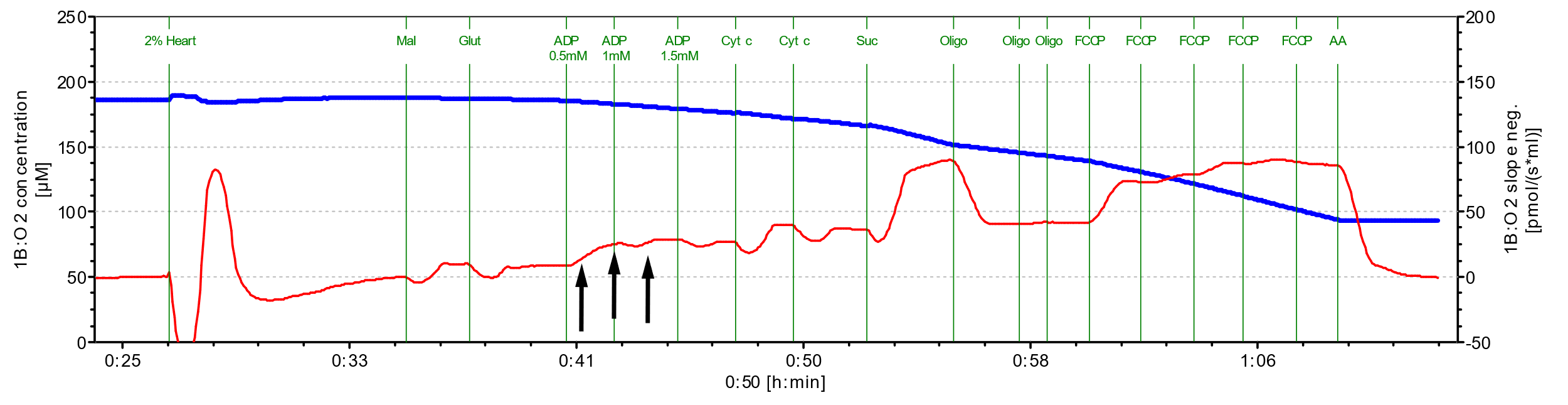


**Figure S30.** Titration of substrates. ADP at concentrations 0.5, 1 and 1.5 mM.


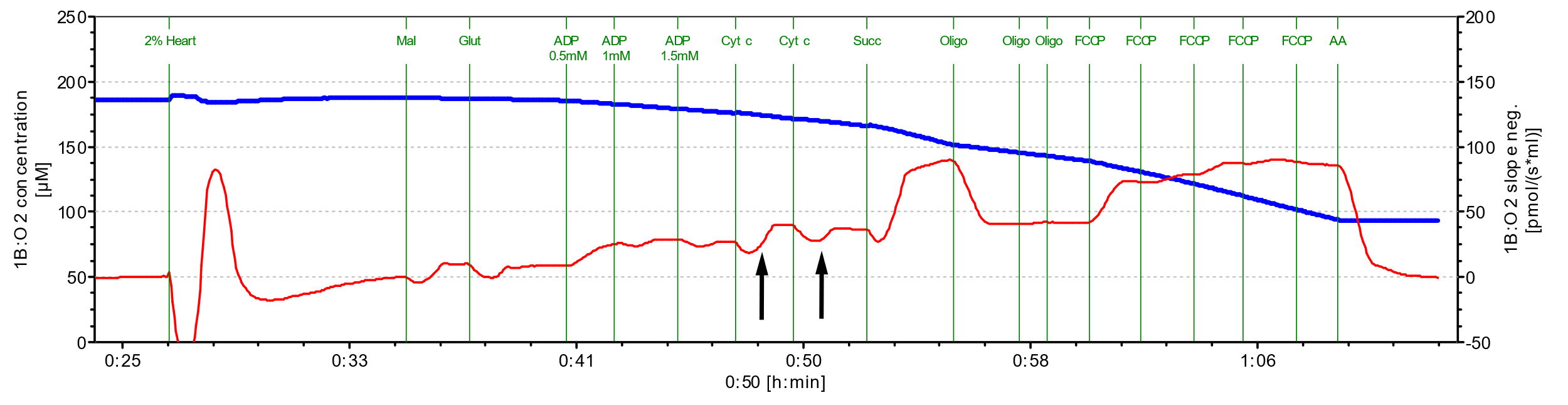


**Figure S31.** Titration of substrates. Cytochrome c at concentrations 10 and 20 µM.


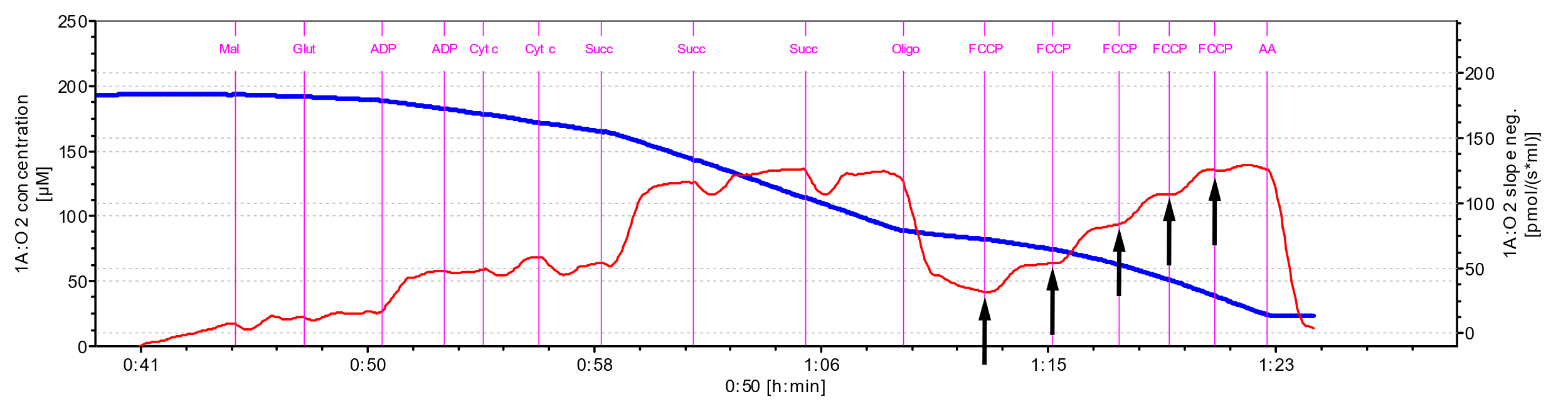


**Figure S32.** Titration of uncouplers. Titration of 0.25 µM steps to final concentration of 1.5 µM FCCP concetration.


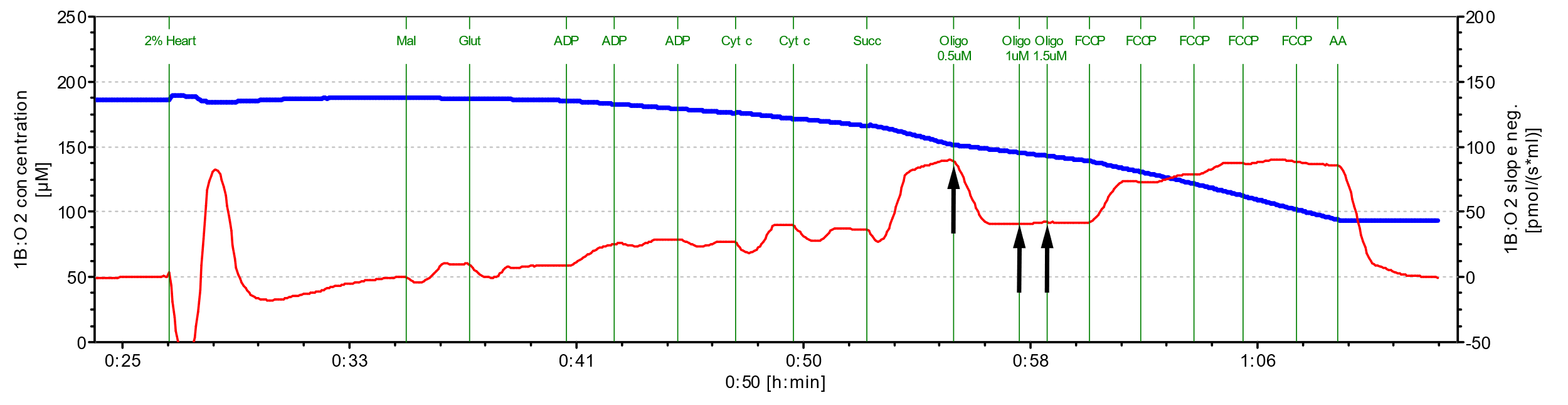


**Figure S33.** Titration of inhibitors. Oligomycin at concentrations 0.5, 1 and 1.5 µM.

## 2B. Final respirometry protocol

Respirometry protocol for assessment of standard bioenergetic parameters was obtained by sequential addition of 2.5 mM malate and 15 mM glutamate, 2.5 mM ADP, 10 µM cytochrome c, 10 mM succinate, 2.5 µM oligomycin, 1.5 µM FCCP, 3.5 µM rotenone, 4 µM antimycin A and 1 mM KCN.

We used higher concentration of ADP (2.5 mM) to avoid its rapid burst during measurement. ADP stock solution was prepared with 300 mM free Mg^2+^.

## 2C. Influence of oligomycin concentration on FCCP-induced uncoupled maximal respiration


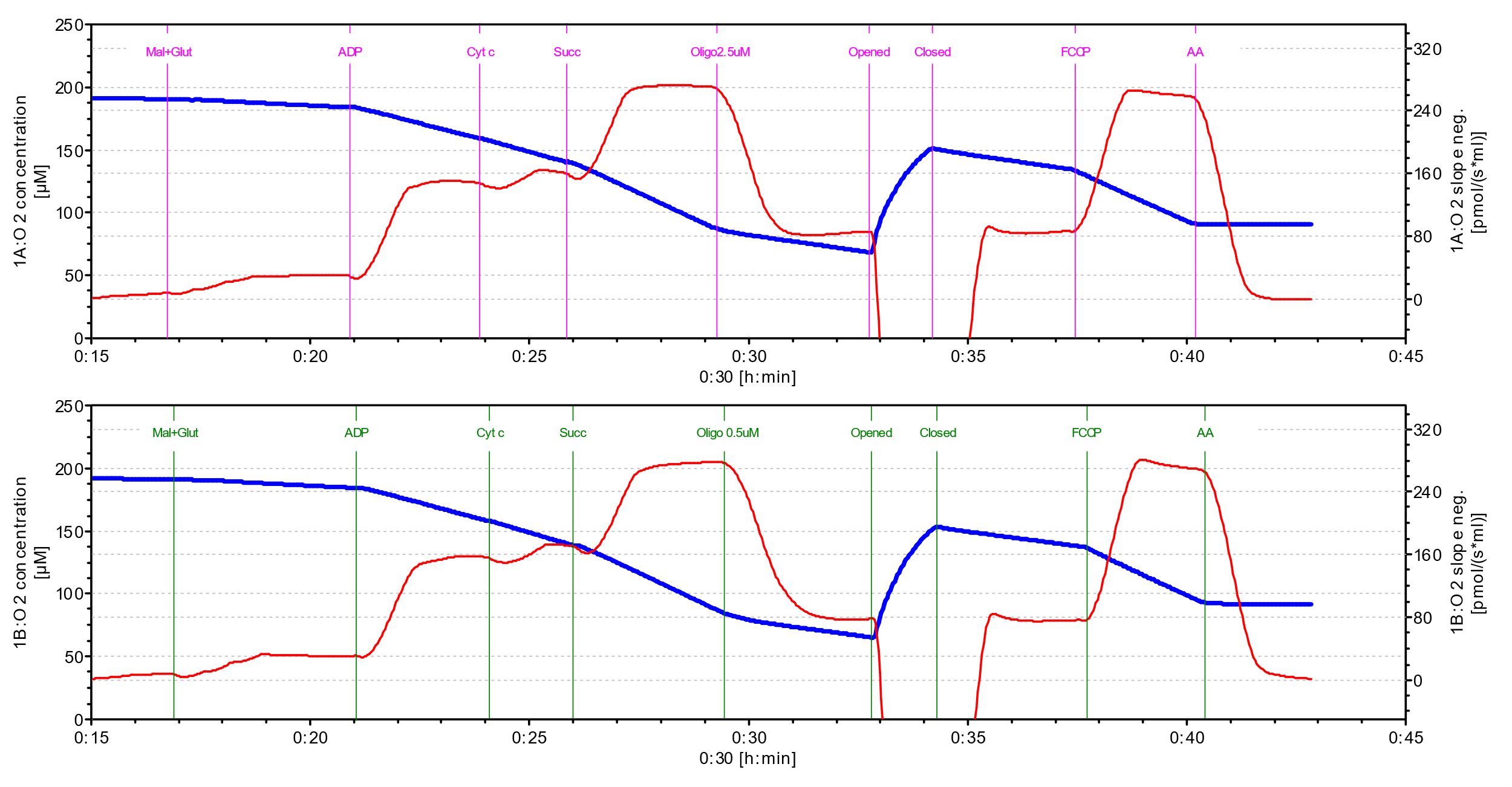


**Figure S34.** Comparison of impact of 2.5 µM and 0.5 µM oligomycin on FCCP-induced uncoupled maximal respiration. The experiment was repeated and showed no difference between these two concentrations (n = 3).

## 2D. Possible modifications of protocol

It is possible to modify the protocol by addition of different substrates and inhibitors. In the presence of abundant substrates and ADP, high resolution respirometry enables the measurement of both activity of the individual complexes of respiratory chain and capacity of fatty acid oxidation (FAO).

**Variations of respiratory protocol**

1. Additional determination of Complex II linked uncoupled respiration is possible to obtain by addition of inhibitor for Complex II = malonate (from difference: OCR after addition of substrates for complexes I and II, ADP, cyt c, oligomycin and FCCP minus OCR after the injection of above mentioned agents + *malonate*).

Sequential addition of agents: 2.5 mM malate, 15 mM glutamate, 2.5 mM ADP, 10 µM cytochrome c, 10 mM succinate, 2.5 µM oligomycin, 1.5 µM FCCP, 5 mM malonate, 4 µM Antimycin A.

1. Additional determination of Complex IV linked respiration is possible to obtain by addition of substrates and inhibitors in prolonged experiment:

Sequential addition of agents: 2.5 mM malate, 15 mM glutamate, 2.5 mM ADP, 10 µM cytochrome c, 10 mM succinate, 2.5 µM oligomycin, 1.5 µM FCCP, 4 µM Antimycin A, 10 mM Ascorbate, 0.2 mM TMPD, 100 mM Sodium Azide.

1. A protocol testing pyruvate dehydrogenase activity could be measured by sequential addition of: 2.5 mM malate, 5 mM pyruvate, 2.5 mM ADP, 10 µM cytochrome c, 10 mM succinate, 2.5 µM oligomycin, 1.5 µM FCCP and 3.5 µM rotenone, 4 µM Antimycin A and 1 mM KCN or 100 mM Sodium Azide (see **Figure S35**).


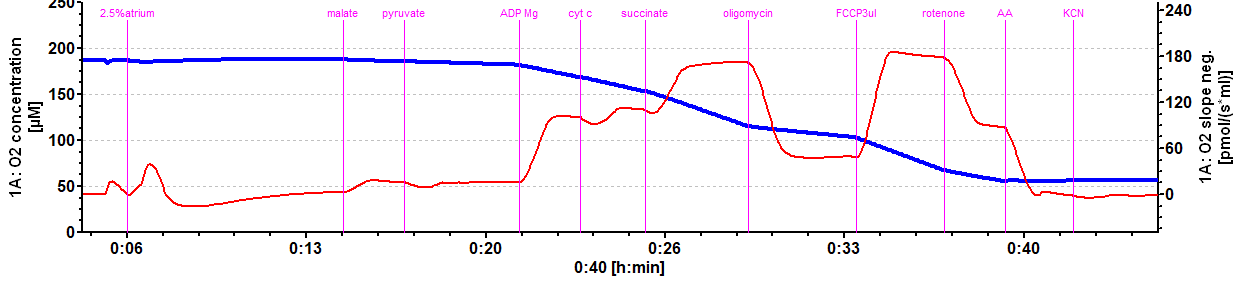


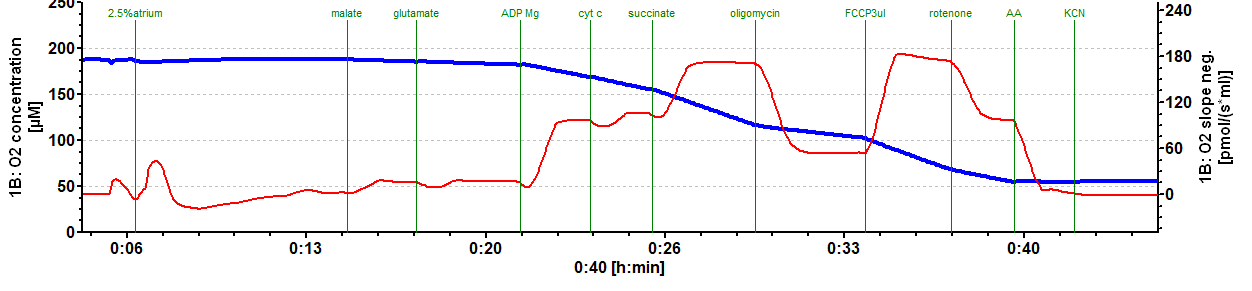


**Figure S35.** Upper part demonstrates protocol which uses combination of malate + pyruvate as a substrate combination for complex I, lower part showes protocol where malate + glutamate were used.

1. Determination of fatty acid oxidation capacity (see **Figure S36**) includes completely different protocol using a long-chain fatty acid as a substrate (=palmitoyl-carnitine) and rotenone as an inihibitor of FAO:

Sequential addition of agents: 2.5 mM malate, 2.5 mM ADP, 20 µM palmitoyl-carnitine (heated to 70°C before injection), 3.5 µM rotenone.


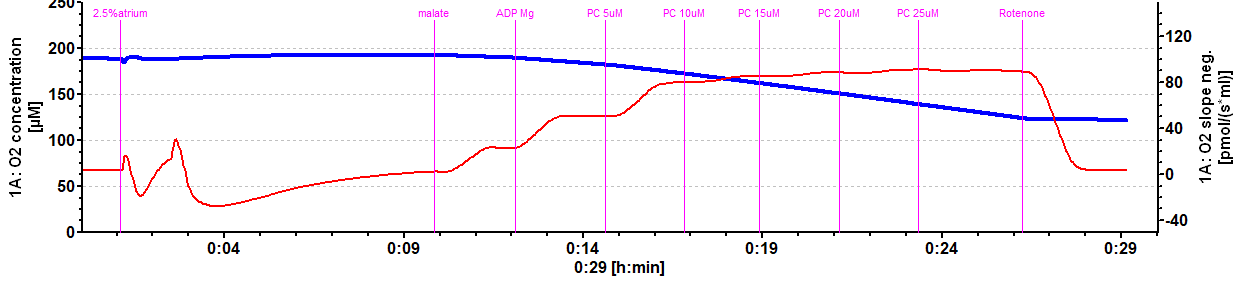


**Figure S36.** Fatty acid oxidation. The protocol demonstrates titration of palmitoyl-carnitine concentrations. The protocol was tested n = 4.

## 2E. The dependency of oxygen consumption rate on oxygen concentration.


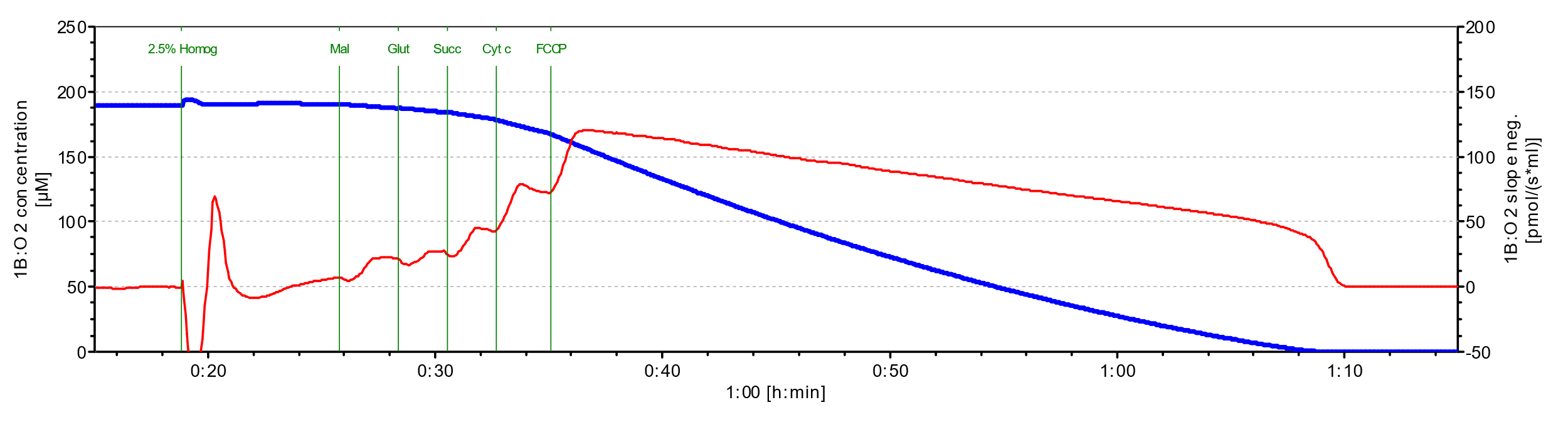

**Figure S37.** Upper: The dependency of oxygen flux on oxygen concentration after addition of substrates for complex I and II, cytochrome c and uncoupler FCCP. Lower: Relationship between oxygen concentration and consumption in the presence of abundant substrates and fully uncoupled mitochondria in human heart homogenate (right atrial appendage). Different colors represent different hearts (n = 7).

## 2F. Durability experiments

1. **Durability of muscle samples**


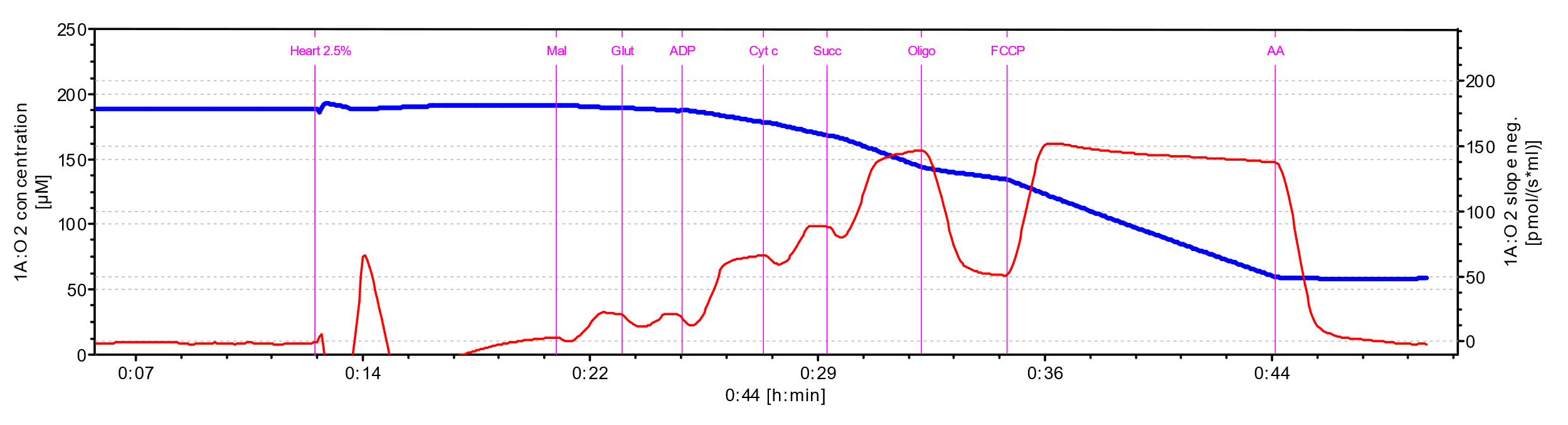


**
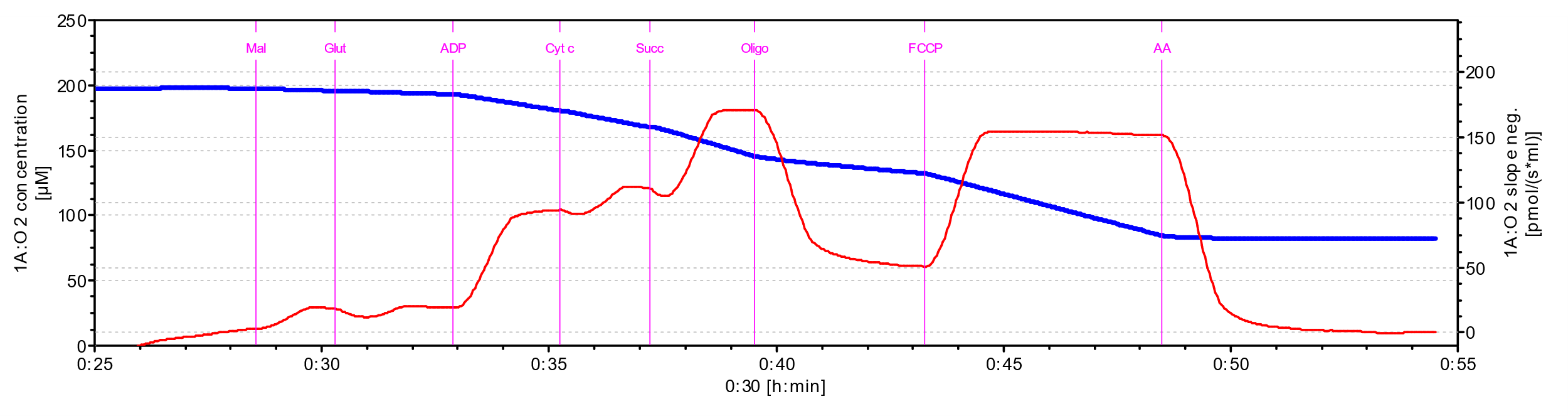
**

**
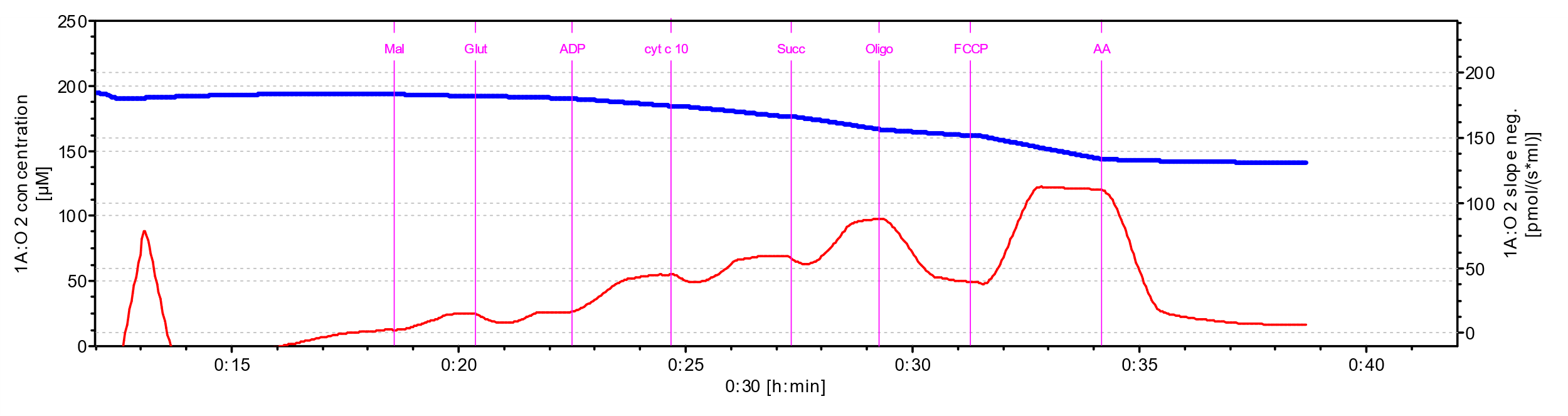
**

**
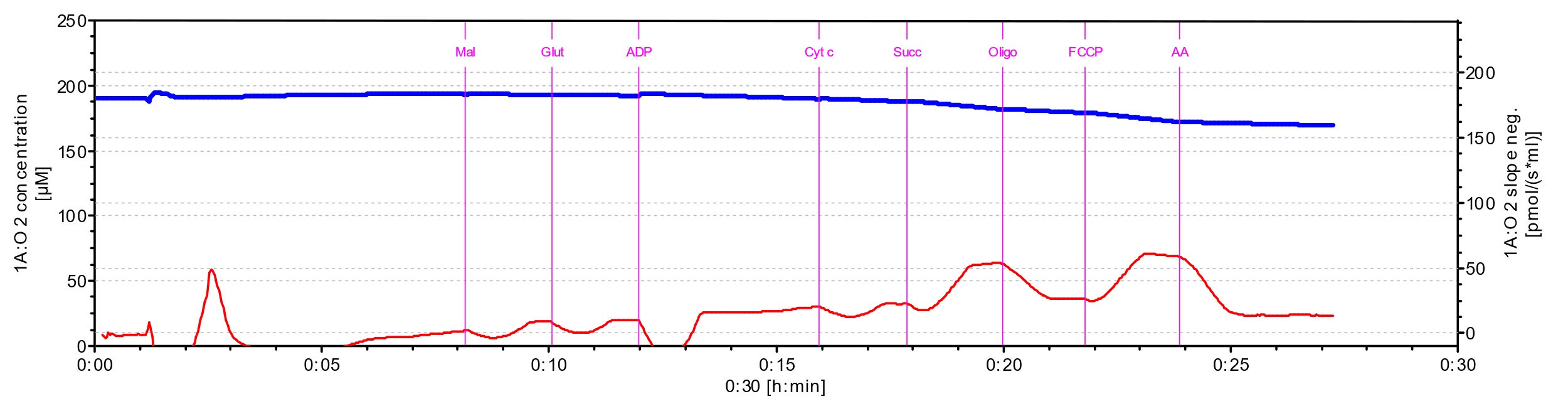
**

**Figures S38, S39, S40 and S41 (from top to bottom).** Durability of muscle samples. Comparison of measurement immediately after biopsy removal with measurements performed 24, 48 or 72 hours after biopsy (the biopsy was stored in BIOPS on ice and fresh homogenate was prepared directly before each measurement).

1. **Durability of tissue homogenates on ice**


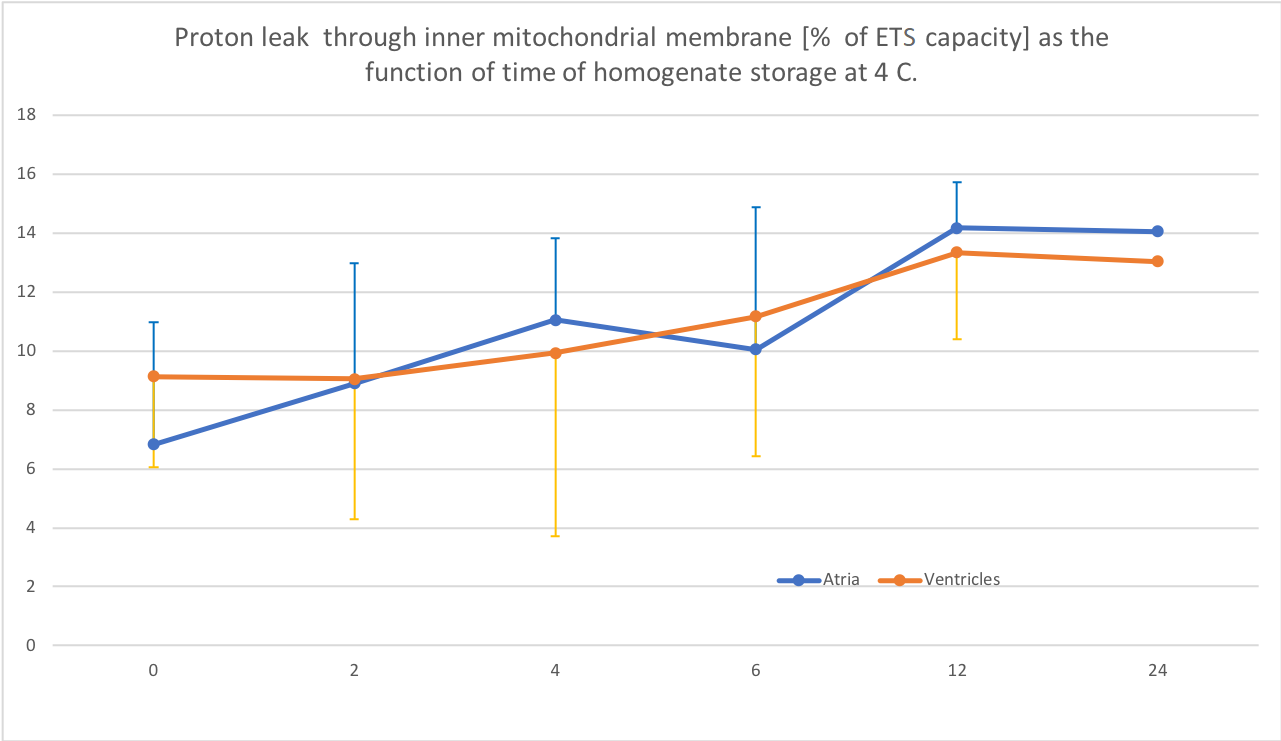


**Figure S42.** Durability of homogenates. Proton leak through inner mitochondrial membrane (% of ETS capacity) as the function of homogenate storage on ice.


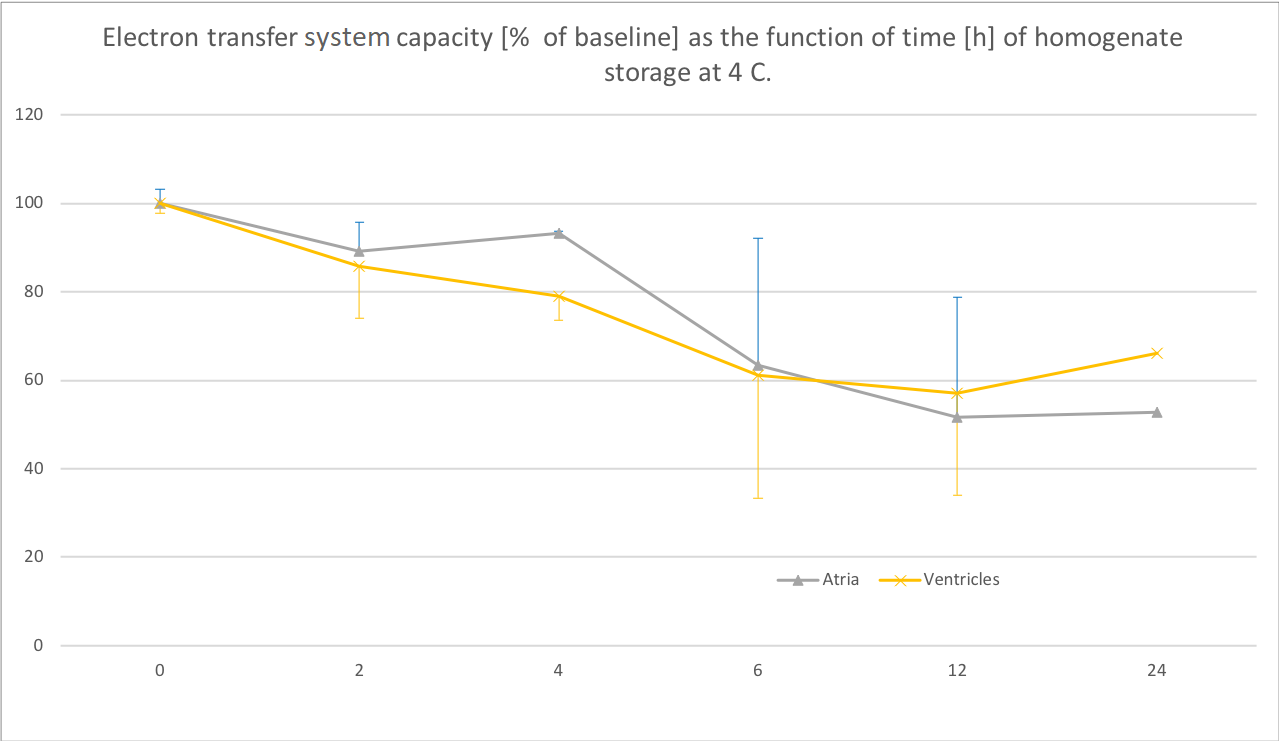


**Figure S43.** Durability of tissue homogenates. Electron transfer system capacity (% of baseline) as the function of time of homogenate storage on ice.

1. **Cryopreservation of tissue homogenates and native muscle**


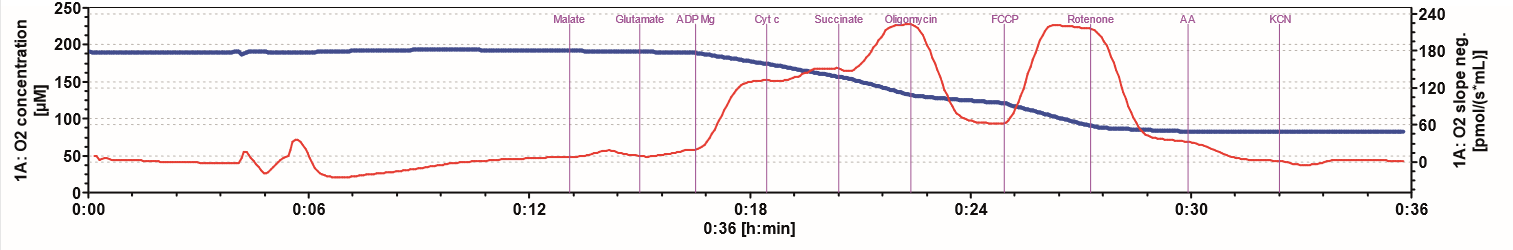


**Figure S44.** Homogenate prepared from fresh biopsy and measured immediately after homogenization.


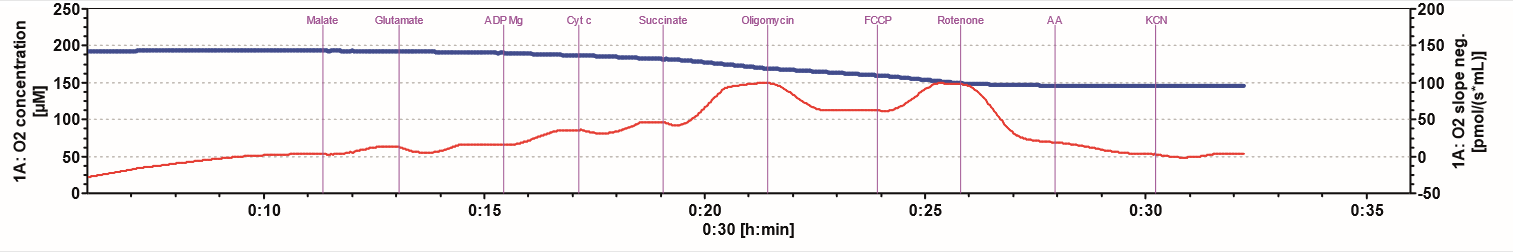


**Figure S45.** Cryopreservation of cardiac muscle homogenate. Homogenate prepared from fresh biopsy, frozen at -80°C, thawed and measured immediately after thawing.


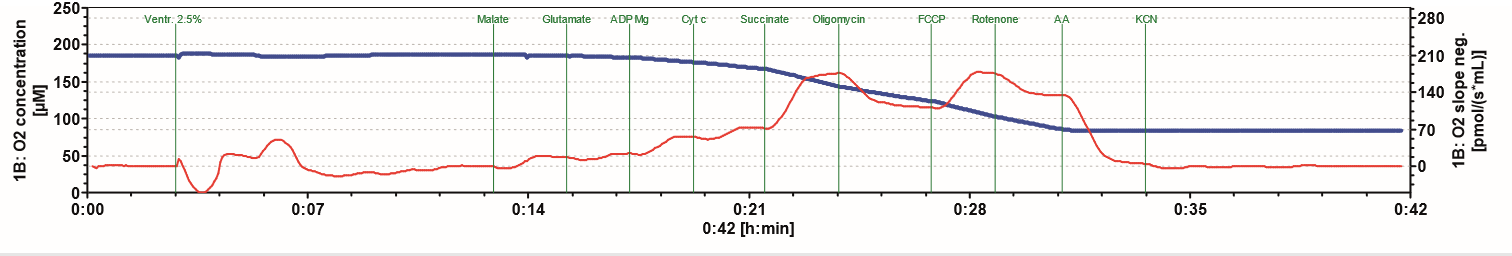


**Figure S46.** Cryopreservation of cardiac muscle biopsy. Muscle tissue was frozen in liquid nitrogen (in BIOPS with 30% DMSO and 10mg/ml BSA, as previously described[3]). After that, muscle tissue was thawed, washed and homogenized in MiR05 and measured immediately after homogenization.

# ISOLATION OF MITOCHONDRIA

Heart mitochondria were isolated as previously described [1,4] with slight modifications. Briefly, the minced heart tissue was manually homogenized in Isolation Medium (225 mM Mannitol, 75 mM Sucrose, 1 mM EGTA, pH 7.4) with glass-glass homogenizer (Wheaton^TM^ 1mL Tissue Grinder, Dounce; Wheaton^TM^, Millville, USA) with loose pestle (larger clearance 0.114 ± 0.025 mm) and by motor-driven homogenization (750 rpmi, HEi-Torque Value 100, Heidolph, Germany) with PTFE pestle (from Wheaton^TM^ 2mL Potter-Elvehjem Tissue Grinder set; Wheaton^TM^, Millville, USA) as described above. The homogenate was filtered through a polyamide mesh and centrifuged at low speed (600 xg, 10 min, 4°C). The supernatant was used for isolation of subsarcolemmal mitochondria and pellet for interfibrillar mitochondria (see **Figure S47**). The subsarcolemmal fraction was obtained by high speed centrifugation (10 000 xg, 10 min, 4°C). The final pellet was resuspended in MiR05 and measured on high resolution respirometry. Interfibrillar mitochondria were derived from initial pellet which was treated in nagarse (at a final concentration of 5 mg / wet weight of tissue) and immediately homogenized with a Potter Elvehjam Homogenizer. The pellet after nagarse treatment was resuspended in the isolation buffer and centrifuged at low speed (600 xg, 10 min, 4°C) to separate nuclear fraction. The remaining supernatant was centrifuged at high speed (10 000 xg, 10 min, 4°C). The resulting pellet contained interfibrillar mitochondria and was resuspended in MiR05 and subsequently measured on high-resolution respirometry.

Muscle sample (minced tissue)

**↓** *Homogenization*

Potter-Elvehjam Homogenate

**↓** *Centrifugation (600 xg)*

**↓** **↓**

SN Pellet

**↓** *Centrifugation (10 000 xg)* **↓** *Nagarse + Centrifugation (600 xg)*

**↓** **↓**  **↓** **↓**

SN Pellet = **SSM** SN Pellet = Nuclear Fraction

**↓** *Centrifugation (10 000 xg)*

**↓ ↓**

SN Pellet = **IFM**

**Figure S47.** Simplified scheme of mitochondrial isolation procedure. Note: SN = supernatant, SSM = subsarcolemmal mitochondria, IFM = interfibrillar mitochondria.


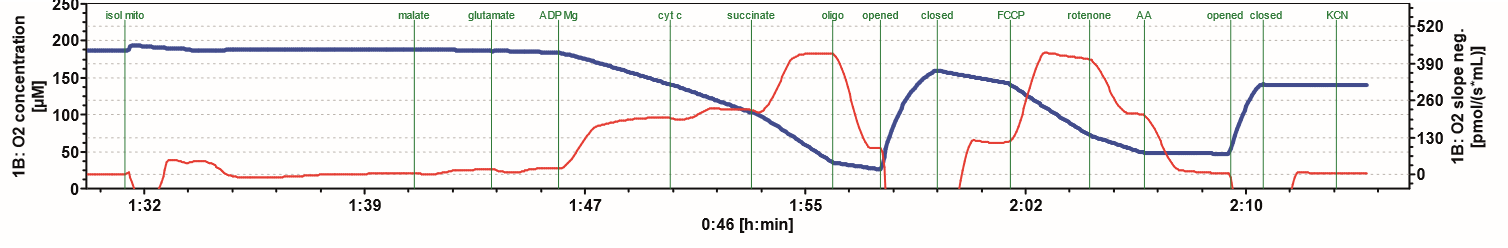


**Figure S48.** Subsarcolemmal mitochondria.


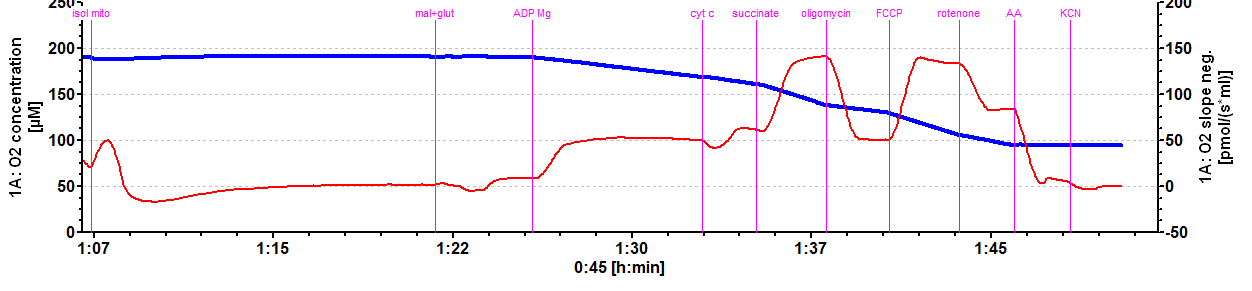


**Figure S49.** Interfibrillar mitochondria.

#

# COMPARISON OF CARDIAC MUSCLE HOMOGENATES WITH ISOLATED MITOCHONDRIA

|  | | Malate+ Glutamate [pmol/(s*ml)] | ADP [pmol/(s*ml)] | Cyt c [pmol/(s*ml)] | % increase post addition of Cytochrome c | Succinate: STATE 3/OXPHOS CAPACITY(P') [pmol/(s*ml)] | Oligomycine: leak respiration [pmol/(s*ml)] | FCCP: ETS capacity (E') [pmol/(s*ml)] | Complex I uncoupled [pmol/(s*ml)] | Antimycine A [pmol/(s*ml)] | Complex I corr. for ROX[pmol/(s*ml)] | CI control ratio | Complex II [pmol/(s*ml)] | CII control ratio | Proton leak [pmol/(s*ml)] | Proton leak [%] | ATP production [%] | ETS capacity (E) corrected for ROX  [pmol/(s*ml)] | Respiratory Control Ratio (RCR) |
| --- | --- | --- | --- | --- | --- | --- | --- | --- | --- | --- | --- | --- | --- | --- | --- | --- | --- | --- | --- |
| Sample: | |  |  |  |  | P' | L' | E' |  | ROX | CI | CI/CII&II | CII | CII/CI&II | |  |  | E | RCR |
| Mean | HOM  (n=3) | 33.5 | 169.3 | 192.3 | 11.7 | 313.6 | 83.5 | 293.7 | 172.3 | 3.3 | 166 | 0.59 | 121.2 | 0.41 | | 26.6 | 73.4 | 290.4 | 3.6 |
| SD |  | 13.1 | 60.5 | 69.6 | 1.8 | 129.3 | 33.8 | 107.6 | 65.3 | 3.9 | 57.4 | 0.04 | 60.4 | 0.04 | | 3.3 | 3.3 | 104.4 | 0.5 |
| **Mean CV [%]** | **HOM**  **(n=3)** | **4.4** | **2.1** | **3.0** | **6.7** | **3.4** | **4.7** | **3.3** | **3.0** | **N/A** | **1.9** | **1.9** | **4.8** | **2.7** | | **4.0** | **1.3** | **3.2** | **2.8** |
| **SD** | **HOM**  **(n=3)** | **2.8** | **1.7** | **2.0** | **6.5** | **1.4** | **4.8** | **4.0** | **3.7** | **N/A** | **1.1** | **1.4** | **3.9** | **2.2** | | **2.3** | **0.7** | **4.0** | **1.9** |
| Mean | SSM  (n=4) | 24.2 | 245.9 | 290.4 | 15.7 | 502.8 | 143.6 | 437.2 | 262.9 | 8.0 | 238 | 0.53 | 212.4 | 0.47 | | 28.8 | 71.2 | 429.2 | 3.0 |
| SD |  | 2.4 | 46.0 | 44.9 | 3.1 | 75.7 | 17.1 | 66.4 | 37.4 | 2.4 | 45.5 | 0.06 | 54.8 | 0.06 | | 2.9 | 2.9 | 66.6 | 0.3 |
| **Mean CV [%]** | **SSM**  **(n=4)** | **6.9** | **6.4** | **5.1** | **7.8** | **5.6** | **9.9** | **8.8** | **9.2** | **N/A** | **6.7** | **2.1** | **6.4** | **2.4** | | **5.6** | **2.2** | **9.0** | **4.1** |
| **SD** | **SSM**  **(n=4)3.2** | **3.6** | **2.3** | **3.5** | **9.6** | **4.0** | **7.5** | **4.5** | **4.6** | **N/A** | **2.5** | **1.0** | **6.0** | **1.3** | | **5.4** | **2.1** | **4.4** | **1.4** |
| Mean | IFM  (n=4) | 8.3 | 52.7 | 59.5 | 11.7 | 111.8 | 40.4 | 107.3 | 63.7 | 5.1 | 47.5 | 0.50 | 52.2 | 0.50 | | 37.6 | 62.4 | 102.2 | 2.7 |
| SD |  | 2.5 | 21.7 | 23.5 | 3.5 | 49.6 | 15.3 | 41.1 | 24.5 | 1.1 | 21.7 | 0.11 | 27.4 | 0.11 | | 4.4 | 4.4 | 40.8 | 0.2 |
| **Mean CV [%]** | **IFM**  **(n=4)** | **16.3** | **11.6** | **13.0** | **16.3** | **18.4** | **18.1** | **14.2** | **15.7** | **N/A** | **13.3** | **9.7** | **30.1** | **13.1** | | **5.7** | **3.6** | **15.5** | **5.9** |
| **SD** | **IFM**  **(n=4)** | **3.2** | **4.7** | **6.3** | **16.3** | **15.1** | **14.3** | **9.9** | **10.1** | **N/A** | **6.4** | **11.8** | **31.2** | **19.8** | | **3.3** | **2.3** | **10.3** | **4.3** |

**Table S1.** **Raw data from duplicate measurements on homogenates (n=3) and isolated subsarcolemmal and interfibrillar mitochondria (both n=4) prepared from left ventricles.** Mitochondrial functional parameters. Note: ETS = Electron Transfer System, OXPHOS = oxidative phosphorylation, HOM = homogenates, SSM = subsarcolemmal mitochondria, IFM = interfibrillar mitochondria, CI = complex I, CII = complex II, ROX = residual oxygen consumption, RCR = respiratory control ratio.

# COMPARISON OF ATRIAL VS VENTRICULAR HOMOGENATES

## 3A. Completed data


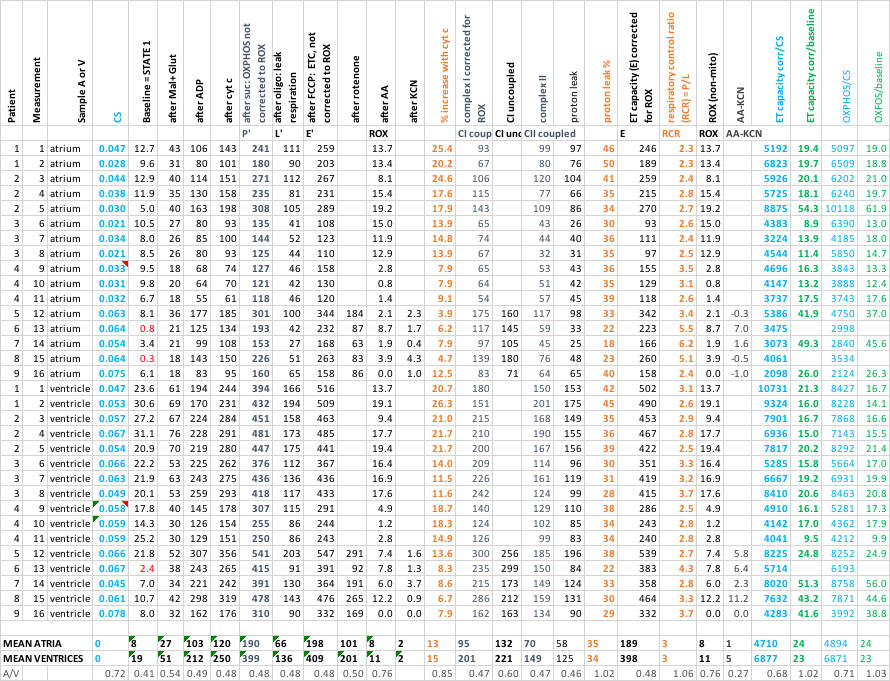


**Table S2: Raw data from comparison of atrial vs. ventricular samples [pmol/s.ml]. Data from 16 measurements from 9 hearts.** Unadjusted data in black, dimensionless indices in orange, data normalised to cityrates synthase activity in blue and to baseline respiration in green.

## 3B. Sex-based differences

| Ventricles | Patient | Sex | CI control ratio | CII control ratio | % leak | RCR |
| --- | --- | --- | --- | --- | --- | --- |
|  | 1 | F | 0,47 | 0,53 | 47,75 | 2,30 |
|  | 2 | F | 0,55 | 0,45 | 36,76 | 2,65 |
|  | 3 | F | 0,64 | 0,36 | 33,81 | 2,50 |
|  | 4 | F | 0,66 | 0,34 | 21,80 | 5,52 |
|  | 5 | F | 0,68 | 0,32 | 17,56 | 6,24 |
|  | 6 | F | 0,65 | 0,35 | 22,79 | 5,12 |
|  | 7 | F | 0,56 | 0,44 | 40,47 | 2,44 |
|  | 1 | M | 0,53 | 0,47 | 36,69 | 3,04 |
|  | 2 | M | 0,60 | 0,40 | 33,35 | 3,42 |
| Atria | 1 | F | 0,49 | 0,51 | 43,55 | 2,86 |
|  | 2 | F | 0,54 | 0,46 | 36,74 | 2,75 |
|  | 3 | F | 0,63 | 0,37 | 29,67 | 3,39 |
|  | 4 | F | 0,61 | 0,39 | 22,04 | 4,27 |
|  | 5 | F | 0,59 | 0,41 | 33,32 | 2,79 |
|  | 6 | F | 0,64 | 0,36 | 29,93 | 3,32 |
|  | 7 | F | 0,55 | 0,45 | 29,04 | 3,69 |
|  | 1 | M | 0,54 | 0,46 | 35,21 | 2,72 |
|  | 2 | M | 0,62 | 0,38 | 37,56 | 2,69 |

# Table S3. Dimensionless parameters from measurements in atrial and ventricular homogenates obtained from female (N = 7) and male patients (N = 2). Data represent means from repeated measurement in those subjects, where experiments were performed in duplicates or triplicates. No significant sex-based differences were observed (p ˃ 0.05). Note: F = female; M = female; CI = complex I; CII = complex II; RCR = respiratory control ratio.

# ELECTRON MICROSCOPY

The isolated mitochondria were fixed with 2 % glutaraldehyde in Sörensen buffer (0.1 M sodium/potassium phosphate buffer, pH 7.3; SB) for 2 hours, washed with SB, embedded in blocks of 1% low-melting point agarose (type VII, Sigma Aldrich), and postfixed with 1% OsO4 solution in SB for 2 hours. The samples were dehydrated in series of acetone with increasing concentration and embedded in Epon-Durcupan resin. Polymerized blocks were cut into 80 nm ultrathin sections, collected on 200 mesh size copper grids, and stained with saturated aqueous solution of uranyl acetate for 4 min. The sections were examined in FEI Morgagni 268 transmission electron microscope operated at 80 kV. The images were captured using Mega View III CCD camera (Olympus Soft Imaging Solutions).

# REFERENCES

1. Pecinová A, Drahota Z, Nůsková H, Pecina P, Houštěk J. Evaluation of basic mitochondrial functions using rat tissue homogenates. Mitochondrion. 2011;11: 722–8. doi:10.1016/j.mito.2011.05.006

2. Ziak J, Krajcova A, Jiroutkova K, Nemcova V, Dzupa V, Duska F. Assessing the function of mitochondria in cytosolic context in human skeletal muscle: Adopting high-resolution respirometry to homogenate of needle biopsy tissue samples. Mitochondrion. Elsevier; 2015;21: 106–112. doi:10.1016/j.mito.2015.02.002

3. Kuznetsov A V, Kunz WS, Saks V, Usson Y. Cryopreservation of mitochondria and mitochondrial function in cardiac and skeletal muscle fibers. Anal Biochem. 2003;2697. doi:10.1016/S0003-2697(03)00326-9

4. Palmer W, Tandler B, Hoppel C. Biochemical Properties of Subsarcolemmal and Interfibrillar Mitochondria Isolated from Rat Cardiac Muscle. J Biol Chem. 1977; 8731–8739. doi:10.1016/0003-9861(85)90675-7
