# Supplementary material for: High resolution respirometry to assess function of mitochondria in native homogenates of human heart muscle
Source: PLoS One. 2020 Jan 15;15(1):e0226142. doi: 10.1371/journal.pone.0226142 (PMC6961865; doi:10.1371/journal.pone.0226142)
Supplement: S1 Data — (ZIP) [file pone.0226142.s003.zip › Analysis_Atrium_vs_Ventricle.docx]

# Ventricle vs atrium

Petr Waldauf

Stata 15.1

8.1.2018

Update: 9.1.2018

## Descriptive statistics

graph box baseline mal glut adp cytc suc oligo fccp aa, ytitle(O2 slope [pmol/(s*ml)]) legend(rows(2)) by(heart)

graph box ci cii leak e, ytitle(O2 slope [pmol/(s*ml)]) legend(rows(1)) by(heart)

graph box ci_control_ratio cii_control_ratio rcr l_p fcr p_e j, ytitle() legend(rows(2)) by(heart)

graph box leak_perc rox, ytitle() legend(rows(2)) by(heart)

|  | **heart** | **N** | **mean** | **sd** | **p25** | **p50** | **p75** | **min** | **max** | **mean diff** | **p** |
| --- | --- | --- | --- | --- | --- | --- | --- | --- | --- | --- | --- |
| baseline | ventricle | 11 | 23.2 | 5.1 | 20.1 | 22.2 | 27.2 | 14.3 | 31.1 | 13.6 | <0.001 |
|  | atrium | 11 | 9.5 | 2.4 | 8.0 | 9.6 | 11.9 | 5.0 | 12.9 |  |  |
|  | Total | 22 | 16.4 | 8.0 | 9.6 | 13.6 | 22.2 | 5.0 | 31.1 |  |  |
| mal | ventricle | 11 | 41.1 | 9.2 | 35.8 | 42.7 | 49.8 | 26.3 | 55.9 | 16.6 | <0.001 |
|  | atrium | 11 | 24.5 | 4.9 | 21.3 | 24.0 | 27.6 | 14.5 | 32.8 |  |  |
|  | Total | 22 | 32.8 | 11.1 | 24.0 | 28.4 | 42.7 | 14.5 | 55.9 |  |  |
| glut | ventricle | 11 | 55.7 | 16.0 | 39.9 | 60.9 | 69.1 | 29.8 | 75.6 | 26.1 | <0.001 |
|  | atrium | 11 | 29.6 | 9.1 | 20.4 | 26.8 | 39.9 | 18.2 | 43.5 |  |  |
|  | Total | 22 | 42.6 | 18.4 | 26.8 | 39.9 | 60.9 | 18.2 | 75.6 |  |  |
| ADP | ventricle | 11 | 196.5 | 47.1 | 144.8 | 219.0 | 227.6 | 125.7 | 259.5 | 103.2 | <0.001 |
|  | atrium | 11 | 93.4 | 32.0 | 68.2 | 80.4 | 113.8 | 55.5 | 162.6 |  |  |
|  | Total | 22 | 144.9 | 65.8 | 80.4 | 129.5 | 219.0 | 55.5 | 259.5 |  |  |
| Cyt C | ventricle | 11 | 240.3 | 54.6 | 178.2 | 262.0 | 283.6 | 151.3 | 293.5 | 127.4 | <0.001 |
|  | atrium | 11 | 112.9 | 43.3 | 74.1 | 100.3 | 151.0 | 61.0 | 198.2 |  |  |
|  | Total | 22 | 176.6 | 81.0 | 100.3 | 155.9 | 262.0 | 61.0 | 293.5 |  |  |
| Succ | ventricle | 11 | 386.2 | 80.4 | 307.3 | 417.8 | 447.2 | 250.0 | 481.0 | 204.0 | <0.001 |
|  | atrium | 11 | 182.3 | 69.0 | 125.1 | 144.0 | 241.1 | 118.3 | 307.6 |  |  |
|  | Total | 22 | 284.3 | 127.4 | 144.0 | 263.0 | 417.8 | 118.3 | 481.0 |  |  |
| Oligo | ventricle | 11 | 138.1 | 37.5 | 111.9 | 136.3 | 173.2 | 85.9 | 194.2 | 68.2 | <0.001 |
|  | atrium | 11 | 70.0 | 29.9 | 44.3 | 51.7 | 105.3 | 40.8 | 112.1 |  |  |
|  | Total | 22 | 104.1 | 48.1 | 51.7 | 107.9 | 136.3 | 40.8 | 194.2 |  |  |
| FCCP | ventricle | 11 | 402.4 | 101.3 | 290.6 | 436.1 | 484.8 | 242.6 | 515.8 | 220.9 | <0.001 |
|  | atrium | 11 | 181.5 | 69.8 | 119.5 | 157.5 | 259.4 | 107.9 | 289.0 |  |  |
|  | Total | 22 | 292.0 | 141.4 | 157.5 | 263.0 | 436.1 | 107.9 | 515.8 |  |  |
| AA | ventricle | 11 | 12.6 | 6.8 | 4.9 | 16.4 | 17.7 | 1.2 | 19.4 | 2.2 | 0.094 |
|  | atrium | 11 | 10.4 | 6.2 | 2.8 | 12.9 | 15.0 | 0.8 | 19.2 |  |  |
|  | Total | 22 | 11.5 | 6.5 | 4.9 | 13.6 | 16.9 | 0.8 | 19.4 |  |  |
| CI | ventricle | 11 | 183.9 | 41.9 | 139.9 | 199.6 | 214.8 | 124.4 | 241.9 | 101.0 | <0.001 |
|  | atrium | 11 | 82.9 | 27.8 | 64.7 | 67.4 | 105.7 | 54.0 | 143.4 |  |  |
|  | Total | 22 | 133.4 | 62.2 | 67.4 | 125.2 | 199.6 | 54.0 | 241.9 |  |  |
| CI control ratio | ventricle | 11 | 0.56 | 0.06 | 0.52 | 0.55 | 0.58 | 0.43 | 0.66 | 0.004 | 0.843 |
|  | atrium | 11 | 0.55 | 0.07 | 0.48 | 0.55 | 0.60 | 0.46 | 0.68 |  |  |
|  | Total | 22 | 0.55 | 0.07 | 0.52 | 0.55 | 0.60 | 0.43 | 0.68 |  |  |
| CII | ventricle | 11 | 146.0 | 34.9 | 114.2 | 150.0 | 167.8 | 98.7 | 201.4 | 76.6 | <0.001 |
|  | atrium | 11 | 69.4 | 29.4 | 43.7 | 57.2 | 98.6 | 31.9 | 119.7 |  |  |
|  | Total | 22 | 107.7 | 50.3 | 57.2 | 105.5 | 150.0 | 31.9 | 201.4 |  |  |
| CII control ratio | ventricle | 11 | 0.44 | 0.06 | 0.42 | 0.45 | 0.48 | 0.34 | 0.57 | -0.004 | 0.843 |
|  | atrium | 11 | 0.45 | 0.07 | 0.40 | 0.45 | 0.52 | 0.32 | 0.54 |  |  |
|  | Total | 22 | 0.45 | 0.07 | 0.40 | 0.45 | 0.48 | 0.32 | 0.57 |  |  |
| Leak | ventricle | 11 | 248.1 | 53.7 | 191.9 | 264.3 | 299.5 | 164.1 | 307.8 | 135.8 | <0.001 |
|  | atrium | 11 | 112.3 | 42.6 | 80.8 | 92.3 | 153.5 | 72.2 | 202.3 |  |  |
|  | Total | 22 | 180.2 | 84.1 | 92.3 | 166.7 | 264.3 | 72.2 | 307.8 |  |  |
| Leak % | ventricle | 11 | 64.4 | 5.1 | 60.8 | 65.0 | 68.7 | 55.1 | 72.0 | 2.3 | 0.068 |
|  | atrium | 11 | 62.1 | 5.7 | 58.6 | 64.1 | 65.4 | 50.3 | 69.9 |  |  |
|  | Total | 22 | 63.2 | 5.4 | 60.8 | 64.3 | 65.8 | 50.3 | 72.0 |  |  |
| E | ventricle | 11 | 389.8 | 95.8 | 285.7 | 419.2 | 467.1 | 239.8 | 502.1 | 218.7 | <0.001 |
|  | atrium | 11 | 171.1 | 67.3 | 111.0 | 154.8 | 245.6 | 92.9 | 269.8 |  |  |
|  | Total | 22 | 280.4 | 138.0 | 154.8 | 252.1 | 419.2 | 92.9 | 502.1 |  |  |
| RCR | ventricle | 11 | 1.62 | 0.27 | 1.46 | 1.54 | 1.65 | 1.39 | 2.37 | -0.004 | 0.947 |
|  | atrium | 11 | 1.63 | 0.16 | 1.53 | 1.56 | 1.71 | 1.43 | 1.99 |  |  |
|  | Total | 22 | 1.62 | 0.22 | 1.52 | 1.56 | 1.65 | 1.39 | 2.37 |  |  |
| L/P | ventricle | 11 | 0.36 | 0.05 | 0.31 | 0.35 | 0.39 | 0.28 | 0.45 | -0.023 | 0.068 |
|  | atrium | 11 | 0.38 | 0.06 | 0.35 | 0.36 | 0.41 | 0.30 | 0.50 |  |  |
|  | Total | 22 | 0.37 | 0.05 | 0.34 | 0.36 | 0.39 | 0.28 | 0.50 |  |  |
| FCR | ventricle | 11 | 0.97 | 0.09 | 0.97 | 1.00 | 1.03 | 0.76 | 1.06 | -0.044 | 0.263 |
|  | atrium | 11 | 1.02 | 0.13 | 0.93 | 1.01 | 1.14 | 0.80 | 1.26 |  |  |
|  | Total | 22 | 1.00 | 0.11 | 0.93 | 1.01 | 1.05 | 0.76 | 1.26 |  |  |
| P/E | ventricle | 11 | 0.97 | 0.09 | 0.97 | 1.00 | 1.03 | 0.76 | 1.06 | -0.044 | 0.263 |
|  | atrium | 11 | 1.02 | 0.13 | 0.93 | 1.01 | 1.14 | 0.80 | 1.26 |  |  |
|  | Total | 22 | 1.00 | 0.11 | 0.93 | 1.01 | 1.05 | 0.76 | 1.26 |  |  |
| J | ventricle | 11 | 0.64 | 0.05 | 0.61 | 0.65 | 0.69 | 0.55 | 0.72 | 0.023 | 0.070 |
|  | atrium | 11 | 0.62 | 0.06 | 0.59 | 0.64 | 0.65 | 0.50 | 0.70 |  |  |
|  | Total | 22 | 0.63 | 0.05 | 0.61 | 0.65 | 0.66 | 0.50 | 0.72 |  |  |
| ROX | ventricle | 11 | 12.6 | 6.8 | 4.9 | 16.4 | 17.8 | 1.2 | 19.5 | 2.2 | 0.094 |
|  | atrium | 11 | 10.4 | 6.2 | 2.8 | 12.9 | 15.0 | 0.8 | 19.2 |  |  |
|  | Total | 22 | 11.5 | 6.5 | 4.9 | 13.6 | 16.9 | 0.8 | 19.5 |  |  |

## Hodnoty korigované na baseline

graph box mal_b glut_b adp_b cytc_b suc_b oligo_b fccp_b, ytitle() legend(rows(3)) by(heart)

tabstat aa_b , by(heart) stat (n mean sd p25 p50 p75 min max) col(stat) format(%9.3g)

regress aa_b i.id i.heart i.measurement

| **O2 slope** | **heart** | **N** | **mean** | **sd** | **p25** | **p50** | **p75** | **min** | **max** | **mean diff** | **p** |
| --- | --- | --- | --- | --- | --- | --- | --- | --- | --- | --- | --- |
| mal/baseline | ventricle | 11 | 1.80 | 0.30 | 1.61 | 1.84 | 2.07 | 1.13 | 2.11 | -0.660 | 0.002 |
|  | atrium | 10 | 2.46 | 0.46 | 2.30 | 2.38 | 2.80 | 1.52 | 3.17 |  |  |
|  | Total | 21 | 2.11 | 0.50 | 1.83 | 2.10 | 2.33 | 1.13 | 3.17 |  |  |
| glut/baseline | ventricle | 11 | 2.41 | 0.53 | 2.24 | 2.43 | 2.65 | 1.21 | 3.34 | -0.420 | 0.018 |
|  | atrium | 10 | 2.83 | 0.51 | 2.56 | 2.99 | 3.25 | 1.91 | 3.43 |  |  |
|  | Total | 21 | 2.61 | 0.55 | 2.26 | 2.58 | 3.04 | 1.21 | 3.43 |  |  |
| adp/baseline | ventricle | 11 | 8.73 | 2.32 | 7.33 | 8.24 | 10.50 | 5.12 | 12.90 | 0.100 | 0.979 |
|  | atrium | 10 | 8.63 | 1.41 | 7.63 | 8.39 | 9.42 | 6.58 | 10.90 |  |  |
|  | Total | 21 | 8.68 | 1.89 | 7.63 | 8.39 | 10.20 | 5.12 | 12.90 |  |  |
| cytc/baseline | ventricle | 11 | 10.60 | 2.47 | 9.36 | 10.40 | 12.50 | 6.01 | 14.60 | 0.300 | 0.853 |
|  | atrium | 10 | 10.30 | 2.03 | 8.86 | 10.70 | 11.70 | 7.15 | 13.30 |  |  |
|  | Total | 21 | 10.50 | 2.22 | 9.07 | 10.50 | 11.80 | 6.01 | 14.60 |  |  |
| suc/baseline | ventricle | 11 | 17.00 | 3.21 | 15.50 | 17.00 | 19.90 | 9.93 | 21.40 | 0.200 | 0.983 |
|  | atrium | 10 | 16.80 | 3.14 | 13.30 | 17.80 | 19.00 | 12.40 | 21.00 |  |  |
|  | Total | 21 | 16.90 | 3.10 | 14.70 | 17.30 | 19.00 | 9.93 | 21.40 |  |  |
| oligo/baseline | ventricle | 11 | 6.02 | 1.23 | 5.58 | 6.03 | 6.49 | 3.41 | 8.38 | -0.500 | 0.322 |
|  | atrium | 10 | 6.52 | 1.95 | 4.80 | 6.64 | 8.72 | 3.90 | 9.35 |  |  |
|  | Total | 21 | 6.25 | 1.59 | 5.20 | 6.23 | 6.85 | 3.41 | 9.35 |  |  |
| fccp/baseline | ventricle | 11 | 17.60 | 3.50 | 16.30 | 17.00 | 21.10 | 9.64 | 21.90 | 0.800 | 0.704 |
|  | atrium | 10 | 16.80 | 3.75 | 13.30 | 17.20 | 20.50 | 10.30 | 21.10 |  |  |
|  | Total | 21 | 17.20 | 3.55 | 15.60 | 17.00 | 20.50 | 9.64 | 21.90 |  |  |
| aa/baseline | ventricle | 11 | 0.54 | 0.29 | 0.28 | 0.58 | 0.77 | 0.09 | 0.93 | -0.406 | 0.001 |
|  | atrium | 10 | 0.94 | 0.58 | 0.29 | 1.19 | 1.43 | 0.08 | 1.51 |  |  |
|  | Total | 21 | 0.73 | 0.49 | 0.29 | 0.63 | 1.08 | 0.08 | 1.51 |  |  |

## Vzestup po cyt C

Výpočet : 100*(after Cyt c - after ADP)/(after ADP)

tabstat cytc_perc , by(heart) stat (n mean sd p25 p50 p75 min max) col(stat) format(%9.3g)

| Cyc C % | **heart** | **N** | **mean** | **sd** | **p25** | **p50** | **p75** | **min** | **max** | **mean diff** | **p** |
| --- | --- | --- | --- | --- | --- | --- | --- | --- | --- | --- | --- |
|  | ventricle | 11 | 22.6% | 7.1% | 16.3% | 23.1% | 27.8% | 13.0% | 35.6% | 3.3% | 0.119 |
|  | atrium | 11 | 19.3% | 8.9% | 10.1% | 17.4% | 25.4% | 8.6% | 34.1% |  |  |
|  | Total | 22 | 21.0% | 8.0% | 16.1% | 21.6% | 26.6% | 8.6% | 35.6% |  |  |

graph box cytc_perc, ytitle(Cyt C - perc) legend(rows(2)) by(heart, total rows(1))

## Koeficienty variability

mean baseline – rox

### Atrium

| **Atrium** | cv mean | cv sd | cv stand.error | cv dolní 95% CI | cv horní 95% CI |
| --- | --- | --- | --- | --- | --- |
| **baseline** | 24.2% | 13.0% | 6.5% | 3.5% | 45.0% |
| **mal** | 11.7% | 8.9% | 4.5% | -2.5% | 25.9% |
| **glut** | 9.9% | 9.4% | 4.7% | -5.0% | 24.8% |
| **adp** | 13.1% | 7.3% | 3.7% | 1.4% | 24.8% |
| **cytc** | 13.4% | 8.4% | 4.2% | 0.0% | 26.8% |
| **cytc_perc** | 14.9% | 9.8% | 4.9% | -0.6% | 30.4% |
| **suc** | 11.1% | 7.4% | 3.7% | -0.8% | 22.9% |
| **oligo** | 12.0% | 5.3% | 2.6% | 3.6% | 20.3% |
| **fccp** | 12.5% | 4.4% | 2.2% | 5.5% | 19.6% |
| **aa** | 28.5% | 27.0% | 13.5% | -14.5% | 71.5% |
| **ci** | 13.9% | 7.1% | 3.5% | 2.6% | 25.2% |
| **ci_control_ratio** | 7.5% | 3.6% | 1.8% | 1.9% | 13.2% |
| **cii** | 14.9% | 6.7% | 3.4% | 4.2% | 25.6% |
| **cii_control_ratio** | 9.5% | 4.7% | 2.4% | 1.9% | 17.0% |
| **leak** | 13.8% | 8.8% | 4.4% | -0.1% | 27.8% |
| **leak_perc** | 4.9% | 1.3% | 0.7% | 2.9% | 7.0% |
| **E** | 13.4% | 3.9% | 1.9% | 7.2% | 19.5% |
| **RCR** | 5.0% | 1.4% | 0.7% | 2.8% | 7.2% |
| **L_P** | 8.0% | 2.7% | 1.4% | 3.6% | 12.3% |
| **FCR** | 5.3% | 3.6% | 1.8% | -0.4% | 11.0% |
| **P_E** | 5.3% | 3.6% | 1.8% | -0.4% | 11.0% |
| ***J*** | 4.9% | 1.3% | 0.7% | 2.9% | 7.0% |
| **ROX** | 28.5% | 27.0% | 13.5% | -14.5% | 71.5% |

### Ventricle

| **Ventricle** | cv mean | cv sd | cv stand.error | cv dolní 95% CI | cv horní 95% CI |
| --- | --- | --- | --- | --- | --- |
| **baseline** | 18.0% | 9.9% | 4.9% | 2.3% | 33.7% |
| **mal** | 11.3% | 3.9% | 2.0% | 5.0% | 17.5% |
| **glut** | 10.4% | 4.7% | 2.4% | 2.9% | 17.9% |
| **adp** | 6.4% | 3.1% | 1.6% | 1.5% | 11.4% |
| **cytc** | 5.2% | 3.1% | 1.5% | 0.3% | 10.1% |
| **cytc_perc** | 13.0% | 7.9% | 4.0% | 0.4% | 25.7% |
| **suc** | 7.4% | 3.2% | 1.6% | 2.3% | 12.5% |
| **oligo** | 11.2% | 5.0% | 2.5% | 3.3% | 19.1% |
| **fccp** | 6.4% | 4.4% | 2.2% | -0.7% | 13.5% |
| **aa** | 30.8% | 24.4% | 12.2% | -8.0% | 69.7% |
| **ci** | 7.5% | 3.6% | 1.8% | 1.8% | 13.1% |
| **ci_control_ratio** | 7.7% | 6.3% | 3.2% | -2.4% | 17.7% |
| **cii** | 15.5% | 5.8% | 2.9% | 6.2% | 24.7% |
| **cii_control_ratio** | 8.9% | 5.7% | 2.9% | -0.1% | 18.0% |
| **leak** | 6.2% | 2.3% | 1.1% | 2.6% | 9.9% |
| **leak_perc** | 3.1% | 0.5% | 0.3% | 2.3% | 4.0% |
| **E** | 6.7% | 4.0% | 2.0% | 0.4% | 13.0% |
| **RCR** | 7.0% | 7.9% | 3.9% | -5.6% | 19.5% |
| **L_P** | 5.5% | 0.7% | 0.4% | 4.3% | 6.6% |
| **FCR** | 3.4% | 2.8% | 1.4% | -1.0% | 7.8% |
| **P_E** | 3.4% | 2.8% | 1.4% | -1.0% | 7.8% |
| ***J*** | 3.1% | 0.5% | 0.3% | 2.3% | 4.0% |
| **ROX** | 30.8% | 24.4% | 12.2% | -8.0% | 69.7% |

# O_2_ concentration

graph box baseline mal glut adp cytc suc oligo fccp aa, ytitle(O2 concetration [μM]) legend(rows(2)) by(heart)

| **O2 conc** | **heart** | **N** | **mean** | **sd** | **p25** | **p50** | **p75** | **min** | **max** | **mean diff** | **p** |
| --- | --- | --- | --- | --- | --- | --- | --- | --- | --- | --- | --- |
| baseline | ventricle | 11 | 181.6 | 3.1 | 178.9 | 181.2 | 183.5 | 177.1 | 186.9 | -7.6 | <0.001 |
|  | atrium | 11 | 189.2 | 3.4 | 185.9 | 187.5 | 192.3 | 185.7 | 195.0 |  |  |
|  | Total | 22 | 185.4 | 5.0 | 181.2 | 185.7 | 187.5 | 177.1 | 195.0 |  |  |
| mal | ventricle | 11 | 177.4 | 3.6 | 173.9 | 177.4 | 180.2 | 173.0 | 183.8 | -9.3 | <0.001 |
|  | atrium | 11 | 186.8 | 3.6 | 183.3 | 185.3 | 190.8 | 183.1 | 192.7 |  |  |
|  | Total | 22 | 182.1 | 5.9 | 177.4 | 183.2 | 185.3 | 173.0 | 192.7 |  |  |
| glut | ventricle | 11 | 169.6 | 5.6 | 165.1 | 168.1 | 175.1 | 162.8 | 179.7 | -13.7 | <0.001 |
|  | atrium | 11 | 183.4 | 4.3 | 179.7 | 181.8 | 188.4 | 178.1 | 190.7 |  |  |
|  | Total | 22 | 176.5 | 8.6 | 168.1 | 178.5 | 181.8 | 162.8 | 190.7 |  |  |
| ADP | ventricle | 11 | 144.0 | 11.0 | 136.0 | 138.6 | 158.5 | 134.5 | 162.9 | -27.3 | <0.001 |
|  | atrium | 11 | 171.3 | 8.6 | 165.2 | 169.9 | 180.9 | 156.1 | 184.0 |  |  |
|  | Total | 22 | 157.7 | 17.0 | 138.6 | 162.1 | 169.9 | 134.5 | 184.0 |  |  |
| Cyt C | ventricle | 11 | 115.6 | 15.4 | 103.4 | 108.0 | 137.5 | 102.6 | 139.5 | -42.0 | <0.001 |
|  | atrium | 11 | 157.6 | 12.8 | 147.3 | 156.1 | 172.2 | 133.9 | 175.7 |  |  |
|  | Total | 22 | 136.6 | 25.5 | 108.0 | 139.3 | 156.1 | 102.6 | 175.7 |  |  |
| Succ | ventricle | 11 | 91.6 | 17.0 | 77.9 | 96.1 | 108.1 | 56.6 | 109.1 | -40.3 | <0.001 |
|  | atrium | 11 | 132.0 | 23.3 | 120.7 | 131.4 | 154.9 | 78.7 | 159.3 |  |  |
|  | Total | 22 | 111.8 | 28.7 | 95.7 | 108.7 | 131.4 | 56.6 | 159.3 |  |  |
| Oligo | ventricle | 11 | 138.3 | 9.6 | 133.0 | 138.6 | 146.4 | 120.1 | 151.4 | -2.8 | 0.6 |
|  | atrium | 11 | 141.1 | 15.4 | 129.9 | 144.5 | 154.1 | 112.2 | 157.3 |  |  |
|  | Total | 22 | 139.7 | 12.6 | 133.0 | 140.4 | 151.4 | 112.2 | 157.3 |  |  |
| FCCP | ventricle | 11 | 77.8 | 26.2 | 53.8 | 74.9 | 97.7 | 39.3 | 124.1 | -34.9 | 0.001 |
|  | atrium | 11 | 112.6 | 22.8 | 100.1 | 121.3 | 130.1 | 53.7 | 133.0 |  |  |
|  | Total | 22 | 95.2 | 29.9 | 73.3 | 99.7 | 122.6 | 39.3 | 133.0 |  |  |
| AA | ventricle | 11 | 75.9 | 35.7 | 49.1 | 62.2 | 90.7 | 28.4 | 135.5 | -25.9 | 0.014 |
|  | atrium | 11 | 101.9 | 24.1 | 91.2 | 105.6 | 120.5 | 43.0 | 125.1 |  |  |
|  | Total | 22 | 88.9 | 32.5 | 57.8 | 91.0 | 116.6 | 28.4 | 135.5 |  |  |
