# Supplementary material for: High resolution respirometry to assess function of mitochondria in native homogenates of human heart muscle
Source: PLoS One. 2020 Jan 15;15(1):e0226142. doi: 10.1371/journal.pone.0226142 (PMC6961865; doi:10.1371/journal.pone.0226142)
Supplement: S1 Data — (ZIP) [file pone.0226142.s003.zip › Analysis_ComparisonAtrVentrHeartHomConsolidated patients 1-9.docx]

# Comparison Atr Ventr Heart Hom Consolidated patients 1-9

Petr Waldauf

Stata 15.1

Created: 06.10.2018

Updated: 01.11.2018 (corrected CS)

label define sample 0 "atrium" 1 "ventricle"

encode sample0, gen (sample)

reshape wide cs - oxphos_baseline , i( measurement ) j( sample )

# Descriptive statistics

## Atrium

tabstat cs - u_cs_baseline if sample ==0 , stat(n mean sd p25 p50 p75 min max) columns(statistics) format(%9.4g)

| **variable** | **N** | **mean** | **sd** | **p25** | **p50** | **p75** | **min** | **max** |
| --- | --- | --- | --- | --- | --- | --- | --- | --- |
| **CS** | 16 | 0.03288 | 0.007356 | 0.029 | 0.032 | 0.036 | 0.021 | 0.047 |
| **Baseline = STATE 1** | 16 | 7.737 | 3.842 | 5.55 | 8.3 | 10.15 | 0.3 | 12.9 |
| **after Mal+ Glut** | 16 | 27.38 | 8.943 | 19 | 26 | 35.5 | 18 | 43 |
| **after ADP** | 16 | 103.3 | 35.96 | 80 | 92 | 127.5 | 55 | 177 |
| **after cyt c** | 16 | 119.6 | 41.17 | 93 | 104.5 | 150.5 | 61 | 198 |
| **after suc: STATE 3/OXPHOS CAPACITY (P') non corrected for ROX** | 16 | 189.9 | 65.52 | 131 | 170 | 238 | 118 | 308 |
| **after oligo: leak respiration** | 16 | 65.94 | 29.01 | 43 | 51.5 | 95 | 27 | 112 |
| **after FCCP: ET capacity (E') non-corrected for ROX** | 16 | 197.7 | 73.42 | 126.5 | 185.5 | 261 | 108 | 344 |
| **after rotenone** | 5 | 100.6 | 47.64 | 83 | 86 | 87 | 63 | 184 |
| **after AA** | 16 | 8.2 | 6.352 | 2 | 8.4 | 13.55 | 0 | 19.2 |
| **after KCN** | 5 | 1.94 | 1.501 | 1 | 1.7 | 2.3 | 0.4 | 4.3 |
| **% increase with cyt c** | 16 | 13.03 | 6.741 | 7.9 | 13.2 | 17.75 | 3.9 | 25.4 |
| **complex I corrected for ROX** | 16 | 95.25 | 34.88 | 66 | 88 | 116 | 54 | 175 |
| **CI uncoupled** | 5 | 132.2 | 43.89 | 105 | 145 | 160 | 71 | 180 |
| **complex II** | 16 | 70.38 | 27.93 | 48 | 61.5 | 89.5 | 32 | 120 |
| proton leak | 16 | 57.81 | 27.08 | 36.5 | 46.5 | 81 | 25 | 104 |
| **proton leak %** | 16 | 34.56 | 8.382 | 31.5 | 35 | 39.5 | 18 | 50 |
| **ET capacity (E) corrected for ROX** | 16 | 189.4 | 73.05 | 123.5 | 177.5 | 252.5 | 93 | 342 |
| **respiratory control ratio (RCR) = P/L** | 16 | 3.237 | 1.243 | 2.4 | 2.65 | 3.45 | 2.3 | 6.2 |
| **ROX (non-mito)** | 16 | 8.2 | 6.352 | 2 | 8.4 | 13.55 | 0 | 19.2 |
| **AA-KCN** | 5 | 1.36 | 3.303 | -0.5 | -0.3 | 1.6 | -1 | 7 |
| **ET capacity corr/CS** | 16 | 5732 | 1786 | 4464 | 5219 | 7407 | 3224 | 8875 |
| **ET capacity corr/baseline** | 14 | 23.57 | 14.35 | 13.9 | 18.75 | 26 | 8.9 | 54.3 |
| OXPHOS/CS | 16 | 5813 | 1674 | 4456 | 6026 | 6778 | 3743 | 10118 |
| OXFOS/baseline | 14 | 24.16 | 14.38 | 14.7 | 18.9 | 26.3 | 12.4 | 61.9 |
| 3p/3u | 16 | 1.035 | 0.1812 | 0.9 | 0.99 | 1.115 | 0.82 | 1.46 |
| Complex II respiration [%] | 16 | 0.3688 | 0.06534 | 0.3114 | 0.3713 | 0.4188 | 0.2552 | 0.4838 |
| Complex I respiration [%] | 5 | 0.5707 | 0.1052 | 0.4644 | 0.6244 | 0.6271 | 0.4528 | 0.6847 |
| 3p/CS | 16 | 5813 | 1674 | 4456 | 6026 | 6778 | 3743 | 10118 |
| 3u/CS | 16 | 5732 | 1786 | 4464 | 5219 | 7407 | 3224 | 8875 |

## Ventricle

tabstat cs - u_cs if sample ==1 , stat(n mean sd p25 p50 p75 min max) columns(statistics) format(%9.4g)

| **variable** | **N** | **mean** | **sd** | **p25** | **p50** | **p75** | **min** | **max** |
| --- | --- | --- | --- | --- | --- | --- | --- | --- |
| **CS** | 16 | 0.05638 | 0.005795 | 0.0525 | 0.0565 | 0.059 | 0.047 | 0.067 |
| **Baseline = STATE 1** | 16 | 19.05 | 8.454 | 12.5 | 21.35 | 24.4 | 2.4 | 31.1 |
| **after Mal+ Glut** | 16 | 50.63 | 15.75 | 36 | 52.5 | 65 | 30 | 76 |
| **after ADP** | 16 | 212.1 | 54.54 | 166 | 222.5 | 243 | 126 | 307 |
| **after cyt c** | 16 | 250.1 | 59.28 | 204.5 | 263.5 | 287.5 | 151 | 356 |
| **after suc: STATE 3/OXPHOS CAPACITY (P') non corrected for ROX** | 16 | 398.9 | 82.12 | 343 | 416.5 | 449 | 250 | 541 |
| **after oligo: leak respiration** | 16 | 135.9 | 38.93 | 101.5 | 133 | 169.5 | 86 | 203 |
| **after FCCP: ET capacity (E') non-corrected for ROX** | 16 | 408.6 | 94.78 | 348 | 434.5 | 480.5 | 243 | 547 |
| **after rotenone** | 5 | 201.6 | 79.37 | 169 | 191 | 265 | 92 | 291 |
| **after AA** | 16 | 10.78 | 6.677 | 5.45 | 10.8 | 17.25 | 0 | 19.4 |
| **after KCN** | 5 | 1.5 | 1.369 | 0.9 | 1.3 | 1.6 | 0 | 3.7 |
| **% increase with cyt c** | 16 | 15.34 | 6.003 | 10.05 | 14.45 | 20.85 | 6.7 | 26.3 |
| **complex I corrected for ROX** | 16 | 201.3 | 52.13 | 156.5 | 209.5 | 230.5 | 124 | 300 |
| **CI uncoupled** | 5 | 220.6 | 57.13 | 173 | 212 | 256 | 163 | 299 |
| **complex II** | 16 | 148.9 | 30.33 | 126.5 | 150 | 167.5 | 99 | 201 |
| proton leak | 16 | 125.3 | 35.45 | 93 | 121.5 | 154 | 83 | 196 |
| **proton leak %** | 16 | 34 | 5.739 | 30 | 34 | 38 | 22 | 45 |
| **ET capacity (E) corrected for ROX** | 16 | 397.8 | 90.38 | 341.5 | 417 | 465.5 | 240 | 539 |
| **respiratory control ratio (RCR) = P/L** | 16 | 3.063 | 0.5005 | 2.75 | 2.85 | 3.3 | 2.5 | 4.3 |
| **ROX (non-mito)** | 16 | 10.78 | 6.677 | 5.45 | 10.8 | 17.25 | 0 | 19.4 |
| **AA-KCN** | 5 | 5.14 | 4.279 | 2.3 | 5.8 | 6.4 | 0 | 11.2 |
| **ET capacity corr/CS** | 16 | 7154 | 1915 | 5844 | 7308 | 8155 | 4041 | 10731 |
| **ET capacity corr/baseline** | 15 | 23.22 | 12.13 | 16 | 19.2 | 24.8 | 9.5 | 51.3 |
| OXPHOS/CS | 16 | 7143 | 1598 | 5816 | 7645 | 8308 | 4212 | 9623 |
| OXFOS/baseline | 15 | 23.43 | 12.83 | 16.6 | 17.9 | 24.9 | 9.9 | 56 |
| 3p/3u | 16 | 1.011 | 0.08098 | 1 | 1.035 | 1.065 | 0.79 | 1.09 |
| Complex II respiration [%] | 16 | 0.3763 | 0.04404 | 0.352 | 0.3774 | 0.3966 | 0.2975 | 0.4658 |
| Complex I respiration [%] | 5 | 0.5289 | 0.1327 | 0.4675 | 0.4765 | 0.4919 | 0.4443 | 0.7642 |
| 3p/CS | 16 | 7143 | 1598 | 5816 | 7645 | 8308 | 4212 | 9623 |
| 3u/CS | 16 | 7154 | 1915 | 5844 | 7308 | 8155 | 4041 | 10731 |

## Graphs

graph box cs, over(sample, label(labsize(large))) ytitle(, size(large))

graph box baseline , over(sample, label(labsize(large))) ytitle(, size(large))

graph box mal_glut , over(sample, label(labsize(large))) ytitle(, size(large))

graph box adp , over(sample, label(labsize(large))) ytitle(, size(large))

graph box cyt_c , over(sample, label(labsize(large))) ytitle(, size(large))

graph box p , over(sample, label(labsize(large))) ytitle(, size(large))

graph box l , over(sample, label(labsize(large))) ytitle(, size(large))

graph box e, over(sample, label(labsize(large))) ytitle(, size(large))

graph box rot, over(sample, label(labsize(large))) ytitle(, size(large))

graph box rox , over(sample, label(labsize(large))) ytitle(, size(large))

graph box kcn , over(sample, label(labsize(large))) ytitle(, size(large))

graph box cyt_c_perc , over(sample, label(labsize(large))) ytitle(, size(large))

graph box ci_coupled , over(sample, label(labsize(large))) ytitle(, size(large))

graph box ci_uncoupled , over(sample, label(labsize(large))) ytitle(, size(large))

graph box ciicoupled , over(sample, label(labsize(large))) ytitle(, size(large))

graph box leak , over(sample, label(labsize(large))) ytitle(, size(large))

graph box leak_perc , over(sample, label(labsize(large))) ytitle(, size(large))

graph box etc , over(sample, label(labsize(large))) ytitle(, size(large))

graph box rcr , over(sample, label(labsize(large))) ytitle(, size(large))

graph box roxnonmito , over(sample, label(labsize(large))) ytitle(, size(large))

graph box aa_kcn , over(sample, label(labsize(large))) ytitle(, size(large))

graph box etc_cs , over(sample, label(labsize(large))) ytitle(, size(large))

graph box etc_baseline , over(sample, label(labsize(large))) ytitle(, size(large))

graph box oxphos_cs , over(sample, label(labsize(large))) ytitle(, size(large))

graph box oxphos_baseline , over(sample, label(labsize(large))) ytitle(, size(large))

graph box p_3u, over(sample, label(labsize(large))) ytitle(, size(large))

graph box cii_perc, over(sample, label(labsize(large))) ytitle(, size(large))

graph box ci_perc, over(sample, label(labsize(large))) ytitle(, size(large))

graph box p_cs, over(sample, label(labsize(large))) ytitle(, size(large))

graph box u_cs, over(sample, label(labsize(large))) ytitle(, size(large))

# Atrium vs Ventricle (linear mixed effect models)

## CS - opraveno

graph box cs, over(sample, label(labsize(large))) ytitle(, size(large))

tabstat cs, by(sample) stat(n mean sd p25 p50 p75 min max) columns(statistics) format(%9.4g)

mixed cs i.sample|| patient:sample, cov(unstructured)

margins sample, plot(ytitle(CS (mean, 95% CI)) ytitle(, size(large)) xtitle(, size(large)) xlabel(, labsize(large)) xmtick(-0.25 0 1 1.25) title(Linear mixed effect model))

Komory mají v průměru o .0231 více CS než síně (p<0.001)

Mixed effect model má problém s konvergencí v random části při velmi nízkých hodnotách CS. Proto ho analyzuji znovu s CS vynásobeným hodnotu 1000:

gen cs1000 = 1000 * cs

mixed cs1000 i.sample|| patient:sample, cov(unstructured)

Model již nyní konvergoval bez problémů…

## CS pouze pacient 5-9

graph box cs_5_9, over(sample, label(labsize(large))) ytitle(, size(large))

tabstat cs_5_9, by(sample) stat(n mean sd p25 p50 p75 min max) columns(statistics) format(%9.4g)

mixed cs_5_9 i.sample|| patient:sample, cov(unstructured)

margins sample, plot(ytitle(CS patient 5-9 (mean, 95% CI)) ytitle(, size(large)) xtitle(, size(large)) xlabel(, labsize(large)) xmtick(-0.25 0 1 1.25) title(Linear mixed effect model))

Komory (u pacienta 5-9) mají v průměru o .0206 více CS než síně (p<0.001)

## after suc: 3p/STATE 3/OXPHOS CAPACITY (P') non corrected for ROX

graph box p, over(sample, label(labsize(large))) ytitle(, size(large))

tabstat p, by(sample) stat(n mean sd p25 p50 p75 min max) columns(statistics) format(%9.4g)

mixed p i.sample|| patient:sample, cov(unstructured)

margins sample, plot(ytitle(P (mean, 95% CI)) ytitle(, size(large)) xtitle(, size(large)) xlabel(, labsize(large)) xmtick(-0.25 0 1 1.25) title(Linear mixed effect model))

Komory mají v průměru o 212 cyšší hodnotu P' než síně (p=0.012)

## % increase with cyt c

graph box cyt_c_perc, over(sample, label(labsize(large))) ytitle(, size(large))

tabstat cyt_c_perc, by(sample) stat(n mean sd p25 p50 p75 min max) columns(statistics) format(%9.4g)

mixed cyt_c_perc i.sample|| patient:sample, cov(unstructured)

margins sample, plot(ytitle(CYT_C_PERC (mean, 95% CI)) ytitle(, size(large)) xtitle(, size(large)) xlabel(, labsize(large)) xmtick(-0.25 0 1 1.25) title(Linear mixed effect model))

Komory mají v průměru o 2.26 vyšší hodnotu CYT_C_PERC než síně, rozdíl ale nedosahuje statistické signifikance (p=0.12)

## complex I corrected for ROX

graph box ci_coupled, over(sample, label(labsize(large))) ytitle(, size(large))

tabstat ci_coupled, by(sample) stat(n mean sd p25 p50 p75 min max) columns(statistics) format(%9.4g)

mixed ci_coupled i.sample|| patient:sample, cov(unstructured)

margins sample, plot(ytitle(CI_COUPLED (mean, 95% CI)) ytitle(, size(large)) xtitle(, size(large)) xlabel(, labsize(large)) xmtick(-0.25 0 1 1.25) title(Linear mixed effect model))

Komory mají v průměru o 110 vyšší hodnotu CI_COUPLED než síně (p<0.001)

## complex II

graph box ciicoupled, over(sample, label(labsize(large))) ytitle(, size(large))

tabstat ciicoupled, by(sample) stat(n mean sd p25 p50 p75 min max) columns(statistics) format(%9.4g)

mixed ciicoupled i.sample|| patient:sample, cov(unstructured)

margins sample, plot(ytitle(CIICOUPLED (mean, 95% CI)) ytitle(, size(large)) xtitle(, size(large)) xlabel(, labsize(large)) xmtick(-0.25 0 1 1.25) title(Linear mixed effect model))

Komory mají v průměru o 78.5 vyšší hodnotu CII COUPLED než síně (p<0.001)

## proton leak %

graph box leak_perc, over(sample, label(labsize(large))) ytitle(, size(large))

tabstat leak_perc, by(sample) stat(n mean sd p25 p50 p75 min max) columns(statistics) format(%9.4g)

mixed leak_perc i.sample|| patient:sample, cov(unstructured)

margins sample, plot(ytitle(LEAK_PERC (mean, 95% CI)) ytitle(, size(large)) xtitle(, size(large)) xlabel(, labsize(large)) xmtick(-0.25 0 1 1.25) title(Linear mixed effect model))

Rozdíl v procent. Proton leaku není mezi síněmi a komorami signifikantní (p=0.794)

## respiratory control ratio (RCR) = P/L

graph box rcr, over(sample, label(labsize(large))) ytitle(, size(large))

tabstat rcr, by(sample) stat(n mean sd p25 p50 p75 min max) columns(statistics) format(%9.4g)

mixed rcr i.sample|| patient:sample, cov(unstructured)

margins sample, plot(ytitle(RCR (mean, 95% CI)) ytitle(, size(large)) xtitle(, size(large)) xlabel(, labsize(large)) xmtick(-0.25 0 1 1.25) title(Linear mixed effect model))

Není signifikantní rozdíl mezi síněmi a komorami (p=0.258)

## Log(respiratory control ratio (RCR) = P/L) – logaritmicky transformovaný parametr

Data jsou lognormálně rozložená, model tedy vytvořím na logaritmcky transformovaných datech.

gen log_rcr = log(rcr)

label variable log_rcr "Log(RCR)"

graph box log_rcr, over(sample, label(labsize(large))) ytitle(, size(large))

tabstat log_rcr, by(sample) stat(n mean sd p25 p50 p75 min max) columns(statistics) format(%9.4g)

mixed log_rcr i.sample|| patient:sample, cov(unstructured)

margins sample, plot(ytitle(RCR (mean, 95% CI)) ytitle(, size(large)) xtitle(, size(large)) xlabel(, labsize(large)) xmtick(-0.25 0 1 1.25) title(Linear mixed effect model))

Není signifikantní rozdíl mezi síněmi a komorami (p=0.429)

## ET capacity corr/CS - opraveno

graph box etc_cs, over(sample, label(labsize(large))) ytitle(, size(large))

tabstat etc_cs, by(sample) stat(n mean sd p25 p50 p75 min max) columns(statistics) format(%9.4g)

mixed etc_cs i.sample|| patient:sample, cov(unstructured)

margins sample, plot(ytitle(ETC_CS (mean, 95% CI)) ytitle(, size(large)) xtitle(, size(large)) xlabel(, labsize(large)) xmtick(-0.25 0 1 1.25) title(Linear mixed effect model))

Komory mají v průměru o 1407 vyšší hodnotu ETC/CS než síně (p=0.005)

## ET capacity corr/baseline

graph box etc_baseline, over(sample, label(labsize(large))) ytitle(, size(large))

tabstat etc_baseline, by(sample) stat(n mean sd p25 p50 p75 min max) columns(statistics) format(%9.4g)

mixed etc_baseline i.sample|| patient:sample, cov(unstructured)

margins sample, plot(ytitle(ETC_BASELINE (mean, 95% CI)) ytitle(, size(large)) xtitle(, size(large)) xlabel(, labsize(large)) xmtick(-0.25 0 1 1.25) title(Linear mixed effect model))

Není signifikantní rozdíl mezi síněmi a komorami (p=0.697)

## OXPHOS/CS

graph box oxphos_cs, over(sample, label(labsize(large))) ytitle(, size(large))

tabstat oxphos_cs, by(sample) stat(n mean sd p25 p50 p75 min max) columns(statistics) format(%9.4g)

mixed oxphos_cs i.sample|| patient:sample, cov(unstructured)

margins sample, plot(ytitle(OXPHOS_CS (mean, 95% CI)) ytitle(, size(large)) xtitle(, size(large)) xlabel(, labsize(large)) xmtick(-0.25 0 1 1.25) title(Linear mixed effect model))

Komory mají v průměru o 1347 více OXPHOS/CS než síně (p<0.001)

## OXPHOS/BASELINE

graph box oxphos_baseline, over(sample, label(labsize(large))) ytitle(, size(large))

tabstat oxphos_baseline, by(sample) stat(n mean sd p25 p50 p75 min max) columns(statistics) format(%9.4g)

mixed oxphos_baseline i.sample|| patient:sample, cov(unstructured)

margins sample, plot(ytitle(OXPHOS_BASELINE (mean, 95% CI)) ytitle(, size(large)) xtitle(, size(large)) xlabel(, labsize(large)) xmtick(-0.25 0 1 1.25) title(Linear mixed effect model))

Není signifikantní rozdíl v OXPHOS/BASELINE mezi síněmi a komorami (p=0.828)

## 3p/3u

graph box p_3u, over(sample, label(labsize(large))) ytitle(, size(large))

tabstat p_3u, by(sample) stat(n mean sd p25 p50 p75 min max) columns(statistics) format(%9.4g)

mixed p_3u i.sample|| patient:sample, cov(unstructured)

margins sample, plot(ytitle(P_3U (mean, 95% CI)) ytitle(3p/3u, size(large)) xtitle(, size(large)) xlabel(, labsize(large)) xmtick(-0.25 0 1 1.25) title(Linear mixed effect model))

Není signifikantní rozdíl v 3p/3u mezi síněmi a komorami (p=0.828)

## Complex II respiration [%]

graph box cii_perc, over(sample, label(labsize(large))) ytitle(, size(large))

tabstat cii_perc, by(sample) stat(n mean sd p25 p50 p75 min max) columns(statistics) format(%9.4g)

mixed cii_perc i.sample|| patient:sample, cov(unstructured)

margins sample, plot(ytitle(CII_PERC (mean, 95% CI)) ytitle(, size(large)) xtitle(, size(large)) xlabel(, labsize(large)) xmtick(-0.25 0 1 1.25) title(Linear mixed effect model))

Není signifikantní rozdíl v Complex II respiration [%] mezi síněmi a komorami (p=0.503)

## Complex I respiration [%]

graph box ci_perc, over(sample, label(labsize(large))) ytitle(, size(large))

tabstat ci_perc, by(sample) stat(n mean sd p25 p50 p75 min max) columns(statistics) format(%9.4g)

mixed ci_perc i.sample|| patient:sample, ~~cov(unstructured)~~

margins sample, plot(ytitle(CI_PERC (mean, 95% CI)) ytitle(, size(large)) xtitle(, size(large)) xlabel(, labsize(large)) xmtick(-0.25 0 1 1.25) title(Linear mixed effect model))

Není signifikantní rozdíl v Complex I respiration [%] mezi síněmi a komorami (p=0.493)

## 3p/CS n=9

graph box p_cs, over(sample, label(labsize(large))) ytitle(, size(large))

tabstat p_cs, by(sample) stat(n mean sd p25 p50 p75 min max) columns(statistics) format(%9.4g)

mixed p_cs i.sample|| patient:sample, cov(unstructured)

margins sample, plot(ytitle(P_CS (mean, 95% CI)) ytitle(3p/CS, size(large)) xtitle(, size(large)) xlabel(, labsize(large)) xmtick(-0.25 0 1 1.25) title(Linear mixed effect model))

Komory mají signifikantně vyšší hodnotu 3p/CS o 1347 než síně (p<0.001)

## 3u/CS n=9

graph box u_cs, over(sample, label(labsize(large))) ytitle(, size(large))

tabstat u_cs, by(sample) stat(n mean sd p25 p50 p75 min max) columns(statistics) format(%9.4g)

mixed u_cs i.sample|| patient:sample, cov(unstructured)

margins sample, plot(ytitle(U_CS (mean, 95% CI)) ytitle(3u/CS, size(large)) xtitle(, size(large)) xlabel(, labsize(large)) xmtick(-0.25 0 1 1.25) title(Linear mixed effect model))

Komory mají signifikantně vyšší hodnotu 3u/CS o 1347 než síně (p=0.005)

## 3p/CS n=5 (patient 5-9)

tabstat p_cs if patient>4, by(sample) stat(n mean sd p25 p50 p75 min max) columns(statistics) format(%9.4g)

graph box p_cs if patient>4, over(sample, label(labsize(large))) ytitle(, size(large)) title("Patient 5-9")

mixed p_cs i.sample if patient>4|| patient:sample, cov(unstructured)

margins sample, plot(ytitle(P_CS (mean, 95% CI)) ytitle(3p/CS, size(large)) xtitle(, size(large)) xlabel(, labsize(large)) xmtick(-0.25 0 1 1.25) title(Linear mixed effect model (patien 5-9)))

ttest pcsa == pcsv

Komory mají signifikantně vyšší hodnotu 3p/CS o 1693 než síně (p=0.01)

## 3u/CS n=9 (patient 5-9)

tabstat u_cs if patient>4, by(sample) stat(n mean sd p25 p50 p75 min max) columns(statistics) format(%9.4g)

graph box u_cs if patient>4, over(sample, label(labsize(large))) ytitle(, size(large)) title("Patient 5-9")

mixed u_cs i.sample if patient>4|| patient:sample, cov(unstructured)

margins sample, plot(ytitle(U_CS (mean, 95% CI)) ytitle(3u/CS, size(large)) xtitle(, size(large)) xlabel(, labsize(large)) xmtick(-0.25 0 1 1.25) title(Linear mixed effect model (patient 5-9)))

ttest ucsa == ucsv

Komory mají hraničně nesignifikantně vyšší hodnotu 3u/CS o 772 než síně (p=0.22)

## Mal+glut/CS n=5 (patient 5-9)

ttest mal_glut_cs_v == mal_glut_cs_a

## ADP/CS (n=5)

ttest adp_cs_v == adp_cs_a

## Cyt C/CS (n=5)

ttest cyt_c_cs_v == cyt_c_cs_a

## Succ/CS (n=5)

ttest suc_cs_v == suc_cs_a

## Oligo/CS (n=5)

ttest oligo_cs_v == oligo_cs_a

## FCCP/CS (n=5)

ttest fccp_cs_v == fccp_cs_a

## Rot/CS (n=5)

ttest rot_cs_v == rot_cs_a

## AA (n=4)

ttest aa_cs_v == aa_cs_a

## KCN (n=4)

ttest kcn_cs_v == kcn_cs_a

# Analýza variability (reliability)

## Intraclass correlation coefficent

<https://en.wikipedia.org/wiki/Intraclass_correlation>

The three models are:

- One-way random effects: each subject is measured by a different set of k randomly selected raters;
- Two-way random: k raters are randomly selected, then, each subject is measured by the same set of k raters;
- Two-way mixed: k fixed raters are defined. Each subject is measured by the k raters.

Number of measurements:

- Single measures: even though more than one measure is taken in the experiment, reliability is applied to a context where a single measure of a single rater will be performed;
- Average measures: the reliability is applied to a context where measures of k raters will be averaged for each subject.

Consistency or absolute agreement:

- Absolute agreement: the agreement between two raters is of interest, including systematic errors of both raters and random residual errors;
- Consistency: in the context of repeated measurements by the same rater, systematic errors of the rater are canceled and only the random residual error is kept.

Stata: Two-way mixed, single measures, absolute agreement

McGraw and Wong (1996a): Two-way mixed, single score ICC(A,1)

Stata: Two-way mixed, single measures, consistency

McGraw and Wong (1996a): Two-way mixed, single score ICC(C,1)

McGraw, K. O., and S. P. Wong. 1996a. Forming inferences about some intraclass correlation coefficients. Psychological Methods 1: 30–46.

Shrout and Fleiss (1979): undefined

*~~Shrout, P. E., and J. L. Fleiss. 1979. Intraclass correlations: Uses in assessing rater reliability. Psychological Bulletin 86: 420–428.~~*

Hodnocení reliability:

Dle Koo and Li (2016):

- below 0.50: poor
- between 0.50 and 0.75: moderate
- between 0.75 and 0.90: good
- above 0.90: excellent

Koo TK, Li MY (June 2016). "A Guideline of Selecting and Reporting Intraclass Correlation Coefficients for Reliability Research". Journal of Chiropractic Medicine. 15 (2): 155–63. doi:10.1016/j.jcm.2016.02.012. PMC 4913118. PMID 27330520.


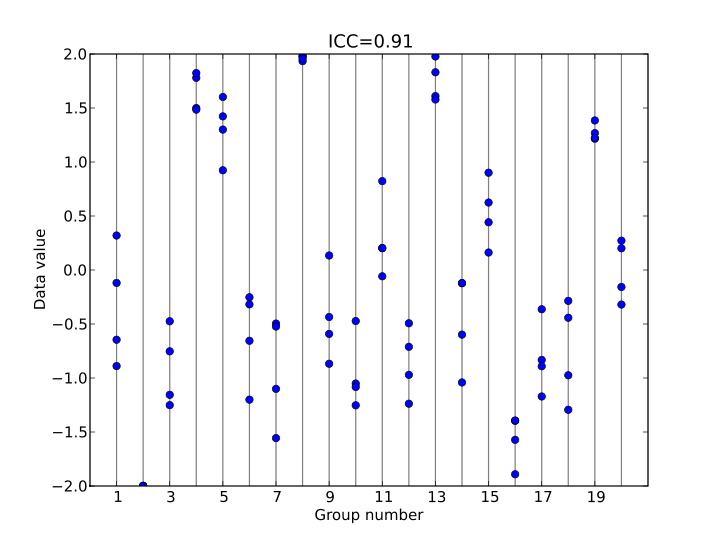


A dot plot showing a dataset with high intraclass correlation. Values from the same group tend to be similar.


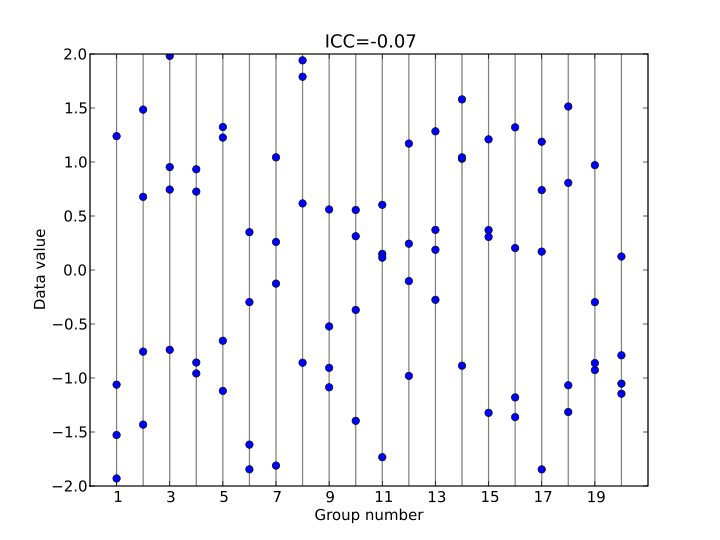


A dot plot showing a dataset with low intraclass correlation. There is no tendency for values from the same group to be similar.


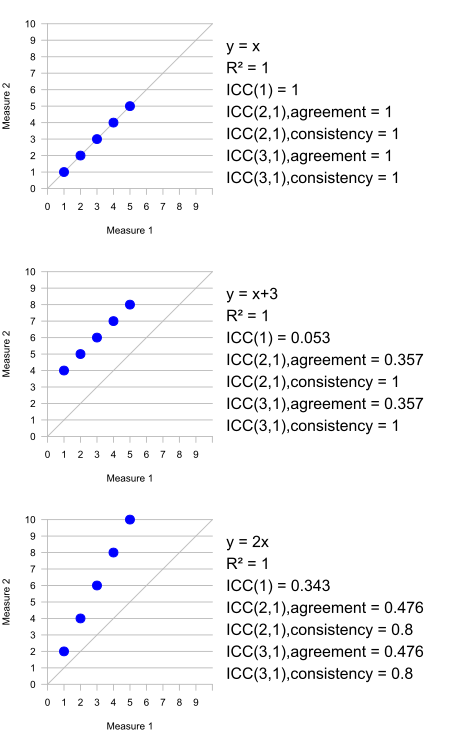


R^2^ – říká jestli body (u nás např. závislost síní na komorách) leží na přímce bez ohledu na její pozici a sklon, R^2^ = 1 leží přesně na přímce (funkční závislostú, R^2^=0 neleží na žadné přímce, mrak dat

ICC agreement – leží data na diagonále, např. u nás síně = komory

ICC consistenci – leží data na přímce, která je paralelní s diagonálou?

## respiratory control ratio (RCR) = P/L

### A-V

#### ICC agreement

twoway (scatter rcr patient if sample ==0) (scatter rcr patient if sample == 1), ytitle(, size(medlarge)) ylabel(0(1)6) xtitle(, size(medlarge)) xlabel(1(1)9, grid) legend(order(1 "Atrium" 2 "Ventricle") size(medlarge))

batplot rcr0 rcr1

icc rcr measurement sample, mixed abs

ICC = 0.16

#### ICC consistency

icc rcr measurement sample, cons mixed

ICC = 0.16

#### R^2^

twoway (scatter rcr0 rcr1) (scatteri 0 0 6 6, recast(line)), ytitle(Atrium) ytitle(, size(large)) ylabel(0(1)6) xtitle(Ventricle) xtitle(, size(large)) xlabel(0(1)6) legend(off) xsize(4) ysize(4) aspectratio(1)

regress rcr i.sample

### A-A

#### ICC agreement

twoway (scatter rcr patient if sample ==0) (scatter rcr patient if sample == 1) if patient <5 & sample ==0, ytitle(, size(medlarge)) ylabel(0(1)6) xtitle(, size(medlarge)) xlabel(1(1)4, grid) legend(order(1 "Atrium" ) size(medlarge))

icc rcr patient sample_a if sample_a!=3, mixed abs

ICC = 0.78

twoway (scatter rcr patient if sample ==0) (scatter rcr patient if sample == 1) if patient <5 & sample ==1, ytitle(, size(medlarge)) ylabel(0(1)6) xtitle(, size(medlarge)) xlabel(1(1)4, grid) legend(order(2 "Ventricle") size(medlarge))

#### ICC consistency

icc rcr patient sample_a if sample_a!=3, cons mixed

ICC = 0.73

#### R^2^

twoway (scatter rcr0 rcr1) (scatteri 0 0 6 6, recast(line)), ytitle(Atrium) ytitle(, size(large)) ylabel(0(1)6) xtitle(Ventricle) xtitle(, size(large)) xlabel(0(1)6) legend(off) xsize(4) ysize(4) aspectratio(1)

regress rcr i.sample_a

### V-V

#### ICC agreement

icc rcr patient sample_v, mixed abs

ICC = 0.74

#### ICC consistency

icc rcr patient sample_v, cons mixed

ICC = 0.66

#### R^2^

twoway (scatter rcr0 rcr1) (scatteri 0 0 6 6, recast(line)), ytitle(Atrium) ytitle(, size(large)) ylabel(0(1)6) xtitle(Ventricle) xtitle(, size(large)) xlabel(0(1)6) legend(off) xsize(4) ysize(4) aspectratio(1)

regress rcr i.sample_v

## 3p/3u

### A-V

#### ICC agreement

twoway (scatter p_3u patient if sample ==0) (scatter p_3u patient if sample == 1), ytitle(, size(medlarge)) ylabel(0(.5)2) xtitle(, size(medlarge)) xlabel(1(1)9, grid) legend(order(1 "Atrium" 2 "Ventricle") size(medlarge))

batplot p_3u0 p_3u1

icc p_3u measurement sample, míxed abs

ICC = 0.08

#### ICC consistency

icc p_3u measurement sample, cons mixed

ICC = 0.07

#### R^2^

twoway (scatter p_3u0 p_3u1) (scatteri 0 0 2 2, recast(line)), ytitle(Atrium) ytitle(, size(large)) ylabel(0(.25)2) xtitle(Ventricle) xtitle(, size(large)) xlabel(0(.25)2) legend(off) xsize(4) ysize(4) aspectratio(1)

regress p_3u i.sample

### A-A

#### ICC agreement

twoway (scatter p_3u patient if sample ==0) (scatter p_3u patient if sample == 1) if patient <5 & sample ==0, ytitle(, size(medlarge)) ylabel(0(.5)2) xtitle(, size(medlarge)) xlabel(1(1)4, grid) legend(order(1 "Atrium" ) size(medlarge))

icc p_3u patient sample_a if sample_a!=3, mixed abs

ICC = 0.89

#### ICC consistency

icc p_3u patient sample_a if sample_a!=3, cons mixed

ICC = 0.86

#### R^2^

twoway (scatter rcr0 rcr1) (scatteri 0 0 6 6, recast(line)), ytitle(Atrium) ytitle(, size(large)) ylabel(0(1)6) xtitle(Ventricle) xtitle(, size(large)) xlabel(0(1)6) legend(off) xsize(4) ysize(4) aspectratio(1)

regress p_3u i.sample_a

### V-V

#### ICC agreement

twoway (scatter p_3u patient if sample ==0) (scatter p_3u patient if sample == 1) if patient <5 & sample ==1, ytitle(, size(medlarge)) ylabel(0(.5)2) xtitle(, size(medlarge)) xlabel(1(1)4, grid) legend(order(2 "Ventricle") size(medlarge))

icc p_3u patient sample_v if sample_v!=3

ICC = 0.89

#### ICC consistency

icc p_3u patient sample_v if sample_v!=3,cons mixed

ICC = 0.87

#### R^2^

twoway (scatter rcr0 rcr1) (scatteri 0 0 6 6, recast(line)), ytitle(Atrium) ytitle(, size(large)) ylabel(0(1)6) xtitle(Ventricle) xtitle(, size(large)) xlabel(0(1)6) legend(off) xsize(4) ysize(4) aspectratio(1)

regress p_3u i.sample_v

## Complex II respiration [%]

### A-V

#### ICC agreement

twoway (scatter cii_perc patient if sample ==0) (scatter cii_perc patient if sample == 1), ytitle(, size(medlarge)) ylabel(0(.1).7) xtitle(, size(medlarge)) xlabel(1(1)9, grid) legend(order(1 "Atrium" 2 "Ventricle") size(medlarge))

batplot cii_perc0 cii_perc1

icc measurement sample, mixed abs

ICC = 0.60

#### ICC consistency

icc cii_perc measurement sample,cons mixed

ICC = 0.16

#### R^2^

twoway (scatter rcr0 rcr1) (scatteri 0 0 6 6, recast(line)), ytitle(Atrium) ytitle(, size(large)) ylabel(0(1)6) xtitle(Ventricle) xtitle(, size(large)) xlabel(0(1)6) legend(off) xsize(4) ysize(4) aspectratio(1)

regress cii_perc i.sample

### A-A

#### ICC consistency

twoway (scatter cii_perc patient if sample ==0) (scatter cii_perc patient if sample == 1) if patient <5 & sample ==0, ytitle(, size(medlarge)) ylabel(0(.1).7) xtitle(, size(medlarge)) xlabel(1(1)4, grid) legend(order(1 "Atrium" ) size(medlarge))

icc cii_perc patient sample_a if sample_a!=3, mixed abs

ICC = 0.70

#### ICC consistency

icc cii_perc patient sample_a if sample_a!=3, cons mixed

ICC = 0.16

#### R^2^

twoway (scatter rcr0 rcr1) (scatteri 0 0 6 6, recast(line)), ytitle(Atrium) ytitle(, size(large)) ylabel(0(1)6) xtitle(Ventricle) xtitle(, size(large)) xlabel(0(1)6) legend(off) xsize(4) ysize(4) aspectratio(1)

regress cii_perc i.sample_a

### V-V

#### ICC agreement

twoway (scatter cii_perc patient if sample ==0) (scatter cii_perc patient if sample == 1) if patient <5 & sample ==1, ytitle(, size(medlarge)) ylabel(0(.1).7) xtitle(, size(medlarge)) xlabel(1(1)4, grid) legend(order(2 "Ventricle") size(medlarge))

icc cii_perc patient sample_v, mixed abs

ICC = 0.70

#### ICC consistency

icc cii_perc patient sample_v, cons mixed

ICC = 0.73

#### R^2^

twoway (scatter rcr0 rcr1) (scatteri 0 0 6 6, recast(line)), ytitle(Atrium) ytitle(, size(large)) ylabel(0(1)6) xtitle(Ventricle) xtitle(, size(large)) xlabel(0(1)6) legend(off) xsize(4) ysize(4) aspectratio(1)

regress cii_perc i.sample_v

## Complex I respiration [%]

### A-V

#### ICC agreement

twoway (scatter ci_perc patient if sample ==0) (scatter ci_perc patient if sample == 1), ytitle(, size(medlarge)) ylabel(0(1)6) xtitle(, size(medlarge)) xlabel(1(1)9, grid) legend(order(1 "Atrium" 2 "Ventricle") size(medlarge))

batplot ci_perc0 ci_perc1

icc ci_perc measurement sample, mixed abs

ICC = 0.21

#### ICC consistency

icc ci_perc measurement sample, cons mixed

ICC = 0.19

#### R^2^

twoway (scatter rcr0 rcr1) (scatteri 0 0 6 6, recast(line)), ytitle(Atrium) ytitle(, size(large)) ylabel(0(1)6) xtitle(Ventricle) xtitle(, size(large)) xlabel(0(1)6) legend(off) xsize(4) ysize(4) aspectratio(1)

regress ci_perc i.sample

### A-A

icc ci_perc patient sample_a if sample_a!=3, mixed

ICC = 0.78

### V-V

icc ci_perc patient sample_v

ICC = 0.74

## 3p/CS

### A-V

#### ICC agreement

twoway (scatter p_cs patient if sample ==0) (scatter p_cs patient if sample == 1), ytitle(, size(medlarge)) ylabel(0(1)6) xtitle(, size(medlarge)) xlabel(1(1)9, grid) legend(order(1 "Atrium" 2 "Ventricle") size(medlarge))

batplot p_cs0 p_cs1

icc p_cs measurement sample, mixed abs

ICC = 0.51

#### ICC consistency

icc p_cs measurement sample, cons mixed

ICC = 0.66

#### R^2^

twoway (scatter p_cs0 p_cs1) (scatteri 0 0 15000 15000, recast(line)), ytitle(Atrium) ytitle(, size(large)) ylabel(0(2500)15000) xtitle(Ventricle) xtitle(, size(large)) xlabel(0(2500)15000) legend(off) xsize(4) ysize(4) aspectratio(1)

regress p_cs i.sample

## 3u/CS

### A-V

#### ICC agreement

twoway (scatter u_cs patient if sample ==0) (scatter u_cs patient if sample == 1), ytitle(, size(medlarge)) ylabel(0(1)6) xtitle(, size(medlarge)) xlabel(1(1)9, grid) legend(order(1 "Atrium" 2 "Ventricle") size(medlarge))

batplot u_cs0 u_cs1

icc u_cs measurement sample, mixed abs

ICC = 0.43

#### ICC consistency

icc u_cs measurement sample,cons mixed

ICC = 0.54

#### R^2^

twoway (scatter u_cs0 u_cs1) (scatteri 0 0 15000 15000, recast(line)), ytitle(Atrium) ytitle(, size(large)) ylabel(0(2500)15000) xtitle(Ventricle) xtitle(, size(large)) xlabel(0(2500)15000) legend(off) xsize(4) ysize(4) aspectratio(1)

regress u_cs i.sample
