# Supplementary material for: High resolution respirometry to assess function of mitochondria in native homogenates of human heart muscle
Source: PLoS One. 2020 Jan 15;15(1):e0226142. doi: 10.1371/journal.pone.0226142 (PMC6961865; doi:10.1371/journal.pone.0226142)
Supplement: S1 Data — (ZIP) [file pone.0226142.s003.zip › Analysis_duplicates_rigth_atrial_appendages.docx]

# Duplikáty analýza

Petr Waldauf

Stata 15.0

21.10.2017

## Baseline = STATE 1

### Deskriptiva

graph box baseline, over(chamber) over(duplicate) ytitle(Baseline OCR [pmol/(s*ml)]) ytitle(, size(medlarge)) note(Duplicates, size(medlarge) position(6)) legend(title(Dupicates))

#### Duplikát 1 vs 2 (chamber A a B dohromady)

tabstat baseline , by ( duplicate ) stat( N mean sd p25 median p75 min max) format(%9.3g) col(statistics)

#### Chamber A vs B (duplikát 1 a 2 dohromady)

tabstat baseline , by ( chamber ) stat( N mean sd p25 median p75 min max) format(%9.3g) col(statistics)

## after Mal

### Deskriptiva

graph box after_mal, over(chamber) over(duplicate) ytitle(After Mal OCR [pmol/(s*ml)]) ytitle(, size(medlarge)) note(Duplicates, size(medlarge) position(6)) legend(title(Dupicates))

#### Duplikát 1 vs 2 (chamber A a B dohromady)

tabstat after_mal , by ( duplicate ) stat( N mean sd p25 median p75 min max) format(%9.3g) col(statistics)

#### Chamber A vs B (duplikát 1 a 2 dohromady)

tabstat after_mal , by ( chamber ) stat( N mean sd p25 median p75 min max) format(%9.3g) col(statistics)

## after Glut

### Deskriptiva

graph box after_glut, over(chamber) over(duplicate) ytitle(After glut OCR [pmol/(s*ml)]) ytitle(, size(medlarge)) note(Duplicates, size(medlarge) position(6)) legend(title(Dupicates))

#### Duplikát 1 vs 2 (chamber A a B dohromady)

tabstat after_glut , by ( duplicate ) stat( N mean sd p25 median p75 min max) format(%9.3g) col(statistics)

#### Chamber A vs B (duplikát 1 a 2 dohromady)

tabstat after_glut , by ( chamber ) stat( N mean sd p25 median p75 min max) format(%9.3g) col(statistics)

## after ADP

### Deskriptiva

graph box after_adp, over(chamber) over(duplicate) ytitle(After ADP OCR [pmol/(s*ml)]) ytitle(, size(medlarge)) note(Duplicates, size(medlarge) position(6)) legend(title(Dupicates))

#### Duplikát 1 vs 2 (chamber A a B dohromady)

tabstat after_adp , by ( duplicate ) stat( N mean sd p25 median p75 min max) format(%9.3g) col(statistics)

#### Chamber A vs B (duplikát 1 a 2 dohromady)

tabstat after_adp , by ( chamber ) stat( N mean sd p25 median p75 min max) format(%9.3g) col(statistics)

## after cyt c

### Deskriptiva

graph box after_cytc, over(chamber) over(duplicate) ytitle(After cytc OCR [pmol/(s*ml)]) ytitle(, size(medlarge)) note(Duplicates, size(medlarge) position(6)) legend(title(Dupicates))

#### Duplikát 1 vs 2 (chamber A a B dohromady)

tabstat after_cytc , by ( duplicate ) stat( N mean sd p25 median p75 min max) format(%9.3g) col(statistics)

#### Chamber A vs B (duplikát 1 a 2 dohromady)

tabstat after_cytc , by ( chamber ) stat( N mean sd p25 median p75 min max) format(%9.3g) col(statistics)

## after suc: STATE 3/OXPHOS CAPACITY (P') non corrected for ROX

### Deskriptiva

graph box after_suc, over(chamber) over(duplicate) ytitle(After suc OCR [pmol/(s*ml)]) ytitle(, size(medlarge)) note(Duplicates, size(medlarge) position(6)) legend(title(Dupicates))

#### Duplikát 1 vs 2 (chamber A a B dohromady)

tabstat after_suc , by ( duplicate ) stat( N mean sd p25 median p75 min max) format(%9.3g) col(statistics)

#### Chamber A vs B (duplikát 1 a 2 dohromady)

tabstat after_suc , by ( chamber ) stat( N mean sd p25 median p75 min max) format(%9.3g) col(statistics)

## after oligo: leak respiration

### Deskriptiva

graph box after_oligo, over(chamber) over(duplicate) ytitle(After oligo OCR [pmol/(s*ml)]) ytitle(, size(medlarge)) note(Duplicates, size(medlarge) position(6)) legend(title(Dupicates))

#### Duplikát 1 vs 2 (chamber A a B dohromady)

tabstat after_oligo , by ( duplicate ) stat( N mean sd p25 median p75 min max) format(%9.3g) col(statistics)

#### Chamber A vs B (duplikát 1 a 2 dohromady)

tabstat after_oligo , by ( chamber ) stat( N mean sd p25 median p75 min max) format(%9.3g) col(statistics)

## after FCCP: ET capacity (E') non-corrected for ROX

### Deskriptiva

graph box after_fccp , over(chamber) over(duplicate) ytitle(After FCCP OCR [pmol/(s*ml)]) ytitle(, size(medlarge)) note(Duplicates, size(medlarge) position(6)) legend(title(Dupicates))

#### Duplikát 1 vs 2 (chamber A a B dohromady)

tabstat after_fccp , by ( duplicate ) stat( N mean sd p25 median p75 min max) format(%9.3g) col(statistics)

#### Chamber A vs B (duplikát 1 a 2 dohromady)

tabstat after_fccp , by ( chamber ) stat( N mean sd p25 median p75 min max) format(%9.3g) col(statistics)

## after AA

### Deskriptiva

graph box after_aa, over(chamber) over(duplicate) ytitle(After AA OCR [pmol/(s*ml)]) ytitle(, size(medlarge)) note(Duplicates, size(medlarge) position(6)) legend(title(Dupicates))

#### Duplikát 1 vs 2 (chamber A a B dohromady)

tabstat after_aa , by ( duplicate ) stat( N mean sd p25 median p75 min max) format(%9.3g) col(statistics)

#### Chamber A vs B (duplikát 1 a 2 dohromady)

tabstat after_aa , by ( chamber ) stat( N mean sd p25 median p75 min max) format(%9.3g) col(statistics)

## complex I corrected for ROX

### Deskriptiva

graph box complex_i_corr, over(chamber) over(duplicate) ytitle(Complex_i_corr OCR [pmol/(s*ml)]) ytitle(, size(medlarge)) note(Duplicates, size(medlarge) position(6)) legend(title(Dupicates))

#### Duplikát 1 vs 2 (chamber A a B dohromady)

tabstat complex_i_corr , by ( duplicate ) stat( N mean sd p25 median p75 min max) format(%9.3g) col(statistics)

#### Chamber A vs B (duplikát 1 a 2 dohromady)

tabstat complex_i_corr , by ( chamber ) stat( N mean sd p25 median p75 min max) format(%9.3g) col(statistics)

## C I control ratio

### Deskriptiva

graph box ci_control_ratio, over(chamber) over(duplicate) ytitle(CI control ratio) ytitle(, size(medlarge)) note(Duplicates, size(medlarge) position(6)) legend(title(Dupicates))

#### Duplikát 1 vs 2 (chamber A a B dohromady)

tabstat ci_control_ratio , by ( duplicate ) stat( N mean sd p25 median p75 min max) format(%9.3g) col(statistics)

#### Chamber A vs B (duplikát 1 a 2 dohromady)

tabstat ci_control_ratio , by ( chamber ) stat( N mean sd p25 median p75 min max) format(%9.3g) col(statistics)

## complex II

### Deskriptiva

graph box complex_ii, over(chamber) over(duplicate) ytitle(Complex_ii OCR [pmol/(s*ml)]) ytitle(, size(medlarge)) note(Duplicates, size(medlarge) position(6)) legend(title(Dupicates))

#### Duplikát 1 vs 2 (chamber A a B dohromady)

tabstat complex_ii , by ( duplicate ) stat( N mean sd p25 median p75 min max) format(%9.3g) col(statistics)

#### Chamber A vs B (duplikát 1 a 2 dohromady)

tabstat complex_ii , by ( chamber ) stat( N mean sd p25 median p75 min max) format(%9.3g) col(statistics)

## CII control ratio

### Deskriptiva

graph box cii_control_ratio, over(chamber) over(duplicate) ytitle(CII control ratio) ytitle(, size(medlarge)) note(Duplicates, size(medlarge) position(6)) legend(title(Dupicates))

#### Duplikát 1 vs 2 (chamber A a B dohromady)

tabstat cii_control_ratio , by ( duplicate ) stat( N mean sd p25 median p75 min max) format(%9.3g) col(statistics)

#### Chamber A vs B (duplikát 1 a 2 dohromady)

tabstat cii_control_ratio , by ( chamber ) stat( N mean sd p25 median p75 min max) format(%9.3g) col(statistics)

## proton leak

### Deskriptiva

graph box proton_leak, over(chamber) over(duplicate) ytitle(Proton leak OCR [pmol/(s*ml)]) ytitle(, size(medlarge)) note(Duplicates, size(medlarge) position(6)) legend(title(Dupicates))

#### Duplikát 1 vs 2 (chamber A a B dohromady)

tabstat proton_leak , by ( duplicate ) stat( N mean sd p25 median p75 min max) format(%9.3g) col(statistics)

#### Chamber A vs B (duplikát 1 a 2 dohromady)

tabstat proton_leak , by ( chamber ) stat( N mean sd p25 median p75 min max) format(%9.3g) col(statistics)

## proton leak %

### Deskriptiva

graph box proton_leak_perc, over(chamber) over(duplicate) ytitle(Proton leak %) ytitle(, size(medlarge)) note(Duplicates, size(medlarge) position(6)) legend(title(Dupicates))

#### Duplikát 1 vs 2 (chamber A a B dohromady)

tabstat proton_leak_perc , by ( duplicate ) stat( N mean sd p25 median p75 min max) format(%9.3g) col(statistics)

#### Chamber A vs B (duplikát 1 a 2 dohromady)

tabstat proton_leak_perc , by ( chamber ) stat( N mean sd p25 median p75 min max) format(%9.3g) col(statistics)

## ET capacity (E) corrected for ROX

### Deskriptiva

graph box etc_corr, over(chamber) over(duplicate) ytitle(Etc_corr OCR [pmol/(s*ml)]) ytitle(, size(medlarge)) note(Duplicates, size(medlarge) position(6)) legend(title(Dupicates))

#### Duplikát 1 vs 2 (chamber A a B dohromady)

tabstat etc_corr , by ( duplicate ) stat( N mean sd p25 median p75 min max) format(%9.3g) col(statistics)

#### Chamber A vs B (duplikát 1 a 2 dohromady)

tabstat etc_corr , by ( chamber ) stat( N mean sd p25 median p75 min max) format(%9.3g) col(statistics)

## respiratory control ratio (RCR) = P/L

### Deskriptiva

graph box rcr, over(chamber) over(duplicate) ytitle(RCR) ytitle(, size(medlarge)) note(Duplicates, size(medlarge) position(6)) legend(title(Dupicates))

#### Duplikát 1 vs 2 (chamber A a B dohromady)

tabstat rcr , by ( duplicate ) stat( N mean sd p25 median p75 min max) format(%9.3g) col(statistics)

#### Chamber A vs B (duplikát 1 a 2 dohromady)

tabstat rcr , by ( chamber ) stat( N mean sd p25 median p75 min max) format(%9.3g) col(statistics)

## Coupling control ratio (leak/OXPHOS)

### Deskriptiva

graph box coupling_control_ratio, over(chamber) over(duplicate) ytitle(Coupling control ratio) ytitle(, size(medlarge)) note(Duplicates, size(medlarge) position(6)) legend(title(Dupicates))

#### Duplikát 1 vs 2 (chamber A a B dohromady)

tabstat coupling_control_ratio , by ( duplicate ) stat( N mean sd p25 median p75 min max) format(%9.3g) col(statistics)

#### Chamber A vs B (duplikát 1 a 2 dohromady)

tabstat coupling_control_ratio , by ( chamber ) stat( N mean sd p25 median p75 min max) format(%9.3g) col(statistics)

## Flux control ratio (FCR) = P/E

### Deskriptiva

graph box fcr, over(chamber) over(duplicate) ytitle(FCR) ytitle(, size(medlarge)) note(Duplicates, size(medlarge) position(6)) legend(title(Dupicates))

#### Duplikát 1 vs 2 (chamber A a B dohromady)

tabstat fcr , by ( duplicate ) stat( N mean sd p25 median p75 min max) format(%9.3g) col(statistics)

#### Chamber A vs B (duplikát 1 a 2 dohromady)

tabstat fcr , by ( chamber ) stat( N mean sd p25 median p75 min max) format(%9.3g) col(statistics)

## OXPHOS control ratio

### Deskriptiva

graph box oxphos_control_ratio, over(chamber) over(duplicate) ytitle(Oxphos control ratio) ytitle(, size(medlarge)) note(Duplicates, size(medlarge) position(6)) legend(title(Dupicates))

#### Duplikát 1 vs 2 (chamber A a B dohromady)

tabstat oxphos_control_ratio , by ( duplicate ) stat( N mean sd p25 median p75 min max) format(%9.3g) col(statistics)

#### Chamber A vs B (duplikát 1 a 2 dohromady)

tabstat oxphos_control_ratio , by ( chamber ) stat( N mean sd p25 median p75 min max) format(%9.3g) col(statistics)

## OXPHOS coupling efficiency

### Deskriptiva

graph box oxphos_coupling_efficiency, over(chamber) over(duplicate) ytitle(Oxphos coupling efficiency) ytitle(, size(medlarge)) note(Duplicates, size(medlarge) position(6)) legend(title(Dupicates))

#### Duplikát 1 vs 2 (chamber A a B dohromady)

tabstat oxphos_coupling_efficiency , by ( duplicate ) stat( N mean sd p25 median p75 min max) format(%9.3g) col(statistics)

#### Chamber A vs B (duplikát 1 a 2 dohromady)

tabstat oxphos_coupling_efficiency , by ( chamber ) stat( N mean sd p25 median p75 min max) format(%9.3g) col(statistics)

## ROX (non-mito)

### Deskriptiva

graph box non_mito, over(chamber) over(duplicate) ytitle(Non mito) ytitle(, size(medlarge)) note(Duplicates, size(medlarge) position(6)) legend(title(Dupicates))

#### Duplikát 1 vs 2 (chamber A a B dohromady)

tabstat non_mito , by ( duplicate ) stat( N mean sd p25 median p75 min max) format(%9.3g) col(statistics)

#### Chamber A vs B (duplikát 1 a 2 dohromady)

tabstat non_mito , by ( chamber ) stat( N mean sd p25 median p75 min max) format(%9.3g) col(statistics)
