# Supplementary material for: High resolution respirometry to assess function of mitochondria in native homogenates of human heart muscle
Source: PLoS One. 2020 Jan 15;15(1):e0226142. doi: 10.1371/journal.pone.0226142 (PMC6961865; doi:10.1371/journal.pone.0226142)
Supplement: S1 Data — (ZIP) [file pone.0226142.s003.zip › Analysis_Durability_of_homogenates.docx]

# Durability analysis, data from 10.2018

Petr Waldauf

Stata 15.5

Created: 07.10.2018

Updated: 07.10.2018

# ET capacity (E) corrected for ROX (absolute values)

## Descriptive statistics

### Atrium

tabstat etc if sample == 0,by(time) stat(n mean sd p25 p50 p75 min max)

### Ventricle

tabstat etc if sample == 1,by(time) stat(n mean sd p25 p50 p75 min max)

graph box etc, over(time) ytitle(ET capacity (E) corrected for ROX) by(sample)

## Linear mixed effect model

mixed etc sample##time|| _all: R.time||patient:||sample:

margins time#sample, plot(ytitle(ET capacity (E) corrected for ROX) yline(245.47 429.61, lpattern(dash) lcolor(%50)) xtitle(Time [hrs]) xtitle(, size(large)) title(Linear mixed effect model) legend(order(1 "Atrium" 2 "Ventricle") size(large) title(Sample)))

### Rozdíl oproti baseline

#### Atrium

margins, dydx( time ) at(sample==0)

Signifikantní pokles je od 6 hodiny dále

#### Ventricle

margins, dydx( time ) at(sample==1)

Signifikantní pokles je od 6 hodiny dále

# ET capacity (E) corrected for ROX (relative values)

Hodnoty vydělím průměrnou hodnotou v baseline a to zvlášť pro síně a komory

gen etc_perc = etc/245.47 if sample==0

replace etc_perc = etc/429.61 if sample==1

## Descriptive statistics

### Atrium

tabstat etc_perc if sample == 0,by(time) stat(n mean sd p25 p50 p75 min max)

### Ventricle

tabstat etc_perc if sample == 1,by(time) stat(n mean sd p25 p50 p75 min max)

graph box etc_perc , over(time) ytitle(Relative ET capacity (E) corrected for ROX) by(sample)

## Linear mixed effect model

mixed etc_perc sample##time|| _all: R.time||patient:||sample:

margins time#sample, plot(ytitle(Relative ET capacity (E) corrected for ROX) yline(1, lpattern(dash) lcolor(%50)) xtitle(Time [hrs]) xtitle(, size(large)) title(Linear mixed effect model) legend(order(1 "Atrium" 2 "Ventricle") size(large) title(Sample)))

### Rozdíl oproti baseline

#### Atrium

margins, dydx( time ) at(sample==0)

Signifikantní pokles je od 6 hodiny dále

#### Ventricle

margins, dydx( time ) at(sample==1)

Signifikantní pokles je od 6 hodiny dále

# Proton leak %

## Descriptive statistics

### Atrium

tabstat leak_perc if sample == 0,by(time) stat(n mean sd p25 p50 p75 min max)

### Ventricle

tabstat leak_perc if sample == 1,by(time) stat(n mean sd p25 p50 p75 min max)

graph box leak_perc, over(time) ytitle(ET capacity (E) corrected for ROX) by(sample)

## Linear mixed effect model

mixed leak_perc sample##time|| _all: R.time||patient:||sample:

margins time#sample, plot(ytitle(Proton leak [%]) yline(29.6, lpattern(dash) lcolor(%50)) xtitle(Time [hrs]) xtitle(, size(large)) title(Linear mixed effect model) legend(order(1 "Atrium" 2 "Ventricle") size(large) title(Sample)))

### Rozdíl oproti baseline

#### Atrium

margins, dydx( time ) at(sample==0)

Není signifikantní změna oproti baseline

#### Ventricle

margins, dydx( time ) at(sample==1)

Není signifikantní změna oproti baseline

# Proton leak % (relative values)

Hodnoty vydělím průměrnou hodnotou v baseline a to zvlášť pro síně a komory

gen leak_perc_perc = leak_perc/29.6025 if sample==0

replace leak_perc_perc = leak_perc/29.6425 if sample==1

## Descriptive statistics

### Atrium

tabstat leak_perc_perc if sample == 0,by(time) stat(n mean sd p25 p50 p75 min max)

### Ventricle

tabstat leak_perc_perc if sample == 1,by(time) stat(n mean sd p25 p50 p75 min max)

graph box leak_perc_perc , over(time) ytitle(Relative ET capacity (E) corrected for ROX) by(sample)

## Linear mixed effect model

mixed leak_perc_perc sample##time|| _all: R.time||patient:||sample:

margins time#sample, plot(ytitle(Relative ET capacity (E) corrected for ROX) yline(1, lpattern(dash) lcolor(%50)) xtitle(Time [hrs]) xtitle(, size(large)) title(Linear mixed effect model) legend(order(1 "Atrium" 2 "Ventricle") size(large) title(Sample)))

### Rozdíl oproti baseline

#### Atrium

margins, dydx( time ) at(sample==0)

Není signifikantní změna oproti baseline

#### Ventricle

margins, dydx( time ) at(sample==1)

Není signifikantní změna oproti baseline
