# Supplementary material for: High resolution respirometry to assess function of mitochondria in native homogenates of human heart muscle
Source: PLoS One. 2020 Jan 15;15(1):e0226142. doi: 10.1371/journal.pone.0226142 (PMC6961865; doi:10.1371/journal.pone.0226142)
Supplement: S1 Data — (ZIP) [file pone.0226142.s003.zip › Analysis_Durability_of_muscle.docx]

# Durability Analysis

Petr Waldauf

Stata 14.2 .. 15.1

09.04.2017

Update 7.4.2018

Obsah

[Durability Analysis 1](#_Toc510891305)

[Malate 2](#_Toc510891306)

[Glutamate 2](#_Toc510891307)

[ADP 3](#_Toc510891308)

[Cytochrome C 3](#_Toc510891309)

[Succinate 4](#_Toc510891310)

[FCCP 4](#_Toc510891311)

[Lineární regrese 6](#_Toc510891312)

[Mixed effect model 7](#_Toc510891313)

[FCCP % baseline 8](#_Toc510891314)

[Všichni 3 pacienti 8](#_Toc510891315)

[Pacient 1 8](#_Toc510891316)

[Pacient 2 9](#_Toc510891317)

[Pacient 3 9](#_Toc510891318)

[Oligomycine 9](#_Toc510891319)

[Lineární regrese 10](#_Toc510891320)

[Mixed effect model 11](#_Toc510891321)

[Oligomycine/ADP 13](#_Toc510891322)

[Lineární regrese 14](#_Toc510891323)

[Mixed effect model 14](#_Toc510891324)

[Antimycine 16](#_Toc510891325)

[Lineární regrese 17](#_Toc510891326)

[Mixed effect model 18](#_Toc510891327)

Poznámka:

Péčko je parametr: P>|z|

p25 = 25th percentile

p50 = 50th percentile

p75 = 75th percentile

cv = coefficient of variation (sd/mean)

label define sample 0 "Baseline" 1 "3 hrs" 2 "12 hrs" 3 "24 hrs" 4 "48 hrs" 5 "72 hrs"

label define substrate 0 "Malate" 1 "Glutamate" 2 "ADP" 3 "CC" 4 "Succinate" 5 "Oligo" 6 "FCCP" 7 "Antimycine"

# Malate

tabstat value if substrate ==0, by(sample) stat(N mean sd var cv p25 p50 p75 min max)

graph box value if substrate ==0, over(sample) ytitle(Malate) ytitle(, size(large))

graph box value if substrate ==0, over(order) over(sample) ytitle(Malate) ytitle(, size(large))

# Glutamate

tabstat value if substrate ==1, by(sample) stat(N mean sd var cv p25 p50 p75 min max)

graph box value if substrate ==1, over(sample) ytitle(Glutamate) ytitle(, size(large))

graph box value if substrate ==1, over(order) over(sample) ytitle(Glutamate) ytitle(, size(large))

# ADP

tabstat value if substrate ==2, by(sample) stat(N mean sd var cv p25 p50 p75 min max)

graph box value if substrate ==2, over(sample) ytitle(ADP) ytitle(, size(large))

graph box value if substrate ==2, over(order) over(sample) ytitle(ADP) ytitle(, size(large))

# Cytochrome C

tabstat value if substrate ==3, by(sample) stat(N mean sd var cv p25 p50 p75 min max)

graph box value if substrate ==3, over(sample) ytitle(Cytochrome C) ytitle(, size(large))

graph box value if substrate ==3, over(order) over(sample) ytitle(Cytochrome C) ytitle(, size(large))

# Succinate

tabstat value if substrate ==4, by(sample) stat(N mean sd var cv p25 p50 p75 min max)

graph box value if substrate ==4, over(sample) ytitle(Succinate) ytitle(, size(large))

graph box value if substrate ==4, over(order) over(sample) ytitle(Succinate) ytitle(, size(large))

# FCCP

tabstat value if substrate ==6, by(sample) stat(N mean sd var cv p25 p50 p75 min max)

graph box value if substrate ==6, over(sample) ytitle(FCCP) ytitle(, size(large))

graph box value if substrate ==6, over(order) over(sample) ytitle(FCCP) ytitle(, size(large))

twoway (connected value sample if substrate ==6 & patient ==1, connect(ascending)) (connected value sample if substrate ==6 & patient ==2, connect(ascending)) (connected value sample if substrate ==6 & patient ==3, connect(ascending))

## Lineární regrese

Péčko je parametr: P>|z|

regress value i.sample##i.patient if substrate ==6

margins sample##patient

marginsplot

## Mixed effect model

Péčko je parametr: P>|z|

mixed value i.sample||patient: if substrate ==6

estimates store m1

mixed value i.sample||patient: if substrate ==6,residuals(independent, by( sample ))

estimates store m2

lrtest m1 m2

margins sample

marginsplot

margins, dydx( sample )

marginsplot, title ("Prediction of Difference of RCC" "Against Baseline with 95% CI" ) ytitle( , size(medlarge)) xtitle(Propofol, size(medlarge)) xtitle(Time, size(medlarge)) xlabel( 1 "3 hrs" 2 "12 hrs" 3 "24 hrs" 4 "48 hrs" 5 "72 hrs") yline(0)

## FCCP % baseline

### Všichni 3 pacienti

tabstat valueperc if substrate ==6, by(sample) stat(N mean sd var cv p25 p50 p75 min max)

Ve 48 hod: pokles na 80.9%±21.3%

V 72 hod: pokles na 62.4%±44.1%

twoway (connected valueperc sample if substrate ==6 & patient ==1, connect(ascending)) (connected valueperc sample if substrate ==6 & patient ==2, connect(ascending)) (connected valueperc sample if substrate ==6 & patient ==3, connect(ascending))

### Pacient 1

tabstat valueperc if substrate ==6 & patient ==1, by(sample) stat(N mean sd var cv p25 p50 p75 min max)

Ve 48 hod: vzestup na 107.7%±0.2%

V 72 hod: vzestup na 118.3%±4.3%

### Pacient 2

tabstat valueperc if substrate ==6 & patient ==2, by(sample) stat(N mean sd var cv p25 p50 p75 min max)

Ve 48 hod: pokles na 67.7%±9.1%

V 72 hod: pokles na 41.0%±1.1%

### Pacient 3

tabstat valueperc if substrate ==6 & patient ==3, by(sample) stat(N mean sd var cv p25 p50 p75 min max)

Ve 48 hod: pokles na 67.4%±5.8%

V 72 hod: pokles na 28.1%±13.9%

# Oligomycine

tabstat value if substrate ==5, by(sample) stat(N mean sd var cv p25 p50 p75 min max)

graph box value if substrate ==5, over(sample) ytitle(Oligomycine) ytitle(, size(large))

graph box value if substrate ==5, over(order) over(sample) ytitle(Oligomycine) ytitle(, size(large))

twoway (connected value sample if substrate ==5 & patient ==1, connect(ascending)) (connected value sample if substrate ==5 & patient ==2, connect(ascending)) (connected value sample if substrate ==5 & patient ==3, connect(ascending))

## Lineární regrese

Péčko je parametr: P>|z|

regress value i.sample##i.patient if substrate ==5

margins sample##patient

marginsplot

## Mixed effect model

Péčko je parametr: P>|z|

mixed value i.sample||patient: if substrate ==5

estimates store m1

mixed value i.sample||patient: if substrate ==5 ,residuals(independent, by( sample ))

estimates store m2

lrtest m1 m2

margins sample

marginsplot

margins, dydx( sample )

marginsplot, title ("Prediction of Difference of Leak" "Against Baseline with 95% CI" ) ytitle( , size(medlarge)) xtitle(Propofol, size(medlarge)) xtitle(Time, size(medlarge)) xlabel( 1 "3 hrs" 2 "12 hrs" 3 "24 hrs" 4 "48 hrs" 5 "72 hrs") yline(0)

# Oligomycine/ADP

tabstat value if substrate ==8, by(sample) stat(N mean sd var cv p25 p50 p75 min max)

graph box value if substrate ==8, over(sample) ytitle(Oligomycine/ADP) ytitle(, size(large))

graph box value if substrate ==8, over(order) over(sample) ytitle(Oligomycine/ADP) ytitle(, size(large))

twoway (connected value sample if substrate ==8 & patient ==1, connect(ascending)) (connected value sample if substrate ==8 & patient ==2, connect(ascending)) (connected value sample if substrate ==8 & patient ==3, connect(ascending))

## Lineární regrese

Péčko je parametr: P>|z|

regress value i.sample##i.patient if substrate ==8

margins sample##patient

marginsplot

## Mixed effect model

Péčko je parametr: P>|z|

mixed value i.sample||patient: if substrate ==8

estimates store m1

mixed value i.sample||patient: if substrate ==8, residuals(independent, by( sample ))

estimates store m2

lrtest m1 m2

margins sample

marginsplot

margins, dydx( sample )

marginsplot, title ("Prediction of Difference of Leak/ADP" "Against Baseline with 95% CI" ) ytitle( , size(medlarge)) xtitle(Propofol, size(medlarge)) xtitle(Time, size(medlarge)) xlabel( 1 "3 hrs" 2 "12 hrs" 3 "24 hrs" 4 "48 hrs" 5 "72 hrs") yline(0)

Statisticky signifikantní vyšší je rozdíl leaku/ADP oproti baseline ve 12, 48 a 72 hod – je vyšší,

Ve 12 hod je hr. nesignifikantní (p=0.117)

# Antimycine

tabstat value if substrate ==7, by(sample) stat(N mean sd var cv p25 p50 p75 min max)

graph box value if substrate ==7, over(sample) ytitle(Antimycine) ytitle(, size(large))

graph box value if substrate ==7, over(order) over(sample) ytitle(Antimycine) ytitle(, size(large))

twoway (connected value sample if substrate ==7 & patient ==1, connect(ascending)) (connected value sample if substrate ==7 & patient ==2, connect(ascending)) (connected value sample if substrate ==7 & patient ==3, connect(ascending))

## Lineární regrese

Péčko je parametr: P>|z|

regress value i.sample##i.patient if substrate ==7

margins sample##patient

marginsplot

## Mixed effect model

Péčko je parametr: P>|z|

mixed value i.sample||patient: if substrate ==7

estimates store m1

mixed value i.sample||patient: if substrate ==7,residuals(independent, by( sample ))

estimates store m2

lrtest m1 m2

margins sample

marginsplot

margins, dydx( sample )

marginsplot, title ("Prediction of Difference of Non-Mit" "Against Baseline with 95% CI" ) ytitle( , size(medlarge)) xtitle(Propofol, size(medlarge)) xtitle(Time, size(medlarge)) xlabel( 1 "3 hrs" 2 "12 hrs" 3 "24 hrs" 4 "48 hrs" 5 "72 hrs") yline(0)
