# Supplementary material for: High resolution respirometry to assess function of mitochondria in native homogenates of human heart muscle
Source: PLoS One. 2020 Jan 15;15(1):e0226142. doi: 10.1371/journal.pone.0226142 (PMC6961865; doi:10.1371/journal.pone.0226142)
Supplement: S1 Data — (ZIP) [file pone.0226142.s003.zip › Analysis_Isolated_mitochondria_homogenates_plusCS.docx]

# Analýza homogenátů a izolovaných mitochondrií

Petr Waldauf

Stata 15.1

Vytvořeno: 12.10.2018

Aktualizováno: 30.10.2018

label define sample 0 "Isolated mito"

label define sample 1 "Homogenates", add

label values sample sample

label define duplicates 0 "Sample 1" 1 "Sample 2"

label values duplicate duplicates

# Linear mixed effect model

## Respiratory control ratio (RCR) = P/L

tabstat rcr, by(sample) stat(n mean sd p25 p50 p75 min max) format(%9.2f)

graph box rcr, over(sample, label(labsize(medlarge))) ytitle(, size(medlarge)) subtitle(, size(medlarge))

Pozn.: u izolovaných mitochondrií jsou 2 měření outlierem – ve zdrojových datech označeno jako SAMPLE 1

Isolated mito

tabstat rcr if sample==0, by( duplicate ) stat(n mean sd p25 p50 p75 min max) format(%9.2f)

Homogenates

tabstat rcr if sample==1, by( duplicate ) stat(n mean sd p25 p50 p75 min max) format(%9.2f)

graph box rcr, over(duplicate, label(labsize(medlarge))) ytitle(, size(medlarge)) by(sample) subtitle(, size(medlarge))

mixed rcr sample##duplicate||patient:sample duplicate , cov(unstr)

margins sample, plot(ytitle(RCR (mean, 95% CI)) ytitle(, size(large)) xtitle(, size(large)) xlabel(, labsize(large)) xmtick(-0.25 0 1 1.25) title(Linear mixed effect model))

pwcompare sample, eff

U homogenátů (oba duplikáty dohromady) je hraničně nesignifikantně vyšší hodnota RCR o 0.54 (p=0.097) než u izolovaných mitochondrií (oba duplikáty dohromady).

Za nesignifikanci může outlier (SAMPLE 1)

margins duplicate, over(sample) plot(ytitle(RCR (mean, 95% CI)) ytitle(, size(large)) xtitle(, size(large)) xlabel(, labsize(large)) xmtick(-0.25 0 1 1.25) title(Linear mixed effect model))

Isolated mito

margins duplicate if sample==0, pwcompare(eff)

U izolovaných mitochondrií není signifikantní rozdíl mezi 1. a 2. měřením (p=0.457)

Homogenates

margins duplicate if sample==1, pwcompare(eff)

U homogenátů je signifikantně nižší 2. měření o 0.178 (p=0.038)

## IIIp/CS - updated

tabstat iiipcs, by(sample) stat(n mean sd p25 p50 p75 min max) format(%9.1f)

graph box iiipcs, over(sample, label(labsize(medlarge))) ytitle(, size(medlarge)) subtitle(, size(medlarge))

Isolated mito

tabstat iiipcs if sample==0, by( duplicate ) stat(n mean sd p25 p50 p75 min max) format(%9.1f)

Homogenates

tabstat iiipcs if sample==1, by( duplicate ) stat(n mean sd p25 p50 p75 min max) format(%9.1f)

graph box iiipcs, over(duplicate, label(labsize(medlarge))) ytitle(, size(medlarge)) by(sample) subtitle(, size(medlarge))

mixed iiipcs1 sample##duplicate||patient:sample duplicate , cov(unstr)

~~mixed iiipcs sample##duplicate if iiipcs<20000||patient:sample duplicate , cov(unstr)~~

margins sample, plot(ytitle(IIIp/CS (mean, 95% CI)) ytitle(, size(large)) xtitle(, size(large)) xlabel(, labsize(large)) xmtick(-0.25 0 1 1.25) title(Linear mixed effect model))

pwcompare sample, eff

U homogenátů (oba duplikáty dohromady) je signifikantně nižší hodnota IIIp/CS o 185 (p<0.001) než u izolovaných mitochondrií (oba duplikáty dohromady).

margins duplicate, over(sample) plot(ytitle(IIIPCS (mean, 95% CI)) ytitle(, size(large)) xtitle(, size(large)) xlabel(, labsize(large)) xmtick(-0.25 0 1 1.25) title(Linear mixed effect model))

Isolated mito

margins duplicate if sample==0, pwcompare(eff)

U izolovaných mitochondrií je signifikantní byšší hodnota o 148 při 2. měřením (p<0.001)

Homogenates

margins duplicate if sample==1, pwcompare(eff)

U homogenátů není signifikantní rozdíl mezi 1. a 2. měření (p=0.18).

## IIIu/CS - updated

tabstat iiiucs, by(sample) stat(n mean sd p25 p50 p75 min max) format(%9.1f)

graph box iiiucs, over(sample, label(labsize(medlarge))) ytitle(, size(medlarge)) subtitle(, size(medlarge))

Isolated mito

tabstat iiiucs if sample==0, by( duplicate ) stat(n mean sd p25 p50 p75 min max) format(%9.1f)

Homogenates

tabstat iiiucs if sample==1, by( duplicate ) stat(n mean sd p25 p50 p75 min max) format(%9.1f)

graph box iiiucs, over(duplicate, label(labsize(medlarge))) ytitle(, size(medlarge)) by(sample) subtitle(, size(medlarge))

mixed iiiucs1 sample##duplicate||patient:sample duplicate , cov(unstr)

margins sample, plot(ytitle(IIIu/CS (mean, 95% CI)) ytitle(, size(large)) xtitle(, size(large)) xlabel(, labsize(large)) xmtick(-0.25 0 1 1.25) title(Linear mixed effect model))

pwcompare sample, eff

U homogenátů (oba duplikáty dohromady) je signifikantně nižší hodnota IIIu/CS o 93 (p<0.001) než u izolovaných mitochondrií (oba duplikáty dohromady).

margins duplicate, over(sample) plot(ytitle(IIIUCS (mean, 95% CI)) ytitle(, size(large)) xtitle(, size(large)) xlabel(, labsize(large)) xmtick(-0.25 0 1 1.25) title(Linear mixed effect model))

Isolated mito

margins duplicate if sample==0, pwcompare(eff)

U izolovaných mitochondrií je hraničně nesignifikantně vyšší hodnota u 2. měření o 105 (p=0.066)

Homogenates

margins duplicate if sample==1, pwcompare(eff)

U homogenátů není signifikantní rozdíl mezi 1. a 2. měření (p=0.508)

## % increase with cyt c

tabstat cyt_c_perc, by(sample) stat(n mean sd p25 p50 p75 min max) format(%9.1f)

graph box cyt_c_perc, over(sample, label(labsize(medlarge))) ytitle(, size(medlarge)) subtitle(, size(medlarge))

mixed cyt_c_perc sample##duplicate||patient:sample duplicate , cov(unstr)

margins sample, plot(ytitle(% increase with cyt c (mean, 95% CI)) ytitle(, size(large)) xtitle(, size(large)) xlabel(, labsize(large)) xmtick(-0.25 0 1 1.25) title(Linear mixed effect model))

pwcompare sample, eff

U homogenátů (oba duplikáty dohromady) je signifikantně nižší hodnota % vzestupu po cyt C o 15.5% (p<0.001) než u izolovaných mitochondrií (oba duplikáty dohromady).

## Baseline = STATE 1

tabstat baseline, by(sample) stat(n mean sd p25 p50 p75 min max) format(%9.1f)

graph box baseline, over(sample, label(labsize(medlarge))) ytitle(, size(medlarge)) subtitle(, size(medlarge))

mixed baseline sample##duplicate||patient:sample duplicate , cov(unstr)

margins sample, plot(ytitle(Baseline = STATE 1 (mean, 95% CI)) ytitle(, size(large)) xtitle(, size(large)) xlabel(, labsize(large)) xmtick(-0.25 0 1 1.25) title(Linear mixed effect model))

pwcompare sample, eff

U homogenátů (oba duplikáty dohromady) je signifikantně vyšší hodnota baseline o 5.4 (p=0.003) než u izolovaných mitochondrií (oba duplikáty dohromady).

## CS

tabstat cs, by(sample) stat(n mean sd p25 p50 p75 min max) format(%9.4f)

graph box cs, over(sample, label(labsize(medlarge))) ytitle(, size(medlarge)) subtitle(, size(medlarge))

mixed cs sample##duplicate||patient:sample duplicate , cov(unstr)

margins sample, plot(ytitle(CS (mean, 95% CI)) ytitle(, size(large)) xtitle(, size(large)) xlabel(, labsize(large)) xmtick(-0.25 0 1 1.25) title(Linear mixed effect model))

pwcompare sample, eff

U homogenátů (oba duplikáty dohromady) je signifikantně vyšší hodnota CS o 0.02 (p<0.001) než u izolovaných mitochondrií (oba duplikáty dohromady).

## CS vs Baseline

twoway (scatter cs baseline) (qfitci cs baseline), ytitle(CS) xtitle(Baseline - STATE 1) legend(off)

## IIIp/baseline

tabstat iiipbaseline if iiipbaseline <250, by(sample) stat(n mean sd p25 p50 p75 min max) format(%9.1f)

graph box iiipbaseline if iiipbaseline <250, over(sample, label(labsize(medlarge))) ytitle(, size(medlarge)) subtitle(, size(medlarge))

mixed iiipbaseline sample##duplicate if iiipbaseline <250||patient:sample duplicate , cov(unstr)

margins sample, plot(ytitle(IIIp/baseline (mean, 95% CI)) ytitle(, size(large)) xtitle(, size(large)) xlabel(, labsize(large)) xmtick(-0.25 0 1 1.25) title(Linear mixed effect model))

pwcompare sample, eff

U homogenátů (oba duplikáty dohromady) je nesignifikantně nižší hodnota IIIp/baseline o 50 (p=0.135) než u izolovaných mitochondrií (oba duplikáty dohromady).

## IIIu/baseline

tabstat iiiubaseline if iiipbaseline <250, by(sample) stat(n mean sd p25 p50 p75 min max) format(%9.1f)

graph box iiiubaseline if iiipbaseline <250, over(sample, label(labsize(medlarge))) ytitle(, size(medlarge)) subtitle(, size(medlarge))

mixed iiiubaseline sample##duplicate if iiipbaseline <250||patient:sample duplicate , cov(unstr)

margins sample, plot(ytitle(IIIu/baseline (mean, 95% CI)) ytitle(, size(large)) xtitle(, size(large)) xlabel(, labsize(large)) xmtick(-0.25 0 1 1.25) title(Linear mixed effect model))

pwcompare sample, eff

U homogenátů (oba duplikáty dohromady) je nesignifikantně nižší hodnota IIIu/baseline o 40 (p=0.187) než u izolovaných mitochondrií (oba duplikáty dohromady).
