# Supplementary material for: High resolution respirometry to assess function of mitochondria in native homogenates of human heart muscle
Source: PLoS One. 2020 Jan 15;15(1):e0226142. doi: 10.1371/journal.pone.0226142 (PMC6961865; doi:10.1371/journal.pone.0226142)
Supplement: S1 Data — (ZIP) [file pone.0226142.s003.zip › Analysis_Oxygen_dependency.docx]

# Titrace FCCP – Final Results

Petr Waldauf

Stata 14.2

08.04.2017

Obsah

[Titrace FCCP – Final Results 1](#_Toc479543116)

[Raw data 1](#_Toc479543117)

[Slope 2](#_Toc479543118)

[Mixed-effect models 3](#_Toc479543119)

[Ukázka O2 korekce 3](#_Toc479543120)

[Normalized slope 4](#_Toc479543121)

[Mixed-effect models 5](#_Toc479543122)

[Fractional polynomials 8](#_Toc479543123)

[Jednodušší model 9](#_Toc479543124)

[R^2^ 11](#_Toc479543125)

[O2 slope neg. [pmol/(s*ml)] post CC 11](#_Toc479543126)

[O2 slope neg. [pmol/(s*ml)] post FCCP 12](#_Toc479543127)

[Slope & FCCP correlation 12](#_Toc479543128)

[Slope & CC correlation 12](#_Toc479543129)

# Raw data

| sample | file | slope | R^2^ | normslope | postCC | postFCCP |
| --- | --- | --- | --- | --- | --- | --- |
| 1 | 2017-03-09 P1-01myokard_Km_biopsie_09_03_2017 | 0.448 | 0.998 | 0.145 | 106.6 | 287.5 |
| 2 | 2017-03-14 P1-01MYOKARD_KM_BIOPSIE_14_03_2017 | 0.185 | 0.997 | 0.141 | 51.3 | 130.9 |
| 3 | 2017-03-15 P1-01myokard_Km_biopsie_15_03_2017 | 0.309 | 0.997 | 0.209 | 67.96 | 147.5 |
| 4 | 2017-03-17 P1-01myokard_Km_biopsie_17_03_2017 | 0.16 | 0.995 | 0.182 | 50.5 | 88.1 |
| 5 | 2017-03-23 P1-01myokard_Km_biopsie_23_03_2017 | 0.141 | 0.984 | 0.107 | 74.2 | 132.1 |
| 6 | 2017-03-24 P1-01myokard_Km_biopsie_24_03_2017 | 0.271 | 0.979 | 0.27 | 60.3 | 100.5 |
| 7 | 2017-03-28 P1-01myokard_Km_biopsie_28_3_2017 | 0.464 | 0.996 | 0.387 | 72.5 | 119.8 |

Example of data:

2017-03-09 P1-01myokard_Km_biopsie_09_03_2017

Note:

p25 = 25th percentile

p50 = 50th percentile

p75 = 75th percentile

cv = coefficient of variation (sd/mean)

tabstat slope normslope r2 postcc postfccp , stat(N mean sd var cv p25 p50 p75 min max)

# Slope

tabstat slope, stat(N mean sd var cv p25 p50 p75 min max)

Mean slope: 0.283, SD 0.133

graph box slope

## Mixed-effect models

mixed O2slope O2conc if O2conc > 25 & O2conc < 125 || sample: O2conc, stddev

O2 slope = 93.7 + 0.279*O2conc

## Ukázka O2 korekce

twoway (connected O2conc time, msymbol(point) lcolor(blue)) (connected O2slope time, yaxis(2) msymbol(point) lcolor(red)) (connected O2slopecorr time, yaxis(2) msymbol(point) lcolor(green) lwidth(medium)) (scatteri 0 31.45 200 31.45 "malate 10 μl", recast(line) lcolor(gray) lwidth(vthin) mcolor(gray) msymbol(point)) (scatteri 0 33.8 "glutamate 18 μl" 200 33.8 "glutamate 18 μl", recast(line) lcolor(gray) lwidth(vthin)) (scatteri 0 38.22 "ADP 21 μl" 200 38.22 "ADP 21 μl", recast(line) lcolor(gray) lwidth(vthin)) (scatteri 0 41.01 "Cyt C 10 μl" 200 41.01 "Cyt C 10 μl", recast(line) lcolor(gray) lwidth(vthin)) (scatteri 0 45.13 "succinate 20 μl" 200 45.13 "succinate 20 μl", recast(line) lcolor(gray) lwidth(vthin)) (scatteri 0 48.24 "oligomycine 1 μl" 200 48.24 "oligomycine 48.24", recast(line) lcolor(gray) lwidth(vthin)) (scatteri 0 50.3 200 50.3 "FCCP 2 μl", recast(line) lcolor(gray) lwidth(vthin)) (scatteri 0 52.1 200 52.1 "FCCP 0.25 μl", recast(line) lcolor(gray) lwidth(vthin)) (scatteri 0 53.87 200 53.87 "FCCP 0.25 μl", recast(line) lcolor(gray) lwidth(vthin)) (scatteri 0 55.77 200 55.77 "FCCP 0.25 μl", recast(line) lcolor(gray) lwidth(vthin)) (scatteri 0 57.56 200 57.56 "FCCP 0.25 μL", recast(line) lcolor(gray) lwidth(vthin)) (scatteri 0 59.79 "AA" 200 59.79 "AA 0.4 μl", recast(line) lcolor(gray) lwidth(vthin)) if time >30, ytitle("O2 concentration [μM]" "O2 slope [pmol/(s*ml)]]") ytitle(, size(large)) ylabel(, labsize(medlarge)) xtitle(Time [min]) xtitle(, size(large)) xlabel(, labsize(medlarge)) legend(order(1 "O2 concetration [μM]" 14 "O2 slope[pmol/(s*ml)]" 15 "O2 slope corr[pmol/(s*ml)]" ) cols(1) size(large))

# Normalized slope

tabstat normslope, stat(N mean sd var cv p25 p50 p75 min max)

Mean normalized slope: 0.206, SD 0.1, 0.18 (0.14;0.27)

graph box normslope

twoway (connected normO2slope O2conc if sample ==1, msymbol(point)) (connected normO2slope O2conc if sample ==2, msymbol(point)) (connected normO2slope O2conc if sample ==3, msymbol(point)) (connected normO2slope O2conc if sample ==4, msymbol(point)) (connected normO2slope O2conc if sample ==5, msymbol(point)) (connected normO2slope O2conc if sample ==6, msymbol(point)) (connected normO2slope O2conc if sample ==7, msymbol(point))

## Mixed-effect models

mixed normO2slope O2conc if O2conc > 25 & O2conc < 125 || sample: O2conc, stddev

estimates store m1

margins, at( O2conc = (25(5)125))

marginsplot, recast(line) recastci(rarea)


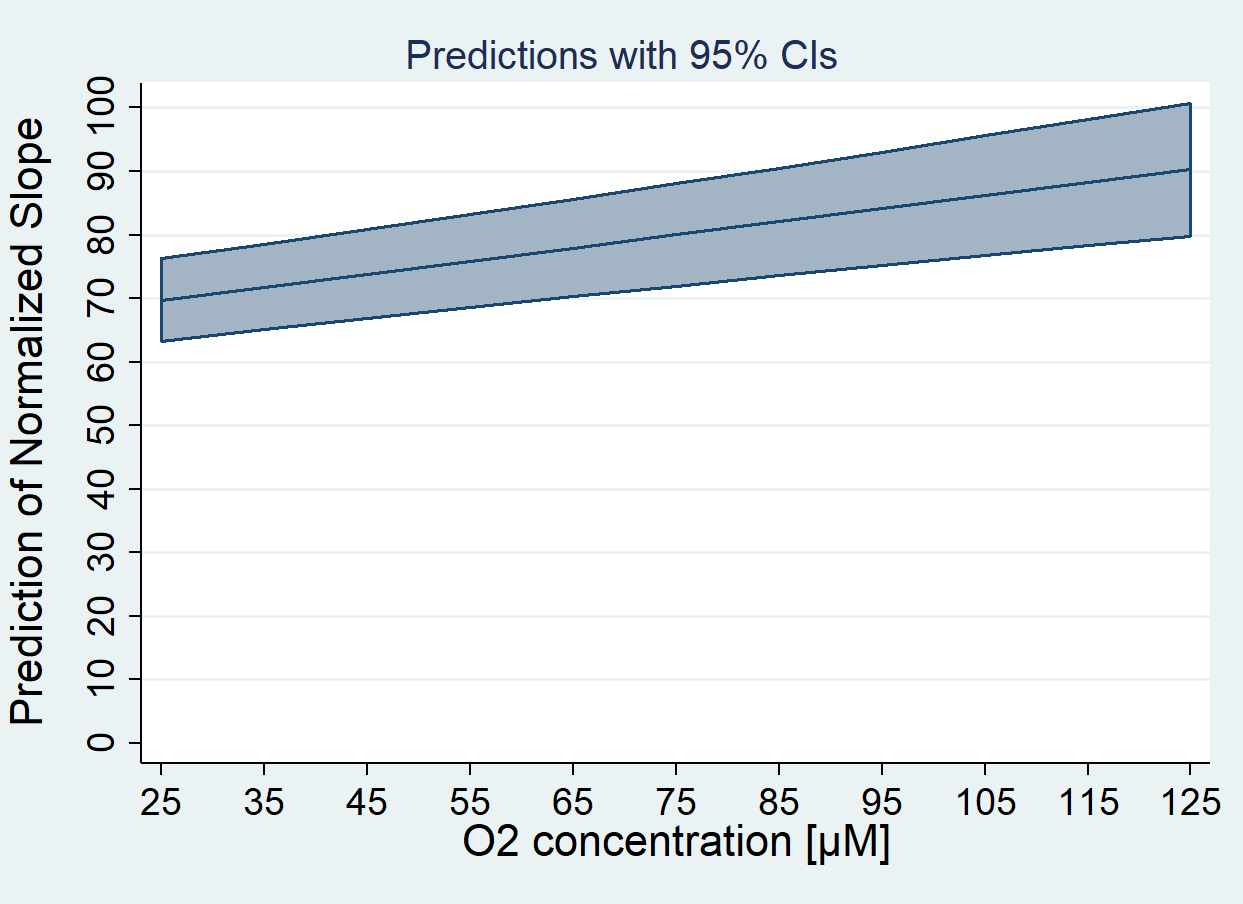


predict wgt_hat, fitted

twoway (connected wgt_hat O2conc if O2conc > 25 & O2conc < 125, msize(vsmall) msymbol(circle) connect(none))


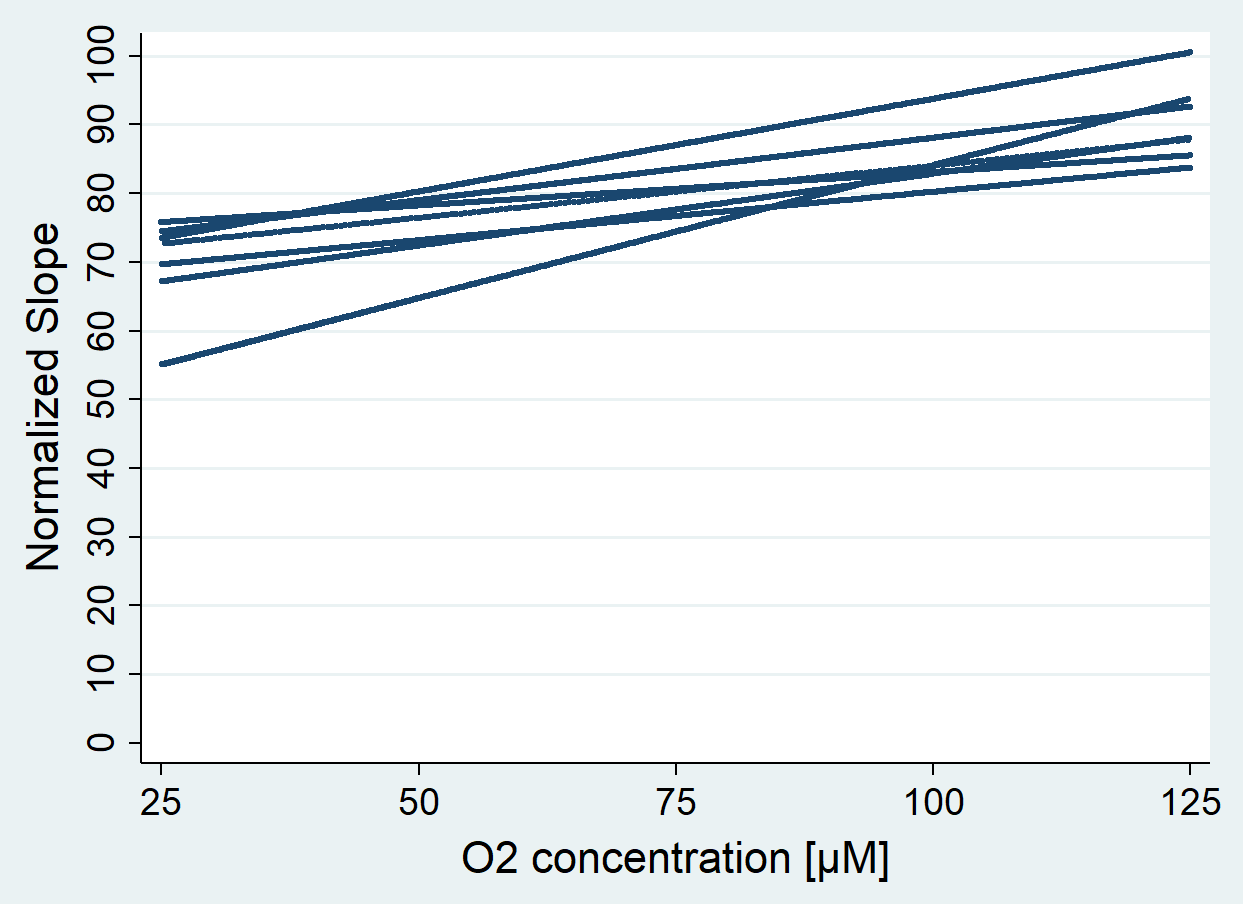


mixed normO2slope O2conc if O2conc > 25 & O2conc < 125 || sample: O2conc, cov(unstr) stddev

estimates store m2

margins, at( O2conc = (25(5)125))

marginsplot, recast(line) recastci(rarea)


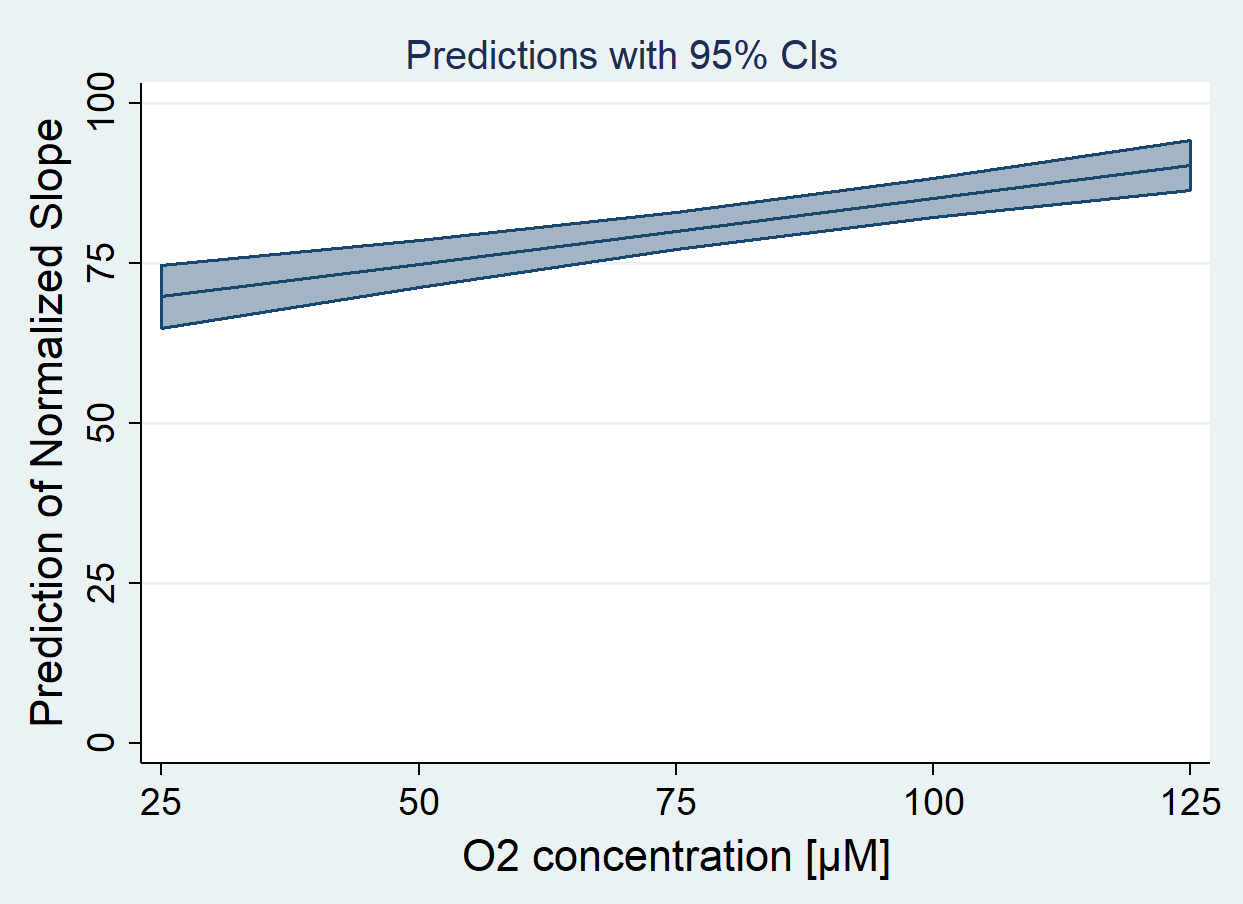


predict wgt_hat1, fitted

twoway (connected wgt_hat1 O2conc if O2conc > 25 & O2conc < 125, msize(vsmall) msymbol(circle) connect(none))


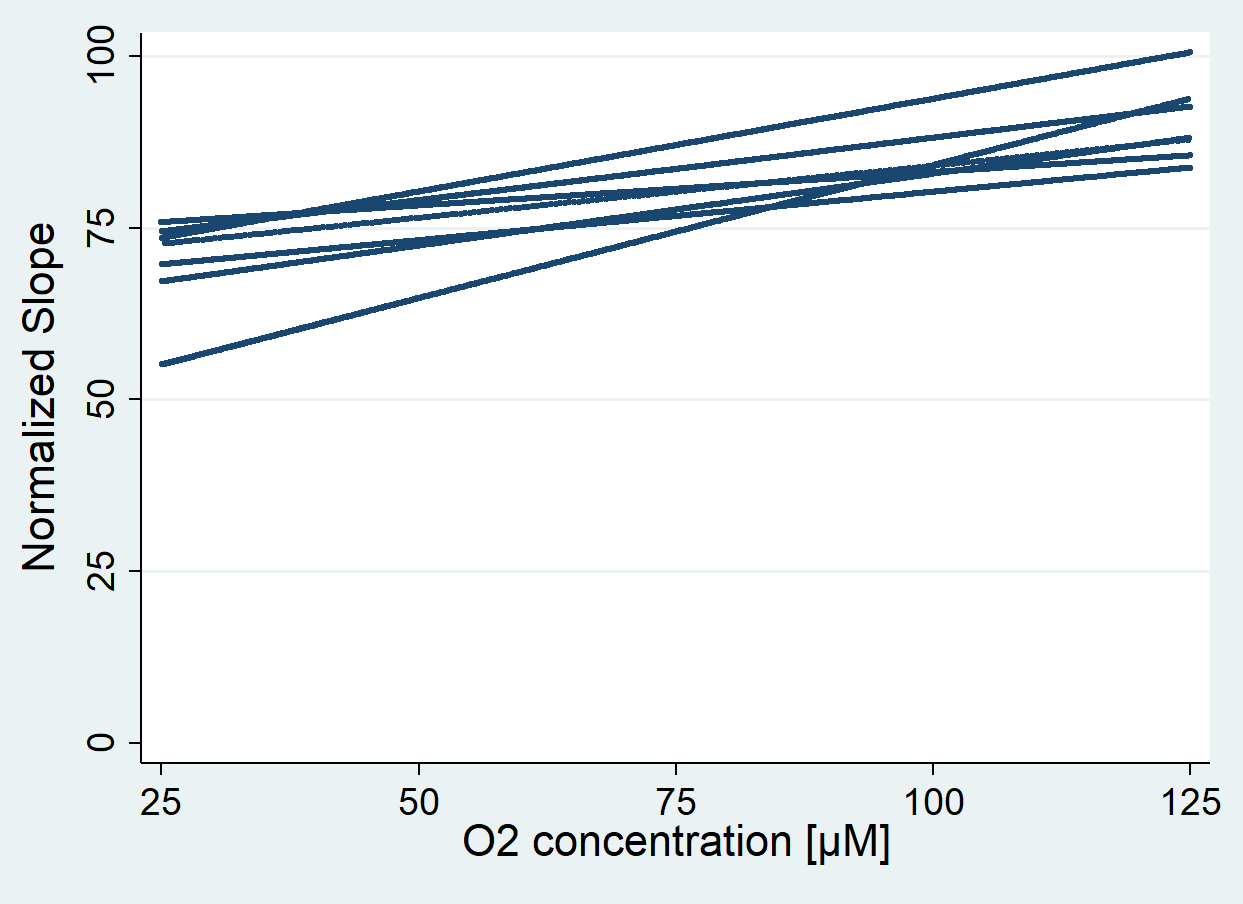


lrtest m1 m2

Model s covariancí random intercept and slope je statisticky signifikantně lepší než bez covariance

Covariance je: -.8904208, tedy záporná, což znamená čím výše je položen intercept tím nižžší je slope.

## Fractional polynomials

fp <O2conc>, : regress normO2slope O2conc <O2conc>

fp plot, residuals(none)


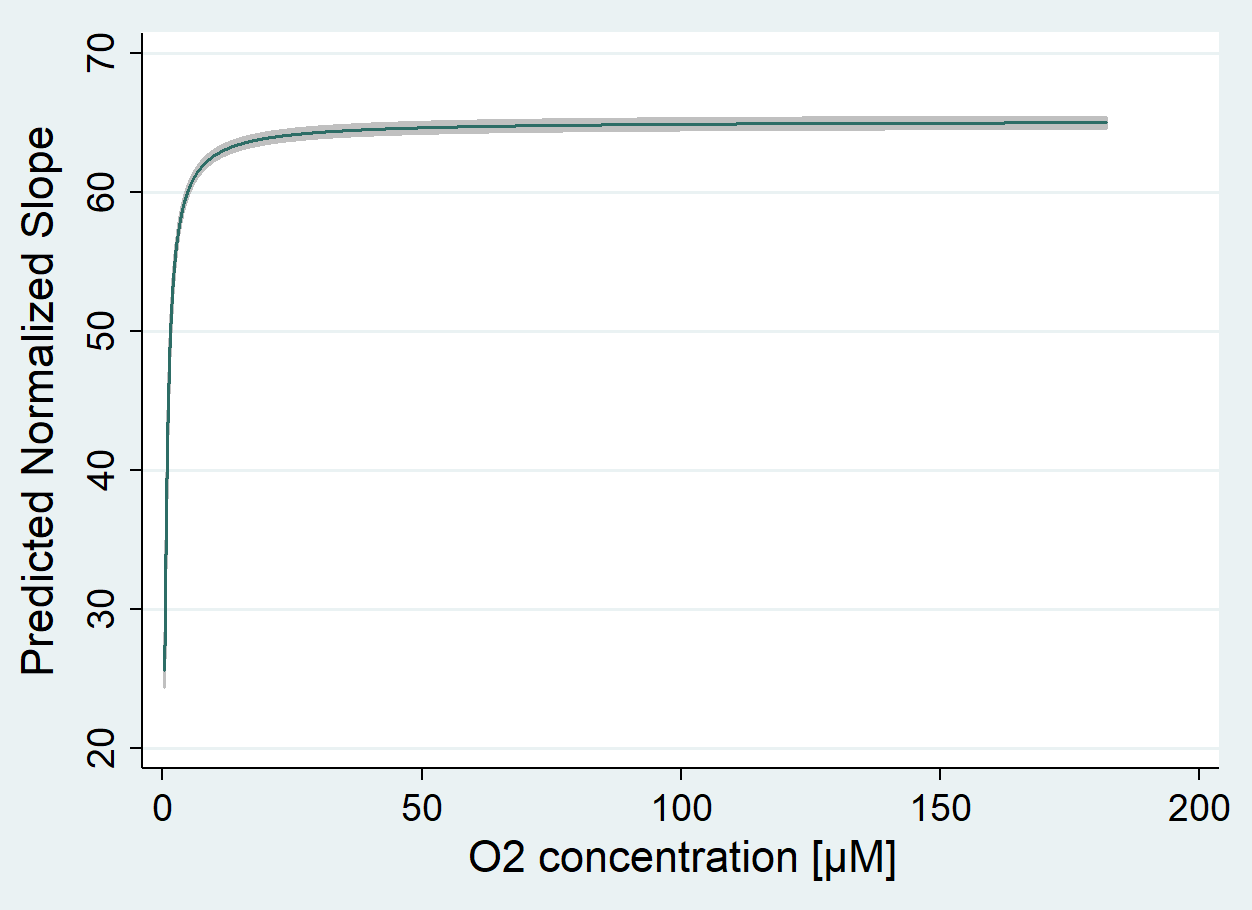


O_2_ slope = 65.1 + 0.2 * O_2_ concentration + $\frac{4.1}{{O2concentration}^{2}}$ - $\frac{25.5}{{O2concentration}}$

R^2^=0.8

### Jednodušší model

fp <O2conc>, fp(-0.5):: regress normO2slope O2conc <O2conc>

fp plot, residuals(none)


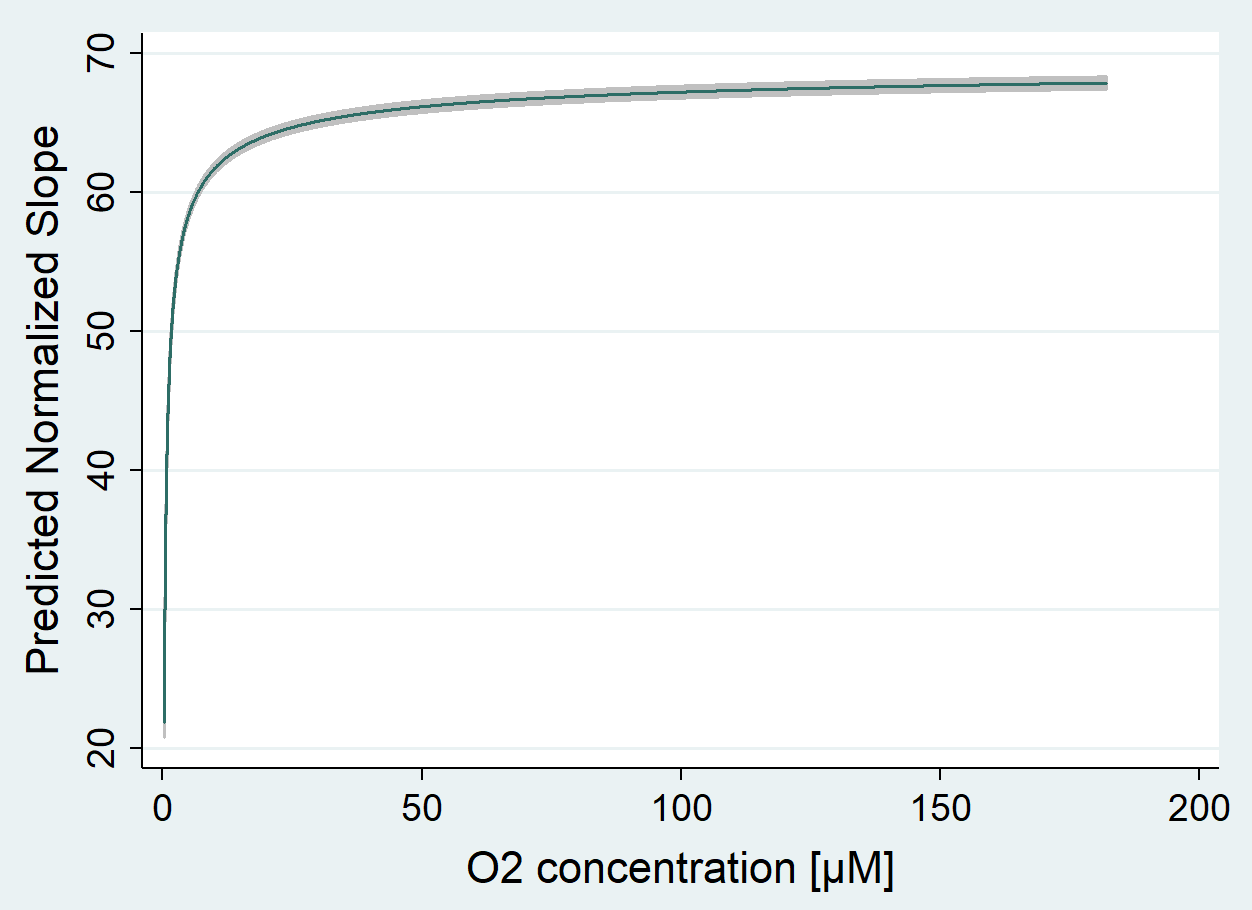


predict normO2sl_pr

predict normO2sl_res, res

fp plot, residuals(residuals)
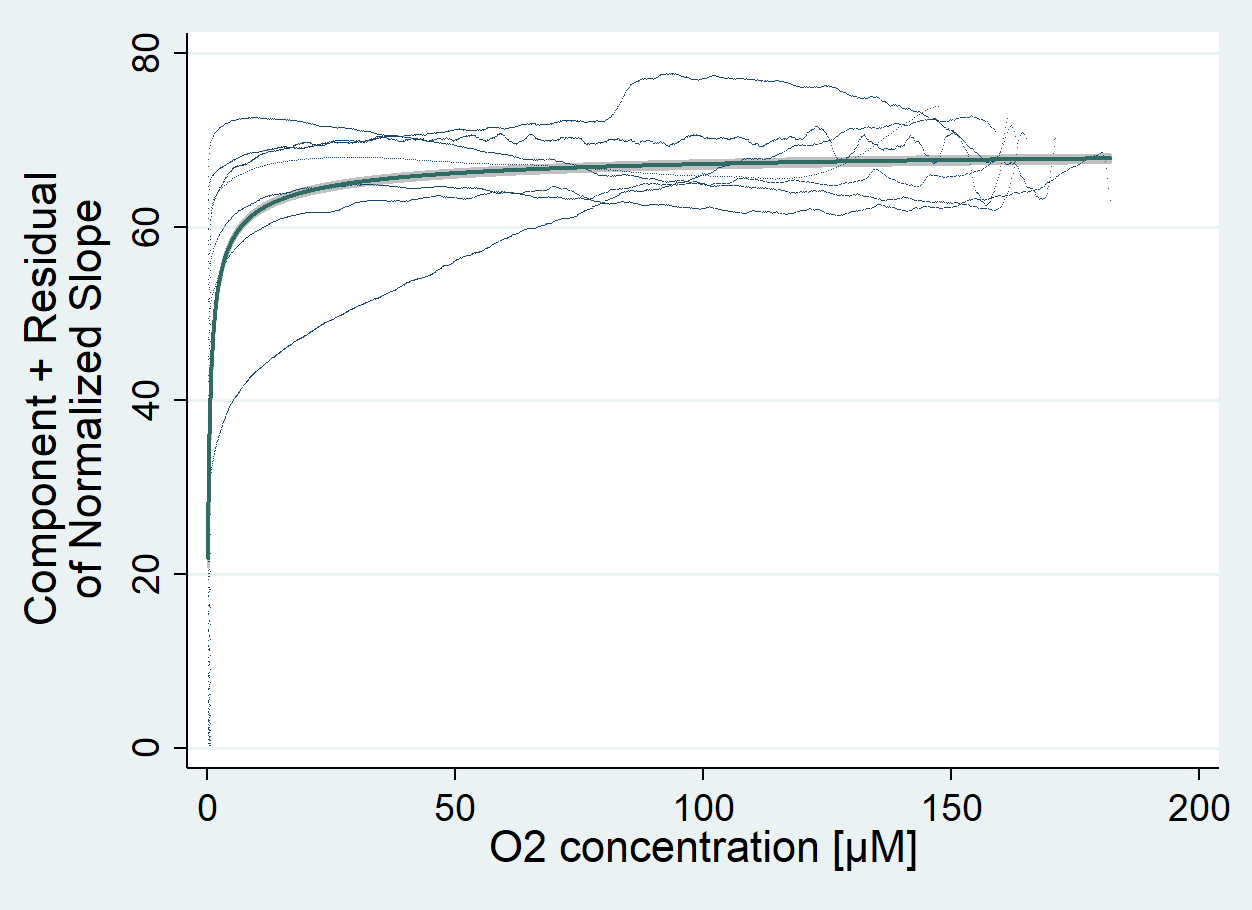


fp plot if O2conc <50, residuals(none)

fp plot if O2conc <25, residuals(none)


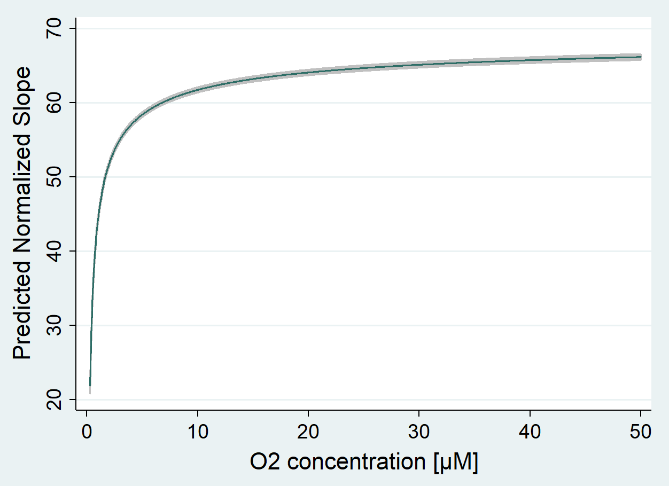

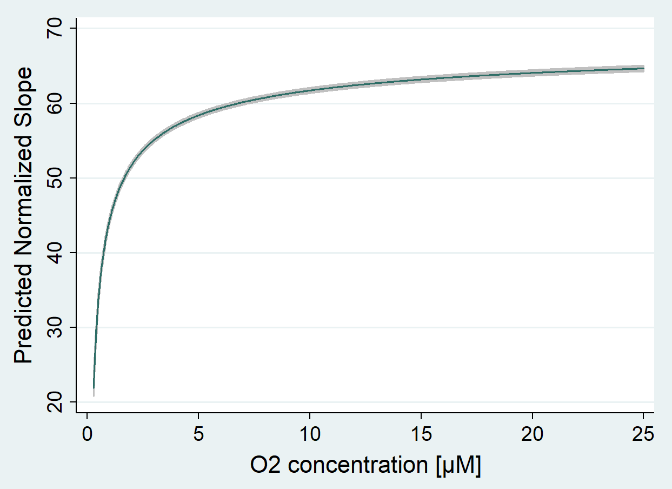


O_2_ slope = 69.7 + 0.18 * O_2_ concentration - $\frac{25.4}{\sqrt{O2\mathrm{concentration}}}$

R^2^=0.8

# R^2^

tabstat r2, stat(N mean sd var cv p25 p50 p75 min max)

graph box r2

# O2 slope neg. [pmol/(s*ml)] post CC

tabstat postcc, stat(N mean sd var cv p25 p50 p75 min max)

graph box postcc

# O2 slope neg. [pmol/(s*ml)] post FCCP

tabstat postfccp , stat(N mean sd var cv p25 p50 p75 min max)

graph box postfccp

Pacient č.1 se v FCCP chová jako outlier

# Slope & FCCP correlation

pwcorr slope postfccp ,sig

R^2^= 0.56, p=0.189

# Slope & CC correlation

pwcorr slope cc,sig

R^2^= 0.67, p=0.094
