# Supplementary material for: High resolution respirometry to assess function of mitochondria in native homogenates of human heart muscle
Source: PLoS One. 2020 Jan 15;15(1):e0226142. doi: 10.1371/journal.pone.0226142 (PMC6961865; doi:10.1371/journal.pone.0226142)
Supplement: S1 Data — (ZIP) [file pone.0226142.s003.zip › Analysis_Titrations.docx]

# Titrace

Petr Waldauf

Stata 14.2

09.04.2017

label define test 1 "FCCP" 2 "ADP" 3 "Oligo"

label values test test

label define substrate 1 "malate" 2 "glutamate" 3 "ADP" 4 "cytochrome C" 5 "succinate" 6 "oligomycine" 7 "FCCP" 8 "antimycine A"

label values substrate substrate

# FCCP

twoway (connected value conc if substrate ==7 & patient ==1, connect(ascending)) (connected value conc if substrate ==7 & patient ==2, connect(ascending)) (connected value conc if substrate ==7 & patient ==3, connect(ascending)) (connected value conc if substrate ==7 & patient ==4, connect(ascending)) (connected value conc if substrate ==7 & patient ==5, connect(ascending)) (connected value conc if substrate ==7 & patient ==6, connect(ascending)) (connected value conc if substrate ==7 & patient ==7, connect(ascending)), ytitle(OCR post FCCP) ytitle(, size(large)) ylabel(, labsize(medlarge)) xtitle(Concentration) xtitle(, size(large)) xlabel(, labsize(medlarge)) legend(order(1 "1" 2 "2" 3 "3" 4 "4" 5 "5" 6 "6" 7 "7") rows(1) title(Pacient, size(medlarge) color(black)))

# FCCP/basalOCR

twoway (connected valueperc conc if substrate ==7 & patient ==1, connect(ascending)) (connected valueperc conc if substrate ==7 & patient ==2, connect(ascending)) (connected valueperc conc if substrate ==7 & patient ==3, connect(ascending)) (connected valueperc conc if substrate ==7 & patient ==4, connect(ascending)) (connected valueperc conc if substrate ==7 & patient ==5, connect(ascending)) (connected valueperc conc if substrate ==7 & patient ==6, connect(ascending)) (connected valueperc conc if substrate ==7 & patient ==7, connect(ascending)), ytitle(FCCP/basalOCR [%]) ytitle(, size(large)) ylabel(, labsize(medlarge)) xtitle(Concentration) xtitle(, size(large)) xlabel(, labsize(medlarge)) legend(order(1 "1" 2 "2" 3 "3" 4 "4" 5 "5" 6 "6" 7 "7") rows(1) title(Pacient, size(medlarge) color(black)))

# ADP

twoway (connected value conc if substrate ==3 & patient ==1, connect(ascending)) (connected value conc if substrate ==3 & patient ==2, connect(ascending)) (connected value conc if substrate ==3 & patient ==3, connect(ascending)) (connected value conc if substrate ==3 & patient ==4, connect(ascending)) (connected value conc if substrate ==3 & patient ==5, connect(ascending)) (connected value conc if substrate ==3 & patient ==6, connect(ascending)) (connected value conc if substrate ==3 & patient ==3, connect(ascending)), ytitle(OCR post ADP) ytitle(, size(large)) ylabel(, labsize(medlarge)) xtitle(Concentration) xtitle(, size(large)) xlabel(, labsize(medlarge)) legend(order(1 "1" 2 "2" 3 "3" 4 "4" 5 "5" 6 "6" 7 "7") rows(1) title(Pacient, size(medlarge) color(black)))

# ADP/glutamate

twoway (connected valueperc conc if substrate ==3 & patient ==1, connect(ascending)) (connected valueperc conc if substrate ==3 & patient ==2, connect(ascending)) (connected valueperc conc if substrate ==3 & patient ==3, connect(ascending)) (connected valueperc conc if substrate ==3 & patient ==4, connect(ascending)) (connected valueperc conc if substrate ==3 & patient ==5, connect(ascending)) (connected valueperc conc if substrate ==3 & patient ==6, connect(ascending)) (connected valueperc conc if substrate ==3 & patient ==3, connect(ascending)), ytitle(ADP/glutamate [%]) ytitle(, size(large)) ylabel(, labsize(medlarge)) xtitle(Concentration) xtitle(, size(large)) xlabel(, labsize(medlarge)) legend(order(1 "1" 2 "2" 3 "3" 4 "4" 5 "5" 6 "6" 7 "7") rows(1) title(Pacient, size(medlarge) color(black)))

# Oligomycine

twoway (connected value conc if substrate ==6 & patient ==1, connect(ascending)) (connected value conc if substrate ==6 & patient ==2, connect(ascending)) (connected value conc if substrate ==6 & patient ==6, connect(ascending)) (connected value conc if substrate ==6 & patient ==4, connect(ascending)) (connected value conc if substrate ==6 & patient ==5, connect(ascending)) (connected value conc if substrate ==6 & patient ==6, connect(ascending)) (connected value conc if substrate ==6 & patient ==6, connect(ascending)), ytitle(OCR post Oligomycine) ytitle(, size(large)) ylabel(, labsize(medlarge)) xtitle(Concentration) xtitle(, size(large)) xlabel(, labsize(medlarge)) legend(order(1 "1" 2 "2" 3 "3" 4 "4" 5 "5" 6 "6" 7 "7") rows(1) title(Pacient, size(medlarge) color(black)))

# Oligomycine/basalOCR

twoway (connected valueperc conc if substrate ==6 & patient ==1, connect(ascending)) (connected valueperc conc if substrate ==6 & patient ==2, connect(ascending)) (connected valueperc conc if substrate ==6 & patient ==6, connect(ascending)) (connected valueperc conc if substrate ==6 & patient ==4, connect(ascending)) (connected valueperc conc if substrate ==6 & patient ==5, connect(ascending)) (connected valueperc conc if substrate ==6 & patient ==6, connect(ascending)) (connected valueperc conc if substrate ==6 & patient ==6, connect(ascending)), ytitle(Oligomycine/basal OCR [%]) ytitle(, size(large)) ylabel(, labsize(medlarge)) xtitle(Concentration) xtitle(, size(large)) xlabel(, labsize(medlarge)) legend(order(1 "1" 2 "2" 3 "3" 4 "4" 5 "5" 6 "6" 7 "7") rows(1) title(Pacient, size(medlarge) color(black)))

# Cytochrome C %

## Data ze všech experimentů (titrace FCCP, ADP, oligo)

Poznámka:

p25 = 25th percentile

p50 = 50th percentile

p75 = 75th percentile

cv = coefficient of variation (sd/mean)

tabstat ccperc, stat (N mean SD var cv p25 p50 p75 min max)

graph box ccperc

Vzestup OCR po přidání CC je v ptůměru 22.1%± 11.3, median 22.4 (16.1;31.5)

### Bez outlieru

tabstat ccperc if ccperc>0, stat (N mean SD var cv p25 p50 p75 min max)

graph box ccperc if ccperc>0

Vzestup OCR po přidání CC je v ptůměru 23.6%± 9.5, median 24.3 (17.0;31.8)

## Data pouze z titrace FCCP

Poznámka:

p25 = 25th percentile

p50 = 50th percentile

p75 = 75th percentile

cv = coefficient of variation (sd/mean)

tabstat ccperc, stat (N mean SD var cv p25 p50 p75 min max)

Vzestup OCR po přidání CC je v ptůměru 24.0%± 13.4, median 28.5 (20.8;34)

graph box ccperc
