# Supplementary figures and images for: High resolution respirometry to assess function of mitochondria in native homogenates of human heart muscle
Source: PLoS One. 2020 Jan 15;15(1):e0226142. doi: 10.1371/journal.pone.0226142 (PMC6961865; doi:10.1371/journal.pone.0226142)

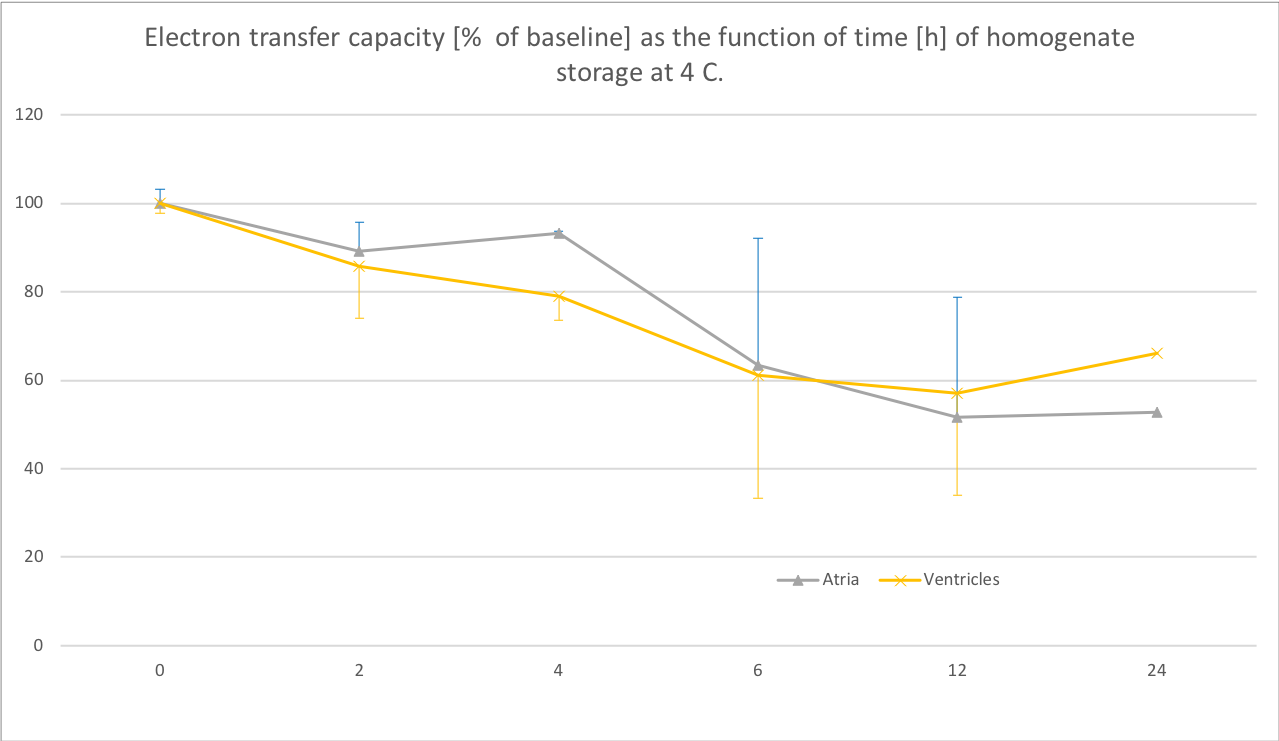

Supplement: S1 Data — (ZIP) [file pone.0226142.s003.zip › Durability_of_homogenates-ETC capacity.png]

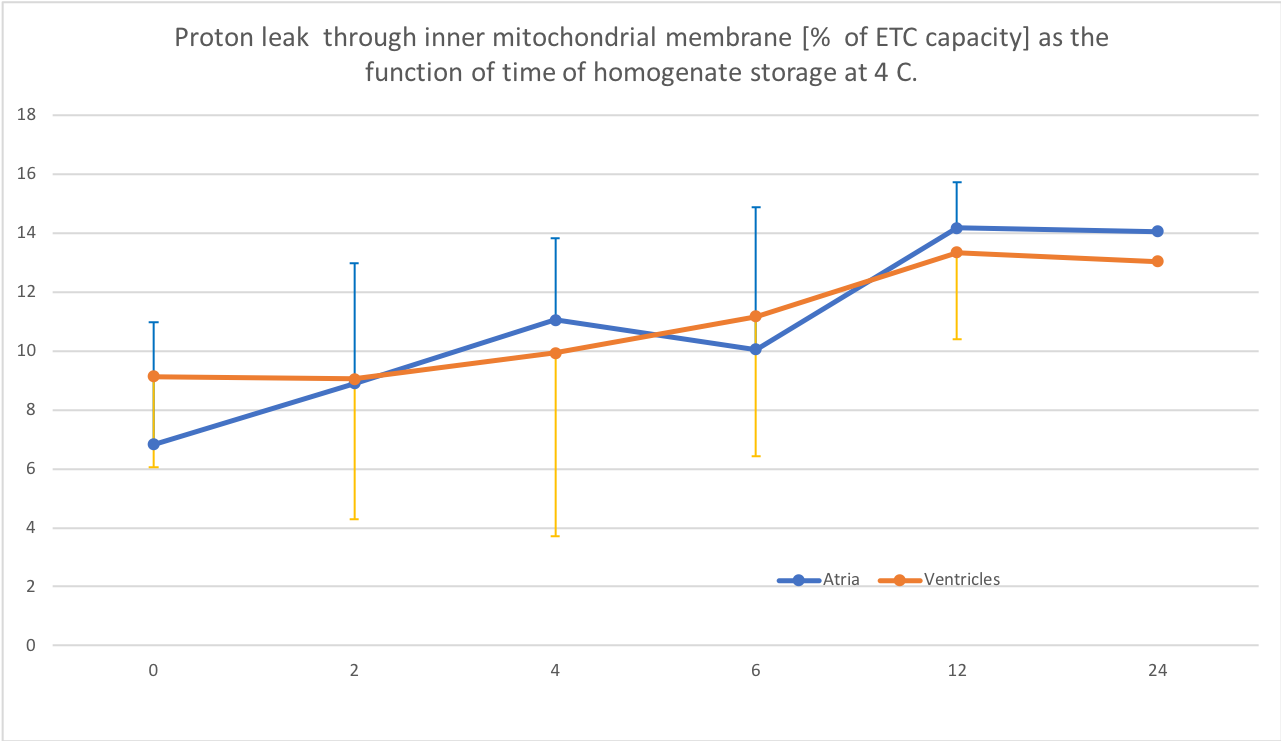

Supplement: S1 Data — (ZIP) [file pone.0226142.s003.zip › Durability_of_homogenates-LEAK.png]
